# Genetically Modified Crops and Food Security

**DOI:** 10.1371/journal.pone.0064879

**Published:** 2013-06-05

**Authors:** Matin Qaim, Shahzad Kouser

**Affiliations:** 1 Department of Agricultural Economics and Rural Development, Georg-August-University of Goettingen, Goettingen, Germany; 2 Institute of Agricultural and Resource Economics, University of Agriculture, Faisalabad, Pakistan; TGen, United States of America

## Abstract

The role of genetically modified (GM) crops for food security is the subject of public controversy. GM crops could contribute to food production increases and higher food availability. There may also be impacts on food quality and nutrient composition. Finally, growing GM crops may influence farmers’ income and thus their economic access to food. Smallholder farmers make up a large proportion of the undernourished people worldwide. Our study focuses on this latter aspect and provides the first *ex post* analysis of food security impacts of GM crops at the micro level. We use comprehensive panel data collected over several years from farm households in India, where insect-resistant GM cotton has been widely adopted. Controlling for other factors, the adoption of GM cotton has significantly improved calorie consumption and dietary quality, resulting from increased family incomes. This technology has reduced food insecurity by 15–20% among cotton-producing households. GM crops alone will not solve the hunger problem, but they can be an important component in a broader food security strategy.

## Introduction

Food security exists when all people have physical and economic access to sufficient, safe, and nutritious food. Unfortunately, food security does not exist for a significant proportion of the world population. Around 900 million people are undernourished, meaning that they are undersupplied with calories [1]. Many more suffer from specific nutritional deficiencies, often related to insufficient intake of micronutrients. Eradicating hunger is a central part of the United Nations’ Millennium Development Goals [2]. But how to achieve this goal is debated controversially. Genetically modified (GM) crops are sometimes mentioned in this connection. Some see the development and use of GM crops as key to reduce hunger [3], [4], while others consider this technology as a further risk to food security [5], [6]. Solid empirical evidence to support either of these views is thin.

There are three possible pathways how GM crops could impact food security. First, GM crops could contribute to food production increases and thus improve the availability of food at global and local levels. Second, GM crops could affect food safety and food quality. Third, GM crops could influence the economic and social situation of farmers, thus improving or worsening their economic access to food. This latter aspect is of particular importance given that an estimated 50% of all undernourished people worldwide are small-scale farmers in developing countries [7].

In regard to the first pathway, GM technologies could make food crops higher yielding and more robust to biotic and abiotic stresses [8], [9]. This could stabilize and increase food supplies, which is important against the background of increasing food demand, climate change, and land and water scarcity. In 2012, 170 million hectares (ha) – around 12% of the global arable land – were planted with GM crops, such as soybean, corn, cotton, and canola [10], but most of these crops were not grown primarily for direct food use. While agricultural commodity prices would be higher without the productivity gains from GM technology [11], impacts on food availability could be bigger if more GM food crops were commercialized. Lack of public acceptance is one of the main reasons why this has not yet happened more widely [12].

Concerning the second pathway, crops with new traits can be associated with food safety risks, which have to be assessed and managed case by case. But such risks are not specific to GM crops. Long-term research confirms that GM technology is not *per se* more risky than conventional plant breeding technologies [13]. On the other hand, GM technology can help to breed food crops with higher contents of micronutrients; a case in point is Golden Rice with provitamin A in the grain [14]. Such GM crops have not yet been commercialized. Projections show that they could reduce nutritional deficiencies among the poor, entailing sizeable positive health effects [15], [16].

The third pathway relates to GM crop use by smallholder farmers in developing countries. Half of the global GM crop area is located in developing countries, but much of this refers to large farms in countries of South America. One notable exception is *Bacillus thuringiensis* (Bt) cotton, which is grown by around 15 million smallholders in India, China, Pakistan, and a few other developing countries [10]. Bt cotton provides resistance to important insect pests, especially cotton bollworms. Several studies have shown that Bt cotton adoption reduces chemical pesticide use and increases yields in farmers’ fields [17]–[20]. There are also a few studies that have shown that these benefits are associated with increases in farm household income and living standard [21]–[23]. Higher incomes are generally expected to cause increases in food consumption in poor farm households. On the other hand, cotton is a non-food cash crop, so that the nutrition impact is uncertain.

Here we address this question and analyze the impact of Bt cotton adoption on calorie consumption and dietary quality in India. Bt cotton was first commercialized in India in 2002. In 2012, over 7 million farmers had adopted this technology on 10.8 million ha – equivalent to 93% of the country’s total cotton area [10]. For the analysis, we carried out a household survey and collected comprehensive data over a period of several years. This is the first *ex post* study that analyzes food security effects of Bt cotton or any other GM crop with micro level data.

## Materials and Methods

### Ethics Statement

Our study builds on data from a socioeconomic survey of farm households in India. Details of this survey are explained further below. The institutional review board of the University of Goettingen only reviews clinical research; our study cannot be classified as clinical research. We consulted with the Head of the Research Department of the University of Goettingen, who confirmed that there is no institutional review board at our University that would require a review of such survey-based socioeconomic research.

### Farm Household Survey

We carried out a panel survey of Indian cotton farm households in four rounds between 2002 and 2008. We used a multistage sampling procedure. Four states were purposively selected, namely Maharashtra, Karnataka, Andhra Pradesh, and Tamil Nadu. These four states cover a wide variety of different cotton-growing situations, and they produce 60% of all cotton in central and southern India [23]. In these four states, we randomly selected 10 cotton-growing districts and 58 villages, using a combination of census data and agricultural production statistics [18], [19], [23]. Within each village, we randomly selected farm households from complete lists of cotton producers. Sample households were visited individually, and the household head was taken through a face-to-face interview, for which we used a structured questionnaire. The questionnaire covered a wide array of agricultural and socioeconomic information, such as input-output details in cotton production, technology adoption, other income sources, and household living standards. The interviews were carried out in local languages by a small team of enumerators, who were trained and supervised by the researchers.

Prior to starting each interview, the study objective was explained. We also clarified that the data collected would be treated confidentially, analyzed anonymously, and be used for research purposes only. Based on this, the interviewees were asked for their verbal informed consent to participate. We decided not ask for written consent, because the interviews were not associated with any risk for participants. Furthermore, many of the sample farmers had relatively low educational backgrounds and were not used to formal paperwork. Very few households did not agree to participate; they were replaced with other randomly selected households in the same villages.

The first-round survey interviews took place in early 2003, shortly after the cotton harvest for the 2002 season was completed. The same survey was repeated at two-year intervals in early 2005 (referring to the 2004 cotton season), early 2007 (referring to the 2006 season), and early 2009 (referring to the 2008 season). In total, 533 households were interviewed during the 7-year period. Most of these households were visited in several rounds. The total sample consists of 1431 household observations (Table 1). In 2002, the proportion of Bt adopters was still relatively small, but it increased rapidly in the following years. By 2008, 99% of the sample households had adopted this technology. To our knowledge, this is the only longer-term panel survey of Bt cotton farm households in a developing country (the data set with the variables used in this article is available as Data S1).

**Table 1:** Number of farm households sampled in India in four survey rounds.

Farm households	2002	2004	2006	2008	Total
Adopters of Bt	131	246	333	375	1085
Non-adopters of Bt	210	117	14	5	346
Total	341	363	347	380	1431

### Calorie Consumption Data

The survey questionnaire included a detailed food consumption recall, which is a common tool to assess food security at the household level [24]. For a 30-day recall period, households were asked about the quantity consumed of different food items and the corresponding monetary value. The questions covered food consumed from own production, market purchases, gifts, and transfers.

The quantity data for the different food items were converted to calories consumed by using calorie conversion factors for India [25], [26]. The total household calorie consumption from the 30-day recall was then divided by 30 to obtain a calorie value per day. Taking into account the age and gender structure of households, as well as physical activity levels of household members, the number of adult equivalents (AE) was calculated for each household. Male adults involved in farming count as 1.0 AE, female adults involved in farming as 0.8 AE. Male and female adults with lower physical activity levels count as 0.8 and 0.7, respectively. For children and adolescents, appropriate adjustments were made [25]–[27]. The daily household calorie consumption was divided by the number of AE in a household to obtain the calories consumed per AE and day.

Values for minimum dietary energy requirements found in the literature vary, which is due to several reasons [24]. Values stated per capita are lower than those stated per AE, because children have lower calorie requirements than adults. Moreover, not all studies take physical activity levels into account already in the AE calculations, as we do. The average daily calorie requirement for a moderately active AE in India is 2875 kcal/day [25]. According to the World Health Organization, a safe minimum daily intake should not fall below 80% of the calorie requirement, meaning 2300 kcal per AE. Minimum values around 2300 kcal per day for adult men are also found in other studies [28]. Based on this, we take 2300 kcal per AE as the threshold, that is, households with daily calorie consumption below 2300 kcal per AE are considered food insecure.

Most of the calories consumed in rural India are from cereals such as wheat, rice, millet, and sorghum that are rich in carbohydrates but less nutritious in terms of protein and micronutrient contents. Hence, in addition to total calories consumed we calculated the number of calories consumed from more nutritious foods to assess dietary quality. In the category “more nutritious foods”, we include pulses, fruits, vegetables, and all animal products (i.e., milk, milk products, meat, fish, and eggs). Recent research suggests that the share of calories consumed from higher value, non-staple foods can also be used as an indicator of nutritional sufficiency [29]. The reason is that poor and undernourished households will largely choose foods that are the cheapest available sources of calories, namely cereals in the context of rural India. Only when they have surpassed subsistence, consumers will begin to substitute towards foods that are more expensive sources of calories [29].

It should be mentioned that food consumption data from household surveys may not provide very accurate data to measure nutritional status [24], [30]. Sometimes, consumption data overestimate calorie intakes, because food losses, waste, and other uses within the household cannot be properly accounted for. However, this limitation applies to both adopters and non-adopters of Bt, so that the comparison between Bt and non-Bt, which is relevant for the impact assessment, is unaffected.

### Regression Models

To estimate the impact of Bt cotton adoption on calorie consumption, we regress total daily calorie consumption per AE on Bt adoption, measured as the number of hectares of Bt cotton grown by a household in a particular year. Since Bt adoption increases farm profits and household incomes [23], we expect a positive and significant treatment effect. However, calorie consumption is also influenced by other factors that need to be controlled for. We control for education of the household head (measured in terms of the number of years of schooling); education plays an important role for both income generation and consumption behavior. We also include a variable for household size (measured in terms of AE). Moreover, we control for farm size in terms of area owned, which is a proxy for agricultural asset ownership more generally. Farm income is not included in the model, as this is directly influenced by Bt adoption. However, off-farm income, measured in US$ per year, is controlled for. We also include state dummies for Karnataka, Andhra Pradesh, and Tamil Nadu (Maharashtra is the reference state), capturing climatic and agroecological differences. Given the panel structure of the data with four survey rounds, we use year dummies for 2004, 2006, and 2008 (2002 is the reference year).

Panel data models are often estimated with a random effects estimator [31]. However, a random effects estimator can lead to biased impact estimates when there is unobserved heterogeneity between Bt adopting and non-adopting households. Such bias resulting from endogeneity of the treatment variable is referred to as selection bias in the impact assessment literature [23], [31]. Unobserved heterogeneity may potentially result from differences in household characteristics (e.g., Bt adopting farmers may have higher motivation, better management skills, or better access to information) or farm characteristics (e.g., differences in soil quality, or water access). Our panel data allow us to control for such unobserved heterogeneity. Since we surveyed the same households repeatedly over a 7-year period when Bt adoption increased, for many households we have observations with and without Bt adoption. Hence, we rely on a within household estimator, which is also called a fixed effects estimator. Differencing within households with the fixed effects estimator eliminates time-invariant unobserved factors, so that they can no longer bias the impact estimates [31]. A Hausman test is used to confirm the appropriateness of the fixed effects specification [19], [31].

We estimate an additional model using calories from more nutritious foods (i.e., pulses, fruits, vegetables, and animal products) instead of total calorie consumption as dependent variable. This additional model helps to analyze impacts of Bt cotton adoption on dietary quality. A positive coefficient for the treatment variable would indicate that Bt adoption increases the consumption of more nutritious foods, thus not only contributing to more calories but also to better dietary quality.

## Results and Discussion

Descriptive statistics are shown in Table 2. The average farm household owns 5 ha of land, without a significant difference between Bt adopters and non-adopters. Around half of this area is grown with cotton. Other crops cultivated include wheat, millet, sorghum, pulses, and in some locations rice, among others. Households are relatively poor; average annual per capita consumption expenditures range between 300 and 500 US$.

**Table 2:** Descriptive statistics of farm households.

Variables	Adopters of Bt (N = 1085)	Non-adopters of Bt (N = 346)
Farm size (ha)	5.11 (5.85)	4.85 (5.51)
Cotton area cultivated (ha)	2.35 (2.35)	2.79 (19.67)
Area cultivated with Bt cotton (ha)	1.97*** (2.08)	0.00 (0.00)
Age of farmer (years)	45.58 (12.86)	45.94 (12.36)
Education of farmer (years)	7.58*** (4.94)	6.69 (5.03)
Per capita consumption expenditure (US$/year)	490.31*** (430.18)	311.72 (355.58)
Off-farm income (US$/year)	560.70 (1455.44)	504.27 (2289.87)
Calorie consumption per AE (kcal/day)	3329.41*** (719.38)	2829.88 (598.99)
Calories consumed from more nutritious foods per AE (kcal/day)^a^	703.89*** (374.90)	638.89 (345.41)
Household size (AE)	5.01 (2.42)	5.14 (2.24)
Food insecure households (%)^b^	7.93***	19.94

Mean values are shown with standard deviations in parentheses. N: Number of observations; AE: adult equivalent.

*** Mean values between adopters and non-adopters of Bt are statistically significant at the 1% level.

a More nutritious foods include pulses, fruits, vegetables, and all animal products.

b Consumption of less than 2300 kcal per AE and day.

**Figure 1:**
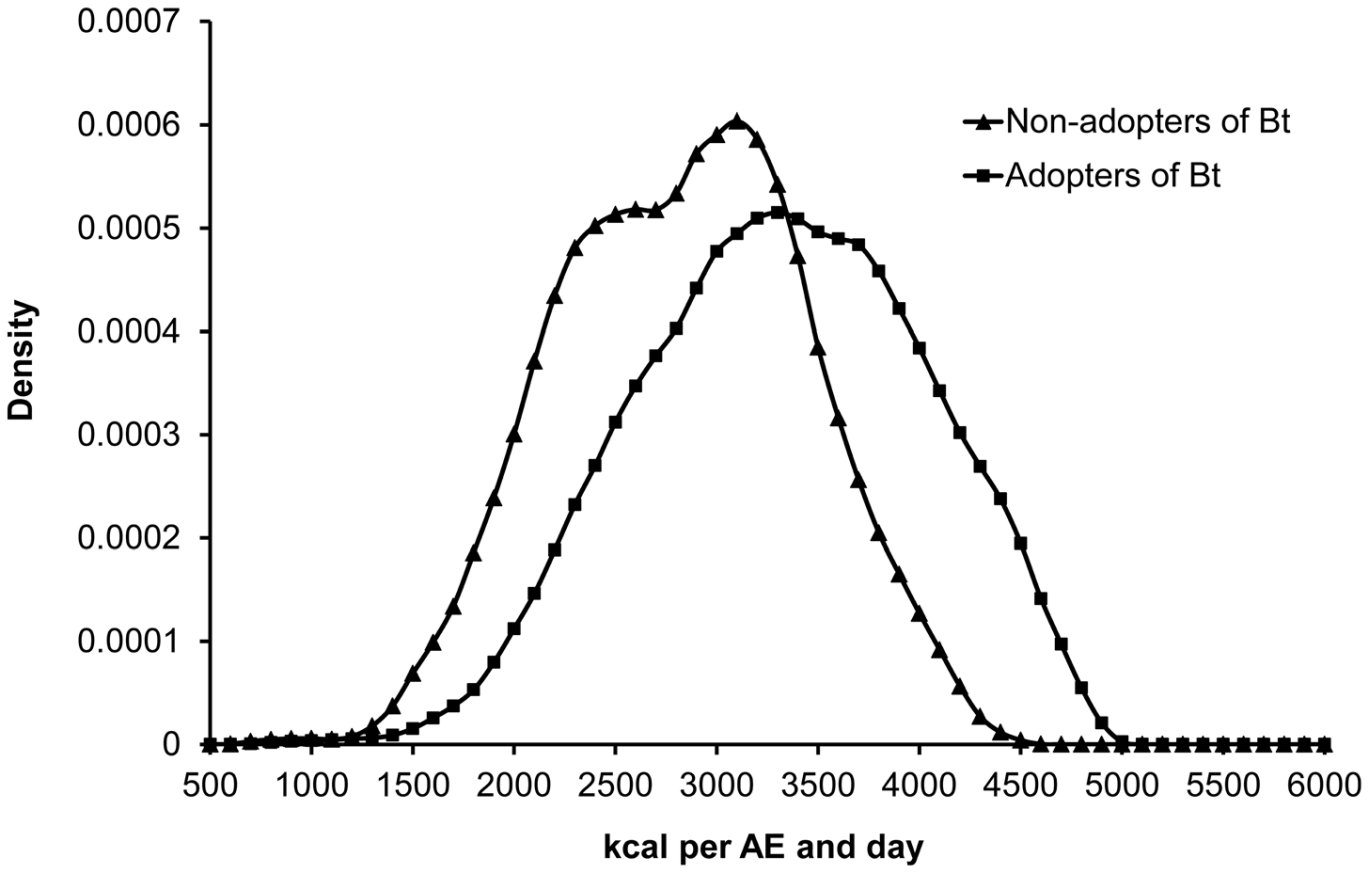
Density functions of household calorie consumption for adopters and non-adopters of Bt cotton. Functions were estimated non-parametrically using the Epanechnikov kernel with 1085 and 346 observations for adopting and non-adopting households, respectively. AE: adult equivalent.

Bt adopting households consume significantly more calories than non-adopting households, and a smaller proportion of them is food insecure (Figure 1, Table 2). This suggests that the cash income gains through Bt adoption may have improved food security among cotton-producing households. Yet, this simple comparison does not yet prove a causal relationship.

### Impact of Bt Cotton Adoption on Food Security

To further analyze the relationship between Bt adoption and calorie consumption, we use panel regression models, as explained above. The main explanatory variable of interest is the Bt cotton area of a farm household, for which descriptive statistics are shown in Table 3. The average Bt area among technology adopters in the sample is close to 2 ha, which is equivalent to 85% of the total cotton area of these farms. A breakdown by survey year shows that the average Bt area increased from less than 1.0 ha in 2002 to 2.4 ha in 2008. Hence, not only the number of Bt adopters but also the Bt area per adopting household increased considerably over time.

**Table 3:** Bt cotton area among adopting households.

	2002	2004	2006	2008	Total
Mean Bt area (ha)	0.94	1.64	2.15	2.37	1.97
Standard deviation	1.32	1.87	2.14	2.22	2.08
Number of observations	131	246	333	375	1085

**Figure 2:**
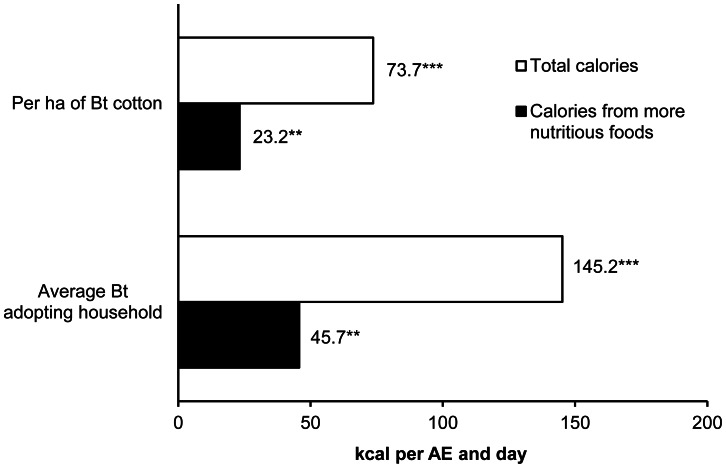
Net effects of Bt adoption on household calorie consumption. Results based on calorie consumption regression models estimated with panel data and household fixed effects (within estimator). Full model results are shown in Table 4. Calories from more nutritious foods include pulses, fruits, vegetables, and animal products. Effects for the average adopting household take into account the number of ha of Bt cotton actually grown. **Significant at the 5% level. ***Significant at the 1% level.

The regression results are shown in Table 4. Each ha of Bt cotton has increased total calorie consumption by 74 kcal per AE and day. For the average adopting household, the net effect is 145 kcal per AE (Figure 2), implying a 5% increase over mean calorie consumption in non-adopting households. Most of the calories consumed in rural India stem from cereals that are rich in carbohydrates but less nutritious in terms of protein and micronutrients. Yet the results show that Bt adoption has significantly increased the consumption of calories from more nutritious foods, thus also contributing to improved dietary quality.

**Table 4:** Calorie consumption models.

	Model (1)	Model (2)	Model (3)
Variables	Total calories (RE model)	Total calories (FE model)	Calories from more nutritious foods (FE model)
Bt area (ha)	79.08*** (18.85)	73.71*** (21.40)	23.17** (10.05)
Farm size (ha)	9.27** (4.22)	−0.69 (7.80)	1.97 (3.56)
Education of farmer (years)	9.41** (4.40)	–	–
Off-farm income (US$/year)	0.07*** (0.02)	0.05*** (0.02)	0.01* (0.007)
Household size (AE)	−62.48*** (10.71)	−89.46*** (14.43)	−29.33*** (6.89)
Karnataka (dummy)^a^	88.36 (57.97)	–	–
Andhra Pradesh (dummy)^a^	21.46 (58.00)	–	–
Tamil Nadu (dummy)^a^	212.86** (84.56)	–	–
2004 (dummy)^b^	−34.35 (48.97)	−5.98 (51.60)	−45.25* (25.33)
2006 (dummy)^b^	13.68 (54.48)	30.09 (61.12)	−112.87*** (29.41)
2008 (dummy)^b^	−92.92 (60.51)	−74.59 (69.51)	−72.70** (30.20)
Constant	3229.31*** (90.46)	3537.08*** (78.16)	843.23*** (41.42)
Number of observations	1431	1431	1431
R^2^	0.13	0.09	0.10
Hausman test (chi-square statistic)	16.82**		

The dependent variable in models (1) and (2) is the total number of kcal consumed per AE and day. The dependent variable in model (3) is the number of kcal consumed from more nutritious foods (i.e., pulses, fruits, vegetables, and animal products) per AE and day. All coefficient estimates can be interpreted as marginal effects; robust standard errors are shown in parentheses. AE: adult equivalent; RE: random effects; FE: fixed effects.

* , **, ***Significant at the 10%, 5%, and 1% level, respectively.

a The reference state is Maharashtra.

b The reference year is 2002.

We applied the total calorie consumption effect of Bt to the subsample of non-adopters to simulate the food security impact of adoption: if all non-adopters switched to Bt, the proportion of food insecure households would drop by 15–20% (Table 5). Most of these nutritional benefits have materialized already, as over 90% of all cotton farm households in India have adopted Bt technology by now.

**Table 5:** Impact of Bt adoption on food security among cotton-producing households.

	Food insecure households (%)^a^	Change in food insecurity relative to status quo (%)
Non-adopters of Bt cotton (status quo)	19.94	
If non-adopters adopted Bt on their total cotton area	15.90	−20.26
If non-adopters adopted Bt on 85% of their cotton area	16.76	−15.95

The proportion of food insecure households in the status quo refers to the subsample of 346 non-adopters. For these households, changes in calorie consumption through Bt adoption were simulated, assuming full Bt adoption (on 100% of their cotton area) and partial Bt adoption (on 85% of their cotton area, as observed in the subsample of Bt adopters). For the simulations, the net effect of Bt on total calorie consumption per ha was used (Figure 2).

a Consumption of less than 2300 kcal per adult equivalent and day.

### Robustness Checks

We tested the robustness of the Bt effects by estimating calorie consumption models with alternative specifications. These additional estimates are shown in Table 6. We first look at possible changes in impact over time. In model (1), the Bt area variable is split into two periods, namely 2002–04 and 2006–08. In both periods, the Bt impact on calorie consumption was positive and significant, but the effect was bigger in 2002–04 than in 2006–08. The reason for this change is not that income effects of Bt adoption would shrink; recent research showed that the profit gains of Bt cotton in India were constant or even increased over time [23]. The change in the calorie effect per ha of Bt is rather due to the fact that the Bt area per farm increased considerably in the later period, as was shown above. Measured per farm household, the calorie consumption effect of Bt was actually very similar in 2002–04 and 2006–08.

**Table 6:** Robustness checks of Bt effects with different model specifications.

	Model (1)	Model (2)	Model (3)	Model (4)
Variables	Total calories	Calories from more nutritious foods	Total calories	Total calories
Bt area 2002–04 (ha)	135.25*** (28.95)	17.94 (13.24)	–	–
Bt area 2006–08 (ha)	54.67** (23.33)	24.79** (10.46)	–	–
Cumulative Bt area (ha)	–	–	17.20 (12.20)	−28.08** (13.21)
Bt area (ha)	–	–	–	105.63*** (26.82)
Number of observations	1431	1431	1431	1431
	**Model (5)**	**Model (6)**	**Model (7)**	**Model (8)**
	**Total calories**	**Total calories**	**Total calories**	**Total calories**
Bt area (ha)	73.71*** (21.40)	76.19*** (27.62)	110.01*** (27.48)	53.40* (30.99)
Bt (dummy)	–	–	–	599.84*** (70.29)
Number of observations a	1431	1016	852	852

All models are estimated with household fixed effects. Other explanatory variables were included in estimation, as in Table 4, but are not shown here for brevity. The dependent variable in all models is calorie consumption measured in kcal per AE and day. Coefficient estimates can be interpreted as marginal effects; robust standard errors are shown in parentheses.

* , **, ***Significant at the 10%, 5%, and 1% level, respectively.

a In model (6), all observations of households that had adopted Bt in 2002 were dropped. In models (7) and (8), all observations of households that had adopted Bt in all survey rounds were dropped.

The smaller calorie consumption effect per ha of Bt with an increasing Bt area on a farm is consistent with Engel’s law, which states that the proportion of the household budget spent on food decreases as income rises [32]. Unsurprisingly, the same trend is not observed when we focus on higher value, non-staple foods. The results of model (2) in Table 6 suggest that the Bt effect on calories from more nutritious foods has been increasing over time. Hence, Bt cotton adoption leads to a lower staple calorie share, implying higher nutritional sufficiency and better dietary quality [29].

In model (3) of Table 6, we analyze whether the Bt effect is cumulative, meaning that households that have adopted Bt earlier or on larger areas benefit over-proportionally. This might be the case when profit gains from Bt adoption are reinvested, possibly entailing larger consumption benefits in subsequent periods. To test for this option, we constructed a cumulative Bt area variable, adding up the Bt area on a farm in a particular year and Bt areas on the same farm in previous survey rounds. The coefficient of this variable is insignificant; cumulative effects do not seem to be important. If we include this variable together with the standard Bt area variable, the cumulative coefficient turns negative while the actual treatment effect increases (model 4). Again, this is consistent with Engel’s law, implying that larger areas with Bt lead to lower proportions of the income gains being spent on calories.

In models (6) and (7), we analyze to what extent changes in the sample affect the estimation results. For easy comparison, results from the full-sample reference model, which were discussed above, are repeated in model (5). It is sometimes observed that early adopters of a new technology benefit more than late adopters. This may be due to cumulative effects, which we already tested for. In addition, general equilibrium adjustments may contribute to differential impacts between early and late adopters [33]. In model (6), we exclude all households that had adopted Bt already in the first survey round in 2002. The change in the Bt effect is very small, so we conclude that late adopters enjoy the same nutritional benefits per ha of Bt as early adopters.

This specification in model (6) with early adopters excluded is also an additional robustness check for possible issues of endogeneity and selection bias. The fixed effects panel estimator controls for time-invariant heterogeneity between adopters and non-adopters of Bt. But it cannot control for possible time-variant differences, which might play a role if early adopters are more innovative also with respect to other opportunities not captured in our data. The similarity of the results in models (5) and (6) substantiates that the estimated Bt impacts do not suffer from selection bias. In model (7), we exclude all observations of households that had adopted Bt in all survey rounds, so that the results are purely based on within household comparisons. The treatment effect remains highly significant. It even increases in magnitude, suggesting that the full-sample result is rather a cautious, lower-bound estimate. Finally, model (8) includes a dummy for Bt adoption in addition to the Bt area variable used before. The dummy produces a large coefficient, underlining the positive food security impact of Bt adoption. But the Bt area effect remains positive and significant, too, which confirms that using a continuous treatment variable is appropriate.

Overall, the additional results with alternative specifications strengthen the findings and show that the positive impacts of Bt cotton adoption on food security in India are very robust.

### Conclusions

The results of this research confirm that the income gains through Bt cotton adoption among smallholder farm households in India have positive impacts on food security and dietary quality. GM crops are not a panacea for the problems of hunger and malnutrition. Complex problems require multi-pronged solutions. But the evidence suggests that GM crops can be an important component in a broader food security strategy. So far, food security impacts are still confined to only a few concrete examples. The nutritional benefits could further increase with more GM crops and traits becoming available in the future. Appropriate policy and regulatory frameworks are required to ensure that the needs of poor farmers and consumers are taken into account and that undesirable social consequences are avoided.

## Supporting Information

Data S1 (PDF)
