# Supplementary material for: Genetically Modified Crops and Food Security
Source: PLoS One. 2013 Jun 5;8(6):e64879. doi: 10.1371/journal.pone.0064879 (PMC3674000; doi:10.1371/journal.pone.0064879)
Supplement: Data S1 — (PDF) [file pone.0064879.s001.pdf]

obs	hh code	round	year	state	age	edu	off_incUS	hhexpend_pc	hhsize_ae	area_ha	cotarea_ha	bt	bt_ha	kcal_tot	kcal_nutr
1	1	1	2002	1	40	15	193.4236	785.4442	6.7	6.0705	1.41645	1	0.4047	4252	1554.402
2	1	2	2004	1	42	15	2210.555	361.0505	12.1	6.0705	3.2376	1	3.2376	3405	602.8553
3	1	3	2006	1	44	15	552.6389	491.1221	9.2	6.4752	2.8329	1	2.8329	3398	578.6755
4	1	4	2008	1	46	15	921.0648	689.5914	13.9	6.0705	2.8329	1	2.8329	3926	476.3335
5	2	1	2002	1	32	10	3684.259	1257.267	3.8	6.0705	4.047	1	0.8094	4333	866.5029
6	2	2	2004	1	34	10	1473.704	649.2133	9.3	14.1645	6.0705	1	6.0705	3645	1020.911
7	2	3	2006	1	36	10	5526.389	507.3353	9.8	12.141	6.0705	1	6.0705	3253	622.1493
8	2	4	2008	1	38	10	1105.278	467.5858	4.1	3.2376	0.8094	1	0.8094	2236	770.6008
9	3	1	2002	1	45	10	0	359.7391	4.3	4.4517	0.4047	1	0.4047	3652	537.2404
10	3	2	2004	1	47	10	276.3194	254.593	5.2	4.047	1.6188	1	1.2141	3544	507.505
11	3	3	2006	1	49	10	221.0555	344.2995	5	4.047	1.6188	1	1.6188	3117	800.9427
12	3	4	2008	1	51	10	1105.278	448.4207	3.6	4.047	0.60705	1	0.60705	2928	831.6838
13	4	1	2002	1	35	12	3315.833	492.9914	6.7	8.9034	1.2141	1	1.2141	3788	765.2245
14	4	2	2004	1	37	12	0	188.256	6.5	2.4282	0.8094	0	0	2042	372.7124
15	5	1	2002	1	55	0	331.5833	455.7058	3.8	10.9269	2.8329	0	0	3174	939.7721
16	5	2	2004	1	57	0	552.6389	483.7653	4.6	6.8799	3.43995	1	2.0235	3535	867.603
17	6	1	2002	1	52	9	0	179.6159	4.4	1.01175	0.4047	0	0	2959	1191.272
18	6	2	2004	1	54	9	552.6389	282.609	5.2	1.01175	1.01175	0	0	3396	755.6125
19	6	3	2006	1	56	9	73.68518	493.9735	6.3	1.6188	0.8094	1	0.8094	4130	1273.519
20	6	4	2008	1	58	9	368.4259	537.0602	5	1.82115	0.8094	1	0.8094	3562	890.9577
21	7	1	2002	1	40	9	0	333.2967	4.6	4.8564	1.2141	0	0	2904	724.5617
22	7	2	2004	1	42	9	276.3194	351.7781	4.3	8.094	4.047	1	2.0235	3550	1294.569
23	7	3	2006	1	44	9	828.9583	762.6265	4.2	6.0705	3.2376	1	2.0235	3445	715.8317
24	7	4	2008	1	46	9	1547.389	753.4219	4	6.0705	3.2376	1	3.2376	3541	791.1795
25	8	1	2002	1	56	2	340.7939	139.3165	6.2	2.0235	0.4047	0	0	3404	568.1288
26	8	2	2004	1	58	2	921.0648	378.6873	6.6	2.63055	0.8094	1	0.8094	3330	976.1981
27	8	3	2006	1	60	2	6447.453	205.5218	11.1	2.63055	1.2141	1	1.2141	2262	881.6929
28	8	4	2008	1	62	2	1326.333	359.2478	8.7	3.2376	2.4282	1	2.4282	3702	544.2683
29	9	1	2002	1	45	15	0	193.5166	6.3	16.9974	2.0235	0	0	1766	105.5554
30	9	2	2004	1	47	15	552.6389	357.4027	6.1	2.4282	0.8094	1	0.8094	2608	538.0217
31	9	3	2006	1	49	15	3684.259	532.2689	7.5	16.188	4.047	1	4.047	2946	601.5085
32	9	4	2008	1	51	15	552.6389	301.4039	8.8	4.8564	1.01175	1	1.01175	3956	296.8947
33	10	1	2002	1	28	3	5526.389	569.7169	4.2	7.2846	2.4282	0	0	3912	256.333
34	10	2	2004	1	30	3	1750.023	282.1783	9.7	8.094	2.8329	1	1.6188	3440	265.8239
35	10	3	2006	1	32	3	921.0648	544.6368	11.8	8.094	3.6423	1	3.6423	3322	810.1715
36	10	4	2008	1	34	3	884.2222	407.997	9.1	6.0705	4.047	1	4.047	3627	363.3024
37	11	1	2002	1	41	12	0	334.8731	5.8	10.5222	2.67102	1	0.52611	4659	708.6272
38	11	2	2004	1	43	12	0	292.8604	5	3.6423	2.0235	1	1.6188	2629	464.4841
39	11	3	2006	1	47	12	0	323.1071	5.9	3.2376	1.6188	1	1.6188	3409	672.9116
40	12	1	2002	1	47	15	552.6389	920.7427	3.6	20.235	1.2141	1	1.2141	3580	417.7701
41	12	2	2004	1	49	15	921.0648	1802.122	5.2	12.141	3.2376	1	4.047	3765	1401.086
42	12	3	2006	1	51	15	552.6389	1663.481	3.3	16.188	4.047	1	4.047	3235	347.4459
43	12	4	2008	1	53	15	0	781.9704	4.4	4.8564	2.0235	1	2.0235	2324	845.5732

44	13	1	2002	1	36	10	0	223.4551	4.3	1.6188	0.8094	1	0.4047	4661	628.8885
45	13	2	2004	1	38	10	460.5324	493.8791	4.4	1.6188	1.2141	1	0.4047	2743	356.9429
46	13	3	2006	1	40	10	0	354.801	4.1	2.0235	1.2141	1	1.2141	2770	545.8897
47	13	4	2008	1	42	10	276.3194	583.3709	2.5	1.6188	1.2141	1	1.2141	3120	840.7157
48	14	1	2002	1	50	0	1547.389	123.9245	4	1.2141	0.60705	0	0	2951	278.4496
49	14	2	2004	1	52	0	2763.194	155.5859	5.4	2.0235	0.4047	1	0.4047	3117	422.4537
50	14	3	2006	1	54	0	709.2198	132.8199	6.4	2.0235	0.4047	1	0.4047	2663	225.9909
51	14	4	2008	1	56	0	33.15833	84.67034	6	1.6188	0.60705	1	0.60705	1646	166.9197
52	15	1	2002	1	50	7	0	423.7177	4	6.0705	1.82115	0	0	3070	593.7509
53	15	2	2004	1	52	7	2210.555	446.9486	3.8	3.2376	1.6188	1	1.6188	2910	589.9684
54	16	1	2002	1	70	4	663.1666	279.669	11.4	4.8564	0.20235	0	0	3287	264.9603
55	16	2	2004	1	72	4	0	200.7503	1.8	0.4047	0.20235	0	0	3087	409.035
56	16	3	2006	1	74	4	128.9491	151.6828	2.3	0.8094	0.303525	1	0.303525	3357	282.3524
57	16	4	2008	1	76	4	265.2667	259.4886	1.6	0.8094	0.4047	1	0.4047	2779	335.2701
58	17	1	2002	1	23	15	0	401.4772	2.8	6.0705	2.8329	0	0	2559	187.2006
59	17	2	2004	1	25	15	0	1003.35	2.8	4.8564	2.0235	1	1.6188	4169	1279.27
60	17	3	2006	1	27	15	0	424.376	5.1	7.2846	2.0235	1	2.0235	2455	418.0751
61	17	4	2008	1	29	15	0	408.2427	4	4.047	2.0235	1	2.0235	3610	658.6608
62	18	1	2002	1	30	10	6631.666	126.549	3.6	1.2141	0.4047	0	0	2877	1051.89
63	18	2	2004	1	32	10	921.0648	188.8889	4.4	1.2141	0.4047	1	0.4047	3153	760.9465
64	18	3	2006	1	34	10	921.0648	248.6658	4.5	0.4047	0.4047	1	0.4047	3629	909.1193
65	18	4	2008	1	36	10	27.63194	134.8208	5	0.8094	0.4047	1	0.4047	1722	276.8568
66	19	1	2002	1	22	16	21221.33	459.501	3.9	1.2141	1.01175	1	0.60705	4084	682.3315
67	19	2	2004	1	24	16	1105.278	960.2417	5.4	1.2141	1.01175	1	0.4047	2978	705.426
68	19	3	2006	1	26	16	921.0648	329.0083	5.8	2.0235	1.01175	1	1.01175	3755	490.247
69	19	4	2008	1	28	16	1768.444	414.1188	4	1.82115	1.2141	1	1.2141	4008	652.4918
70	20	1	2002	1	25	11	0	303.57	3.6	3.03525	1.2141	1	0.60705	4181	1476.355
71	20	2	2004	1	27	11	0	234.1822	4.6	2.8329	2.8329	0	0	3159	614.5212
72	20	4	2008	1	31	11	0	672.6665	2.8	2.8329	2.8329	1	2.8329	3226	510.5073
73	21	1	2002	1	32	10	221.0555	164.7599	8	28.329	4.4517	0	0	2716	517.1315
74	21	2	2004	1	34	10	0	312.8695	7.5	30.3525	6.4752	0	0	3540	872.0659
75	21	3	2006	1	36	10	0	467.776	5.2	28.329	12.141	1	12.141	3551	645.6617
76	21	4	2008	1	38	10	736.8518	590.3735	4.8	8.094	2.8329	1	2.8329	3269	1071.751
77	22	1	2002	1	50	10	276.3194	359.048	2.5	4.047	4.047	1	0.8094	3811	760.6187
78	22	2	2004	1	52	10	442.1111	266.0755	2.5	4.047	2.8329	0	0	3842	845.5928
79	22	3	2006	1	54	10	1842.13	872.1086	2.6	4.047	3.2376	1	3.2376	3379	398.6263
80	22	4	2008	1	56	10	46.05324	734.2692	2.4	4.047	3.2376	1	3.2376	4037	886.8633
81	23	1	2002	1	22	10	0	135.9356	6.6	8.094	2.8329	0	0	3026	465.9055
82	23	2	2004	1	24	10	0	147.8854	8.5	7.2846	5.2611	1	0.8094	3675	422.0829
83	23	3	2006	1	26	10	3094.778	468.4687	9.3	6.0705	3.2376	1	3.2376	3522	515.3682
84	23	4	2008	1	28	10	2726.352	437.9945	7.9	8.9034	6.0705	1	6.0705	3030	673.4393
85	24	1	2002	1	70	7	0	129.9672	4.9	1.82115	0.8094	0	0	3321	517.4642
86	24	2	2004	1	72	7	276.3194	673.8699	4.5	3.2376	1.6188	1	0.4047	3927	975.3521
87	24	3	2006	1	74	7	460.5324	175.669	6.2	1.6188	1.2141	1	1.2141	1731	212.8633

88	24	4	2008	1	76	7	736.8518	265.0623	5.2	1.01175	1.01175	1	1.01175	1938	584.3647
89	25	1	2002	1	55	10	0	299.9261	4.6	5.6658	0.60705	0	0	3323	784.7546
90	25	2	2004	1	57	10	0	548.2492	5.4	6.4752	2.4282	1	0.4047	4621	605.092
91	25	3	2006	1	59	10	414.4791	140.0495	6.2	5.2611	1.2141	1	1.2141	4577	338.5955
92	25	4	2008	1	61	10	0	168.0672	8	4.8564	2.0235	1	2.0235	4108	275.6638
93	26	1	2002	1	37	17	884.2222	281.9638	5.5	8.094	1.82115	0	0	3031	533.6091
94	26	2	2004	1	39	17	368.4259	231.3259	5.5	8.9034	2.4282	1	1.6188	4617	401.7265
95	26	3	2006	1	41	17	1547.389	513.6646	4.6	8.094	1.6188	1	1.6188	2484	588.5679
96	26	4	2008	1	43	17	2763.194	1635.523	3.2	8.094	3.2376	1	3.2376	4476	689.6288
97	27	1	2002	1	50	0	0	387.3615	2.8	1.6188	0.4047	0	0	3162	344.4749
98	27	2	2004	1	52	0	110.5278	301.8039	2.6	1.517625	1.2141	1	0.4047	4050	661.539
99	27	3	2006	1	54	0	221.0555	165.0011	7.9	1.517625	1.2141	1	1.2141	3206	377.1163
100	27	4	2008	1	56	0	0	245.7057	4	1.6188	0.8094	1	0.8094	3627	584.6075
101	28	1	2002	1	32	9	0	166.2372	3.5	2.0235	1.6188	0	0	2984	438.4136
102	28	2	2004	1	34	9	0	168.2947	3.8	3.2376	1.6188	1	0.8094	3801	692.3235
103	28	3	2006	1	36	9	0	254.9629	3.8	3.2376	1.2141	1	1.2141	3834	634.254
104	28	4	2008	1	38	9	0	336.5684	5.8	4.047	1.6188	1	1.6188	3732	618.8131
105	29	1	2002	1	60	12	460.5324	314.4522	4.6	3.2376	1.2141	1	0.8094	3054	355.7473
106	29	2	2004	1	62	12	460.5324	546.6666	4.6	4.8564	2.4282	1	2.0235	3111	401.9961
107	29	3	2006	1	64	12	460.5324	574.5446	5.4	6.0705	1.2141	1	1.2141	4288	429.3907
108	29	4	2008	1	66	12	736.8518	309.8744	6.4	5.05875	2.8329	1	2.8329	3444	664.944
109	30	1	2002	1	50	4	147.3704	99.58966	4.7	2.0235	1.2141	0	0	3529	231.9121
110	30	2	2004	1	52	4	55.26389	104.2753	5.6	1.6188	1.2141	1	0.8094	3447	127.401
111	30	3	2006	1	54	4	0	85.15104	4.7	1.6188	0.60705	1	0.60705	3681	112.5813
112	30	4	2008	1	56	4	552.6389	174.6637	4.9	1.6188	0.8094	1	0.8094	4531	174.8714
113	31	1	2002	1	40	0	257.8981	164.3551	7.1	6.8799	1.2141	0	0	4017	570.6443
114	31	2	2004	1	42	0	0	242.2212	7.6	7.6893	2.4282	1	1.2141	4144	293.87
115	31	3	2006	1	44	0	0	129.34	7	5.2611	1.2141	1	1.2141	4281	104.16
116	31	4	2008	1	46	0	0	522.7595	7.1	4.8564	2.0235	1	2.0235	2100	425.0949
117	32	1	2002	1	32	17	169.4759	186.8445	2	1.2141	0.60705	1	0.60705	3165	443.4607
118	32	2	2004	1	34	17	1013.171	247.0941	3	1.2141	0.8094	1	0.4047	3783	1049.76
119	32	3	2006	1	36	17	2763.194	450.326	2.6	1.2141	1.2141	1	1.2141	3486	690.7318
120	32	4	2008	1	38	17	2394.768	548.0822	2.9	1.2141	0.60705	1	0.60705	3144	594.1539
121	33	1	2002	1	51	10	921.0648	250.409	6.6	14.1645	1.6188	1	1.6188	2777	676.7809
122	33	2	2004	1	53	10	1473.704	203.2742	6.2	10.1175	4.047	1	1.2141	2872	442.5669
123	33	3	2006	1	55	10	3684.259	756.6539	7.1	10.1175	2.4282	1	2.4282	3284	500.0204
124	33	4	2008	1	57	10	2505.296	783.0283	10	12.141	2.8329	1	2.8329	2741	401.0183
125	34	1	2002	1	45	10	0	188.1001	3.5	4.047	1.6188	1	0.60705	3935	504.2025
126	34	2	2004	1	47	10	0	475.8375	2.8	4.047	1.41645	1	0.8094	3376	949.0219
127	34	3	2006	1	49	10	0	421.8574	2.6	3.2376	1.6188	1	1.6188	3288	509.1817
128	34	4	2008	1	51	10	0	415.0414	4	3.03525	1.2141	1	1.2141	3655	767.6191
129	35	1	2002	1	35	10	0	269.1013	7.4	5.6658	1.6188	0	0	3596	800.0144
130	35	2	2004	1	37	10	0	199.7044	9.1	5.6658	2.8329	1	0.8094	2436	426.4025
131	35	3	2006	1	39	10	0	177.8637	8.1	1.6188	1.6188	1	1.6188	3169	111.3566

132	35	4	2008	1	41	10	0	374.189	4.7	4.047	1.6188	1	1.6188	2195	451.4575
133	36	1	2002	1	55	5	530.5333	365.2031	5.4	5.2611	2.0235	0	0	3570	898.3322
134	36	2	2004	1	57	5	405.2685	127.0451	5.9	3.2376	1.2141	0	0	2216	113.1425
135	36	3	2006	1	59	5	0	215.3542	7.4	4.047	0.8094	1	0.8094	3172	477.4929
136	36	4	2008	1	61	5	0	224.7347	7.1	4.8564	2.4282	1	2.4282	3112	443.3625
137	37	1	2002	1	43	10	0	185.8491	6	2.4282	1.2141	1	0.4047	3168	391.205
138	37	2	2004	1	45	10	0	523.1832	4.2	18.2115	12.141	1	8.094	3096	574.4424
139	37	3	2006	1	47	10	0	557.5744	4.9	19.4256	12.141	1	12.141	3091	726.2582
140	38	1	2002	1	20	16	663.1666	128.6418	6	13.15275	6.0705	0	0	2863	399.5347
141	38	2	2004	1	22	16	0	283.8238	5.8	10.1175	5.6658	1	0.8094	3217	469.5837
142	38	3	2006	1	24	16	0	209.2946	7.1	14.1645	5.6658	1	5.6658	2632	425.2374
143	38	4	2008	1	26	16	2578.981	258.3455	9.6	3.03525	2.4282	1	2.4282	4266	456.9387
144	39	1	2002	1	28	15	254.2139	136.534	7.5	10.1175	4.8564	0	0	2068	260.8612
145	39	2	2004	1	30	15	2800.037	254.9795	8.1	10.1175	4.8564	1	0.8094	3364	416.2455
146	39	3	2006	1	32	15	0	412.5628	4.4	6.4752	6.4752	1	6.4752	4537	749.735
147	39	4	2008	1	34	15	2947.407	350.6042	9.5	10.1175	7.6893	1	7.6893	4168	456.7757
148	40	1	2002	1	55	9	368.4259	129.5582	6.8	1.6188	1.6188	0	0	3488	551.4751
149	40	2	2004	1	57	9	1363.176	176.1664	7.4	1.41645	1.41645	0	0	2778	244.09
150	40	3	2006	1	59	9	552.6389	355.2957	6.3	1.6188	0.8094	1	0.8094	1872	295.7789
151	40	4	2008	1	61	9	368.4259	356.3801	8.7	1.2141	3.6423	1	3.6423	2159	395.6286
152	41	1	2002	1	25	12	0	140.7879	5.4	2.22585	1.01175	0	0	2777	409.7037
153	41	2	2004	1	27	12	368.4259	191.5719	5	2.22585	1.2141	1	0.8094	2683	278.6049
154	41	3	2006	1	29	12	221.0555	208.2046	5.8	2.22585	1.6188	1	1.6188	4224	295.5499
155	41	4	2008	1	31	12	2652.667	242.2349	6.6	2.22585	0.60705	1	0.60705	2770	336.8645
156	42	1	2002	1	19	9	736.8518	206.7091	7.2	7.08225	2.0235	0	0	3905	2015.674
157	42	2	2004	1	21	9	663.1666	881.7758	3.4	3.03525	2.0235	1	0.4047	4090	1681.976
158	42	3	2006	1	23	9	331.5833	206.3638	9.5	2.8329	1.6188	1	1.6188	3280	387.4343
159	42	4	2008	1	25	9	0	307.9468	10.5	2.8329	2.0235	1	2.0235	3566	441.8304
160	43	1	2002	1	43	16	1105.278	337.1241	10	14.1645	6.0705	0	0	2577	560.4757
161	43	2	2004	1	45	16	552.6389	144.0503	4.8	6.0705	2.4282	1	0.8094	3377	1414.502
162	43	3	2006	1	47	16	0	331.2615	4.1	5.6658	1.6188	1	1.6188	3483	589.1633
163	43	4	2008	1	49	16	0	844.5851	3.4	8.094	2.8329	1	2.8329	3055	915.5962
164	44	1	2002	1	24	17	921.0648	284.9815	5	8.094	1.6188	1	0.4047	3478	657.6091
165	44	2	2004	1	26	17	644.7453	101.0402	6.1	4.047	4.047	1	1.2141	4394	363.7469
166	44	3	2006	1	28	17	1565.81	544.6443	5	4.4517	2.0235	1	2.0235	2865	654.3487
167	44	4	2008	1	30	17	5526.389	1480.666	5.8	6.0705	4.047	1	4.047	4397	932.584
168	45	1	2002	1	38	10	460.5324	288.0591	4.3	8.094	3.2376	1	0.4047	3413	496.6462
169	45	2	2004	1	40	10	1289.491	606.2794	5.3	14.1645	8.094	1	3.2376	3325	725.3484
170	45	3	2006	1	42	10	552.6389	726.863	4.2	8.094	2.8329	1	2.8329	4212	631.4895
171	45	4	2008	1	44	10	921.0648	497.7975	5	11.3316	6.0705	1	6.0705	2591	530.2883
172	46	1	2002	1	68	10	0	158.1335	8.2	1.6188	0.4047	1	0.4047	3219	262.1399
173	46	2	2004	1	70	10	552.6389	214.2337	5.2	8.9034	6.4752	1	2.0235	3020	644.0822
174	46	3	2006	1	72	10	0	813.1505	10.2	18.2115	3.2376	1	3.2376	4295	1045.161
175	46	4	2008	1	74	10	0	621.9604	1.8	3.2376	0.8094	1	0.8094	4213	1531.072

176	47	1	2002	1	45	17	0	243.4346	3.4	2.0235	1.2141	0	0	3160	612.8273
177	47	3	2006	1	49	17	1547.389	487.9199	4.4	1.6188	1.2141	1	1.2141	3367	337.808
178	47	4	2008	1	51	17	2026.342	588.2377	4.2	2.0235	1.2141	1	1.2141	2974	537.622
179	48	1	2002	1	45	8	294.7407	199.234	9.5	1.2141	0.8094	0	0	2359	647.8131
180	48	2	2004	1	47	8	221.0555	167.3772	10.2	3.6423	0.8094	1	0.8094	2996	360.739
181	48	3	2006	1	49	8	110.5278	394.0994	4.4	4.8564	1.2141	1	1.2141	4141	249.3018
182	48	4	2008	1	51	8	0	366.909	4.7	1.41645	3.2376	1	3.2376	3311	368.9753
183	49	1	2002	1	65	0	184.213	188.5925	3.5	0.8094	0.4047	0	0	2320	332.5311
184	49	2	2004	1	67	0	0	198.5107	3.8	7.2846	5.2611	1	2.0235	4568	2645.247
185	49	3	2006	1	69	0	0	451.5404	4.6	2.0235	1.2141	1	1.2141	3876	335.2433
186	49	4	2008	1	71	0	0	587.8057	5.8	2.0235	4.047	1	4.047	3884	496.6872
187	50	1	2002	1	22	15	0	284.2429	3.6	2.0235	2.0235	0	0	3916	1301.705
188	50	2	2004	1	24	15	0	316.3529	4.6	3.03525	2.4282	1	0.4047	3749	1556.849
189	50	3	2006	1	26	15	331.5833	222.2847	3.4	2.4282	1.2141	1	1.2141	4267	148.5559
190	50	4	2008	1	28	15	442.1111	215.1366	4.3	2.4282	1.6188	1	1.6188	2346	361.2683
191	51	1	2002	1	23	12	368.4259	363.5618	2.6	2.8329	1.6188	0	0	3331	1188.078
192	51	2	2004	1	25	12	442.1111	313.5289	3.4	3.2376	2.4282	1	0.4047	3727	439.1841
193	51	3	2006	1	27	12	1215.805	297.5849	4.2	2.8329	0.4047	1	0.4047	3175	390.5862
194	51	4	2008	1	29	12	2210.555	262.3715	4.8	2.8329	2.0235	1	2.0235	2987	572.2297
195	52	1	2002	1	58	10	921.0648	284.2183	4.2	2.0235	0.4047	0	0	2192	558.8895
196	52	2	2004	1	60	10	884.2222	893.1202	6.1	4.8564	1.6188	1	0.8094	2768	500.7025
197	52	3	2006	1	62	10	828.9583	366.5842	3.8	5.2611	1.01175	1	1.01175	4531	283.0512
198	52	4	2008	1	64	10	1547.389	370.8532	4.4	3.6423	1.6188	1	1.6188	2573	394.2296
199	53	1	2002	1	42	15	442.1111	87.70346	7.1	3.6423	2.4282	0	0	2899	285.8745
200	53	2	2004	1	44	15	552.6389	179.7787	4.9	0	1.6188	1	0.8094	2647	372.4171
201	53	3	2006	1	46	15	921.0648	442.7074	4.1	1.41645	1.2141	1	1.2141	3005	486.1153
202	53	4	2008	1	48	15	921.0648	300.727	4	2.0235	2.0235	1	2.0235	2353	306.5311
203	54	1	2002	1	40	0	0	296.6762	3.3	2.8329	1.6188	0	0	3166	877.6861
204	54	2	2004	1	42	0	294.7407	102.0691	4.4	2.8329	2.4282	0	0	2870	232.1806
205	54	3	2006	1	44	0	257.8981	118.6568	3.9	0.8094	2.0235	1	2.0235	2566	497.8049
206	54	4	2008	1	46	0	644.7453	166.4792	4.3	0.8094	2.4282	1	2.4282	2910	731.0275
207	55	1	2002	1	59	7	92.10648	131.5278	7.3	5.6658	1.41645	0	0	2956	199.1052
208	55	2	2004	1	61	7	368.4259	584.6635	5.6	8.094	6.0705	1	4.047	3838	357.7881
209	55	3	2006	1	63	7	552.6389	203.0852	4	1.41645	1.2141	1	1.2141	2573	364.7715
210	55	4	2008	1	65	7	221.0555	344.728	9.5	3.03525	2.63055	1	2.63055	3339	480.406
211	56	1	2002	1	33	10	0	175.5437	6.3	18.2115	8.4987	0	0	2607	438.9639
212	56	2	2004	1	35	10	0	366.6326	6.2	14.1645	8.9034	1	8.094	2808	410.8836
213	56	3	2006	1	37	10	736.8518	276.7784	4.9	14.1645	8.094	1	8.094	2060	603.7515
214	57	1	2002	1	52	9	46.05324	207.1194	3.4	1.74021	0.8094	0	0	3871	440.2271
215	57	2	2004	1	54	9	1289.491	276.7985	4.2	1.82115	1.2141	1	0.8094	2593	425.0201
216	57	3	2006	1	56	9	0	197.2926	4.4	1.922325	1.2141	1	1.2141	2990	304.4538
217	57	4	2008	1	58	9	1105.278	235.6122	5.2	2.0235	1.2141	1	1.2141	2169	346.046
218	58	1	2002	1	42	10	184.213	247.0485	6	5.46345	2.4282	1	1.6188	4188	1323.091
219	58	2	2004	1	44	10	921.0648	972.4966	4.4	8.094	6.0705	1	3.6423	3723	794.4633

220	58	3	2006	1	46	10	736.8518	786.7101	3.4	4.047	4.047	1	4.047	3865	1435.681
221	58	4	2008	1	48	10	1473.704	918.0079	2.4	8.094	6.0705	1	6.0705	3373	752.6675
222	59	1	2002	1	30	4	73.68518	259.4994	4	1.6188	0.60705	0	0	3606	899.693
223	59	2	2004	1	32	4	994.7499	163.0438	6.8	1.517625	1.517625	1	0.4047	2706	510.2245
224	59	3	2006	1	34	4	921.0648	154.377	5.2	1.82115	1.2141	1	1.2141	3487	583.9396
225	59	4	2008	1	36	4	663.1666	190.352	5.6	1.2141	1.2141	1	1.2141	3685	736.2771
226	60	1	2002	1	42	10	0	226.0156	7.7	4.4517	1.01175	1	1.01175	2969	365.6494
227	60	2	2004	1	44	10	3168.463	342.7012	3.9	7.08225	7.08225	1	3.2376	3160	1085.847
228	60	3	2006	1	46	10	184.213	394.4232	4.1	3.2376	2.4282	1	2.4282	2748	425.5022
229	60	4	2008	1	48	10	1105.278	289.0349	4.1	3.03525	3.2376	1	3.2376	2118	503.5422
230	61	1	2002	1	31	15	1455.282	293.12	11.8	2.0235	1.6188	1	0.4047	3630	625.7908
231	61	2	2004	1	33	15	2210.555	276.0735	5	12.141	6.0705	1	3.6423	4025	307.7644
232	61	3	2006	1	35	15	1105.278	418.4094	4.3	2.4282	1.6188	1	1.6188	3750	242.1138
233	61	4	2008	1	37	15	3684.259	434.2141	3.6	2.0235	2.0235	1	2.0235	4191	666.5233
234	62	1	2002	1	29	17	73.68518	508.0181	11	16.188	1.6188	1	1.2141	3338	855.3644
235	62	2	2004	1	31	17	0	1030.503	13.5	16.188	8.9034	1	4.8564	3269	850.9374
236	62	3	2006	1	33	17	0	677.2356	4.9	5.6658	4.4517	1	4.4517	4682	975.8411
237	62	4	2008	1	35	17	0	266.2121	8.6	10.1175	8.094	1	8.094	3631	694.9001
238	63	1	2002	1	43	15	0	413.1617	9	14.1645	4.8564	0	0	3325	655.4544
239	63	2	2004	1	45	15	0	470.4093	4.5	16.188	12.141	1	8.094	3650	826.7643
240	64	1	2002	1	30	7	0	240.0492	3.6	4.047	3.2376	0	0	3252	800.8058
241	64	2	2004	1	32	7	0	424.2231	2.6	2.8329	2.4282	1	2.0235	3284	962.6039
242	64	3	2006	1	34	7	0	404.0927	2.6	3.2376	2.8329	1	2.8329	3235	952.0292
243	64	4	2008	1	36	7	0	459.99	3	3.6423	3.6423	1	3.6423	3069	779.3839
244	65	1	2002	1	30	9	0	225.0718	5.4	2.4282	1.6188	0	0	3156	861.0677
245	65	2	2004	1	32	9	1013.171	295.1887	9.8	2.0235	1.6188	1	0.8094	3821	590.7439
246	65	3	2006	1	34	9	0	202.4222	5.7	2.0235	2.0235	1	2.0235	3111	409.9191
247	65	4	2008	1	36	9	921.0648	413.9483	3.7	2.0235	2.0235	1	2.0235	2820	565.6309
248	66	1	2002	1	60	0	0	76.07714	7.9	3.6423	2.4282	0	0	2210	125.3527
249	66	2	2004	1	62	0	1289.491	169.5362	8.2	4.4517	3.2376	1	0.4047	3820	272.0681
250	66	3	2006	1	64	0	0	167.1303	5.3	4.047	3.2376	1	3.2376	2927	440.2029
251	66	4	2008	1	66	0	0	264.1848	4.9	2.4282	2.0235	1	2.0235	3035	474.5562
252	67	1	2002	1	60	0	0	350.2503	4.5	12.141	5.2611	0	0	3346	660.6846
253	67	2	2004	1	62	0	0	362.0191	4.5	12.141	8.9034	1	2.0235	3095	636.9187
254	67	3	2006	1	64	0	0	593.9734	4.6	14.1645	9.7128	0	0	2791	94.83667
255	67	4	2008	1	66	0	0	469.7217	4	8.094	6.0705	1	6.0705	2375	551.346
256	68	1	2002	1	45	17	46.05324	177.6611	9.2	8.094	2.0235	0	0	3403	344.1234
257	68	2	2004	1	47	17	0	509.5589	4.4	7.6893	5.2611	1	0.8094	2660	612.4717
258	69	1	2002	1	29	12	0	278.4161	7.4	8.094	4.047	0	0	3524	594.0279
259	69	2	2004	1	31	12	0	311.628	8.2	8.9034	4.8564	1	1.6188	4236	1577.126
260	69	3	2006	1	33	12	0	282.1727	6.7	8.094	4.047	1	4.047	3221	379.8043
261	69	4	2008	1	35	12	0	2019.784	8.9	8.094	4.4517	1	4.4517	2492	406.8964
262	70	1	2002	1	45	11	0	297.4559	9.9	12.141	4.8564	1	0.8094	2710	336.712
263	70	2	2004	1	47	11	0	269.4588	4.6	11.3316	3.2376	1	3.2376	3184	500.0291

264	70	3	2006	1	49	11	0	261.4475	5.8	10.5222	4.8564	1	4.8564	3410	358.4704
265	70	4	2008	1	51	11	0	244.7042	5.8	6.0705	2.8329	1	2.8329	2172	392.3181
266	71	1	2002	1	30	15	1370.544	159.5404	5	14.1645	3.2376	1	1.2141	3452	382.1028
267	71	2	2004	1	32	15	2763.194	341.5189	4.5	14.1645	4.8564	1	2.4282	3481	435.5024
268	71	3	2006	1	34	15	1547.389	642.6805	3.2	14.1645	4.4517	1	4.4517	2900	541.9623
269	71	4	2008	1	36	15	1768.444	809.827	5.7	12.5457	2.0235	1	2.0235	2015	367.6416
270	72	1	2002	1	40	7	0	279.1957	5.1	5.6658	2.0235	1	0.8094	4077	496.3526
271	72	2	2004	1	42	7	0	646.8577	10.9	40.47	16.188	1	8.094	3131	464.1034
272	72	3	2006	1	44	7	921.0648	634.5851	5.6	40.47	12.9504	1	12.9504	3559	669.8954
273	72	4	2008	1	46	7	1473.704	4170.619	11.8	40.47	16.188	1	16.188	3958	692.6591
274	73	1	2002	1	42	4	0	211.1941	4.3	4.047	2.4282	0	0	3739	477.1339
275	73	2	2004	1	44	4	0	268.8495	4	5.6658	2.8329	1	1.6188	4303	562.6519
276	73	3	2006	1	46	4	0	270.6479	5.2	3.84465	1.2141	1	1.2141	2988	354.2663
277	73	4	2008	1	48	4	0	284.0687	5.4	6.0705	3.2376	1	3.2376	2222	397.7102
278	74	1	2002	1	25	12	589.4814	383.3402	2.6	7.2846	3.6423	0	0	3124	290.1554
279	74	2	2004	1	27	12	128.9491	202.7854	3.4	7.2846	4.8564	1	2.8329	3526	454.7739
280	74	3	2006	1	29	12	184.213	405.7792	3.6	7.2846	2.4282	1	2.4282	3844	348.5547
281	74	4	2008	1	31	12	1105.278	367.097	3.4	7.2846	4.4517	1	4.4517	3606	529.1204
282	75	1	2002	1	60	0	0	256.0525	6.6	12.141	3.6423	0	0	3467	567.884
283	75	2	2004	1	62	0	0	626.6769	6.6	12.141	7.6893	1	7.6893	3937	603.4697
284	75	3	2006	1	64	0	0	498.6476	6.3	12.141	1.2141	1	1.2141	2309	328.2644
285	75	4	2008	1	66	0	0	964.8333	9.5	12.141	6.4752	1	6.4752	3557	467.0521
286	76	1	2002	1	45	12	0	381.8629	4.7	4.047	3.2376	0	0	3298	1143.339
287	76	2	2004	1	47	12	0	272.8286	9.5	26.3055	12.141	0	0	2302	418.356
288	76	3	2006	1	49	12	0	562.3027	4.2	16.188	8.094	1	8.094	3644	690.7344
289	76	4	2008	1	51	12	0	415.4425	5.4	16.9974	10.1175	1	10.1175	2131	411.9889
290	77	1	2002	1	28	8	0	244.1526	4.6	3.2376	0.8094	0	0	3268	545.9361
291	77	2	2004	1	30	8	0	135.7825	5.2	2.8329	1.2141	1	0.4047	3899	450.6959
292	77	3	2006	1	32	8	442.1111	138.6488	5.3	3.2376	1.2141	1	1.2141	2260	270.2457
293	77	4	2008	1	34	8	460.5324	161.7341	5.4	3.6423	1.6188	1	1.6188	2204	252.767
294	78	1	2002	1	48	10	0	360.5663	3.8	2.8329	1.6188	0	0	3188	1031.037
295	78	2	2004	1	50	10	0	291.3415	4.1	8.094	4.8564	1	0.4047	3434	412.0567
296	78	3	2006	1	52	10	0	348.7894	5	2.8329	2.0235	1	2.0235	4105	802.2074
297	78	4	2008	1	54	10	0	367.8449	5	4.8564	2.0235	1	2.0235	2158	457.7156
298	79	1	2002	1	30	7	265.2667	209.4144	5.8	4.047	2.4282	0	0	2259	447.3935
299	79	2	2004	1	32	7	313.162	210.3866	8.9	4.047	2.8329	1	2.0235	2308	705.5757
300	79	3	2006	1	34	7	0	238.9091	5.8	4.24935	2.0235	1	2.0235	2873	445.5728
301	79	4	2008	1	36	7	0	479.3307	3.6	4.047	2.8329	1	2.8329	2610	476.7461
302	80	1	2002	1	40	7	368.4259	237.9975	5.4	6.0705	2.0235	1	0.44517	2719	360.4825
303	80	2	2004	1	42	7	0	379.0424	6.2	10.1175	4.047	1	2.8329	4289	299.3854
304	80	3	2006	1	44	7	0	260.8875	5.2	10.1175	6.0705	1	6.0705	2846	364.9867
305	80	4	2008	1	46	7	0	246.5781	12.5	10.1175	2.4282	1	2.4282	1110	272.5241
306	81	1	2002	1	38	10	0	245.158	3.8	4.047	3.2376	1	0.8094	4311	490.1255
307	81	2	2004	1	40	10	0	304.1564	4.6	5.6658	2.0235	1	2.0235	3384	690.0536

308	81	3	2006	1	42	10	0	196.4396	4.6	5.6658	2.8329	1	2.8329	3186	283.3104
309	81	4	2008	1	44	10	0	314.0708	5.8	6.4752	3.6423	1	3.6423	2848	542.6078
310	82	1	2002	1	38	12	0	234.5343	3.5	2.8329	0.8094	1	0.8094	4181	838.4753
311	82	2	2004	1	40	12	1842.13	683.4243	18.3	40.47	12.141	1	4.047	3962	1539.539
312	82	3	2006	1	42	12	184.213	549.0443	5.8	9.7128	0.505875	1	0.505875	4610	1296.695
313	82	4	2008	1	44	12	1105.278	701.343	5.7	10.1175	5.6658	1	5.6658	2047	721.8152
314	83	1	2002	1	27	9	221.0555	170.0041	8.2	24.282	6.0705	0	0	3404	851.7573
315	83	2	2004	1	29	9	221.0555	511.4598	9.3	24.282	8.094	1	1.6188	3392	793.4652
316	83	3	2006	1	31	9	0	640.2518	10.7	16.188	6.0705	1	6.0705	4446	1009.728
317	83	4	2008	1	33	9	442.1111	768.9145	9.6	24.282	9.3081	1	9.3081	3751	611.2621
318	84	1	2002	1	40	0	0	255.714	11.7	8.094	4.047	1	1.2141	3988	318.5154
319	84	2	2004	1	42	0	0	757.5717	12.3	24.282	16.188	1	8.094	3963	505.9897
320	84	4	2008	1	46	0	2763.194	651.2127	6.9	10.9269	10.1175	1	10.1175	3597	484.4374
321	85	1	2002	1	25	9	0	233.0016	9.2	6.0705	2.8329	1	0.8094	3629	354.7832
322	85	2	2004	1	27	9	0	400.3956	9.5	8.9034	4.8564	1	0.8094	4032	1635.769
323	85	3	2006	1	29	9	0	352.5613	10.6	7.6893	6.0705	1	6.0705	3686	429.4224
324	85	4	2008	1	31	9	0	476.6107	9.3	10.1175	5.6658	1	5.6658	3935	377.2048
325	86	1	2002	1	42	15	460.5324	227.9852	4.2	4.047	1.2141	0	0	3734	692.6291
326	86	2	2004	1	44	15	1105.278	257.851	5.8	2.0235	0.8094	0	0	2205	634.6221
327	86	3	2006	1	46	15	0	263.9382	7.8	2.4282	0.60705	1	0.60705	2387	653.3749
328	86	4	2008	1	48	15	1657.917	537.5884	4.2	2.0235	0.8094	1	0.8094	3549	757.7784
329	87	1	2002	1	31	10	442.1111	128.5187	3.3	1.6188	0.8094	0	0	3676	275.516
330	87	2	2004	1	32	10	1842.13	498.954	9.4	20.235	4.047	0	0	2829	316.823
331	88	1	2002	1	40	9	0	228.5597	4.4	2.4282	1.2141	0	0	2817	1070.165
332	88	2	2004	1	42	9	184.213	182.6869	5.5	2.4282	0	0	0	2416	349.2896
333	88	3	2006	1	44	9	0	422.682	4.7	2.4282	1.2141	1	1.2141	2469	472.4959
334	88	4	2008	1	46	9	0	566.2071	4.8	4.047	1.2141	1	1.2141	2454	733.9276
335	89	1	2002	1	46	15	921.0648	472.7123	4	16.188	6.0705	1	0.60705	3793	589.9889
336	89	2	2004	1	48	15	7000.092	633.874	10.6	40.47	12.141	1	6.0705	3993	508.1541
337	90	1	2002	1	48	10	0	181.1572	4.6	7.89165	2.0235	1	0.8094	2117	450.0011
338	90	2	2004	1	50	10	0	193.2632	5.8	6.0705	2.0235	0	0	2301	484.5652
339	90	4	2008	1	54	10	0	428.0522	6.4	3.2376	2.0235	1	2.0235	2586	556.8231
340	91	1	2002	1	55	7	0	112.3	7.3	10.9269	2.4282	1	0.8094	1986	285.2624
341	91	2	2004	1	57	7	1105.278	141.9009	9.2	7.2846	2.4282	0	0	2409	278.4254
342	91	4	2008	1	61	7	0	403.9609	11.5	13.3551	3.2376	1	3.2376	1794	254.4665
343	92	1	2002	1	45	7	552.6389	327.8621	5	0.8094	0.8094	0	0	3656	729.4124
344	92	2	2004	1	47	7	1105.278	267.9945	4.3	6.0705	1.2141	0	0	2912	517.9747
345	92	3	2006	1	49	7	1013.171	556.5325	3.3	4.8564	3.2376	1	3.2376	3542	961.6425
346	92	4	2008	1	51	7	1105.278	661.3186	3.2	2.8329	0.8094	1	0.8094	3128	807.5735
347	93	1	2002	1	35	10	0	278.1699	5.1	3.2376	0.8094	0	0	3727	1072.044
348	93	4	2008	1	41	10	0	315.0539	5	4.047	0.4047	1	0.4047	3744	361.7276
349	94	1	2002	1	52	15	663.1666	289.2901	4.9	4.047	1.01175	0	0	3500	724.0977
350	94	2	2004	1	54	15	1842.13	441.8632	5.9	3.2376	1.2141	0	0	3611	498.5352
351	94	4	2008	1	58	15	552.6389	243.7014	3.1	2.4282	0.4047	1	0.4047	2931	409.6515

352	95	1	2002	1	30	10	0	186.5613	7.3	8.094	1.6188	1	0.4047	3479	375.344
353	95	2	2004	1	32	10	0	186.9441	10.1	12.141	4.047	0	0	2692	420.5576
354	95	3	2006	1	34	10	0	379.8708	8.4	14.5692	3.2376	1	3.2376	4280	431.3232
355	95	4	2008	1	36	10	0	466.0817	10.4	14.5692	4.8564	1	4.8564	2267	438.4892
356	96	1	2002	1	30	12	0	338.0796	7.9	10.1175	4.047	1	0.4047	3185	563.5616
357	96	2	2004	1	32	12	0	374.5895	9.5	10.72455	2.8329	1	1.6188	3506	378.4905
358	96	3	2006	1	34	12	294.7407	150.3785	10.5	3.2376	1.6188	1	1.6188	2860	317.9543
359	96	4	2008	1	36	12	0	143.6764	6.2	3.6423	1.6188	1	1.6188	1738	214.7874
360	97	1	2002	1	40	10	0	222.5687	3.3	2.8329	1.41645	1	0.4047	3411	577.9337
361	97	2	2004	1	42	10	515.7963	271.7235	4.2	1.6188	0.4047	0	0	2622	296.23
362	98	1	2002	1	28	9	0	247.0485	8.3	3.2376	0.8094	1	0.4047	3504	516.6569
363	98	2	2004	1	30	9	276.3194	439.5816	10.2	3.6423	1.2141	0	0	2058	206.7646
364	98	3	2006	1	32	9	0	171.0254	7.9	2.8329	0.4047	1	0.4047	2504	248.9808
365	98	4	2008	1	34	9	736.8518	139.7744	7.3	2.4282	0.8094	1	0.8094	1844	388.4886
366	99	1	2002	1	50	10	0	208.2889	6.3	4.047	1.6188	1	0.4047	3221	391.5964
367	99	2	2004	1	52	10	0	179.3224	8.7	10.9269	4.8564	1	0.8094	2756	443.2432
368	99	3	2006	1	54	10	0	247.0249	8	12.141	1.82115	0	0	2215	401.4456
369	99	4	2008	1	56	10	0	275.2928	5.7	10.1175	2.8329	1	2.8329	2745	393.0712
370	100	1	2002	1	35	0	0	256.8527	2.8	2.0235	1.2141	1	0.4047	4119	919.0319
371	100	2	2004	1	37	0	0	195.7162	3.9	2.8329	0.4047	0	0	2883	695.8286
372	100	3	2006	1	39	0	147.3704	196.0063	4	2.0235	0.8094	1	0.8094	3264	685.1563
373	100	4	2008	1	41	0	0	381.3487	4	2.0235	0.4047	1	0.4047	2805	425.9799
374	101	1	2002	1	25	11	0	331.5388	4.4	2.0235	0.8094	1	0.4047	3974	1380.382
375	101	2	2004	1	27	11	442.1111	109.4004	5.7	3.6423	0.8094	0	0	2178	322.8651
376	101	3	2006	1	29	11	408.9528	108.4664	6.2	3.2376	0.4047	1	0.4047	3173	326.5031
377	101	4	2008	1	31	11	257.8981	204.323	6.7	3.2376	1.6188	1	1.6188	2442	366.3543
378	102	1	2002	1	28	10	0	191.4102	7.3	2.8329	0.4047	1	0.4047	4348	433.9814
379	102	2	2004	1	30	10	9210.647	168.9859	8	4.8564	2.0235	0	0	3212	292.9391
380	102	4	2008	1	34	10	1842.13	377.2224	8.5	8.094	1.6188	1	1.6188	2996	612.9132
381	103	1	2002	1	20	12	0	136.9635	7.9	8.094	1.41645	1	0.44517	3537	339.4457
382	103	2	2004	1	22	12	128.9491	287.658	9.5	9.3081	2.8329	1	0.4047	3562	471.0917
383	103	3	2006	1	24	12	1289.491	983.5394	10.5	8.4987	1.6188	1	1.6188	3638	726.251
384	103	4	2008	1	26	12	1842.13	315.0714	11.2	6.0705	1.6188	1	1.6188	3982	233.6126
385	104	1	2002	1	27	12	0	230.4473	6.9	6.0705	1.6188	1	0.4047	2911	739.9219
386	104	2	2004	1	29	12	0	292.2397	7.7	6.0705	2.0235	0	0	2465	445.3812
387	104	3	2006	1	31	12	221.0555	140.1169	9.2	5.6658	2.0235	1	2.0235	2044	329.9861
388	104	4	2008	1	33	12	1842.13	298.6964	7.2	5.6658	1.6188	1	1.6188	3333	551.7387
389	105	1	2002	1	35	5	276.3194	282.2979	3.7	0.60705	0.4047	1	0.4047	3780	563.4476
390	105	2	2004	1	37	5	442.1111	179.8727	4	1.2141	1.2141	0	0	2378	411.1741
391	106	1	2002	2	25	15	110.5278	125.5355	5.2	2.0235	0.8094	1	0.8094	2335	409.1841
392	106	2	2004	2	27	15	0	179.3827	6.4	6.0705	1.2141	1	1.2141	3142	267.4009
393	106	3	2006	2	29	15	0	292.9016	2.8	2.0235	2.0235	1	2.0235	2634	416.7113
394	106	4	2008	2	31	15	0	814.3645	2.8	2.0235	2.4282	1	2.4282	3411	363.2659
395	107	1	2002	2	37	12	0	259.4419	2.7	6.4752	2.0235	1	2.0235	3925	696.2402

396	107	2	2004	2	39	12	1326.333	141.7462	7	3.6423	0.8094	1	0.8094	3759	363.2503
397	107	3	2006	2	41	12	0	357.2746	3.5	3.6423	1.6188	1	1.6188	3656	813.2922
398	107	4	2008	2	43	12	0	265.9464	8.7	3.6423	3.2376	1	3.2376	3728	262.8823
399	108	1	2002	2	45	6	0	82.119	4.4	3.6423	0.8094	0	0	2041	599.6042
400	108	2	2004	2	47	6	0	150.9019	5.3	2.4282	0.8094	0	0	2713	444.5823
401	108	3	2006	2	49	6	0	408.0279	3.4	1.2141	0.60705	1	0.60705	3703	861.2695
402	108	4	2008	2	51	6	202.6342	296.95	2.6	1.2141	0.60705	1	0.60705	1853	123.4328
403	109	1	2002	2	35	10	0	109.5757	4.2	4.047	0.4047	0	0	3066	465.7242
404	109	2	2004	2	37	10	0	162.9529	4.4	4.047	0.8094	1	0.8094	2472	317.2173
405	109	3	2006	2	39	10	921.0648	283.7306	3.1	2.63055	1.82115	1	1.82115	3312	511.1265
406	109	4	2008	2	41	10	460.5324	455.2645	3.2	2.63055	1.41645	1	1.41645	3891	394.7852
407	110	1	2002	2	65	21	8842.222	223.8428	4.6	14.1645	4.047	0	0	3216	474.5989
408	110	2	2004	2	67	21	36842.59	1364.143	1.6	12.9504	4.047	0	0	4169	692.5336
409	111	1	2002	2	71	10	309.4778	383.5864	4.8	4.8564	0.8094	0	0	2416	208.2541
410	111	2	2004	2	73	10	0	157.3064	5.2	1.299087	0.4047	1	0.8094	2847	271.5904
411	111	3	2006	2	75	10	0	222.2532	4.1	1.2141	1.2141	1	1.2141	3987	410.0707
412	111	4	2008	2	77	10	1908.446	499.8647	9	5.2611	4.047	1	4.047	3855	342.5335
413	112	1	2002	2	40	10	1326.333	173.3131	4.2	0.8094	0.8094	0	0	2840	398.0371
414	112	2	2004	2	42	10	0	155.6263	11.9	2.0235	1.2141	0	0	2025	204.7538
415	112	3	2006	2	44	10	0	198.9506	3.9	2.0235	1.2141	1	1.2141	3141	487.1671
416	112	4	2008	2	46	10	73.68518	283.8305	4.3	1.2141	1.2141	1	1.2141	2371	431.1833
417	113	1	2002	2	65	0	397.9	108.5409	4.4	2.4282	0.4047	0	0	3134	350.4063
418	113	2	2004	2	67	0	0	124.3098	6.8	2.0235	1.6188	0	0	1798	218.9791
419	113	4	2008	2	71	0	184.213	305.0764	3.2	0.8094	0.8094	1	0.8094	3836	278.7665
420	114	1	2002	2	50	3	66.31667	110.8863	5.3	3.84465	1.01175	1	0.8094	3067	482.8471
421	114	2	2004	2	52	3	0	374.8847	6.5	4.913058	1.675458	1	1.6188	3661	276.3428
422	114	4	2008	2	56	3	36.84259	283.7303	4.2	4.8564	1.01175	1	1.01175	2831	612.5866
423	115	1	2002	2	29	2	176.8444	168.7115	3	2.63055	0.4047	1	0.4047	2894	474.4695
424	115	2	2004	2	31	2	0	185.6905	5.2	2.4282	0.8094	1	0.8094	3196	401.0296
425	115	3	2006	2	33	2	0	271.1941	4.2	2.8329	0.8094	1	0.8094	3639	1015.538
426	116	1	2002	2	75	0	0	203.7546	5.2	4.047	1.2141	1	0.4047	2904	450.4192
427	116	2	2004	2	77	0	0	153.3164	6.1	4.047	0.8094	1	0.8094	3032	434.1397
428	116	3	2006	2	79	0	672.3773	247.5891	6.3	2.4282	1.2141	1	1.2141	3028	623.0123
429	116	4	2008	2	81	0	184.213	258.6947	5.6	1.29504	0.8094	1	0.8094	3987	355.2166
430	117	1	2002	2	45	0	0	92.65654	4.2	2.4282	0.8094	0	0	2008	407.3576
431	117	2	2004	2	47	0	0	142.5973	6.1	2.4282	1.2141	1	1.2141	2703	399.4377
432	117	3	2006	2	49	0	0	233.8093	3.3	2.4282	0.4047	1	0.4047	3077	510.4249
433	118	1	2002	2	62	10	0	125.634	5.4	4.047	1.2141	0	0	2731	525.3355
434	118	2	2004	2	64	10	0	475.2274	3.7	5.6658	1.6188	1	1.6188	3252	444.505
435	118	3	2006	2	66	10	0	559.1185	5.6	4.047	1.6188	1	1.6188	2719	361.8229
436	118	4	2008	2	68	10	552.6389	295.5979	7.6	4.047	1.6188	1	1.6188	2286	325.8593
437	119	1	2002	2	45	7	1160.542	79.03159	3.7	0.8094	0.8094	0	0	3395	1211.819
438	119	2	2004	2	47	7	644.7453	401.4812	6.2	3.2376	0.8094	0	0	2737	615.7474
439	119	3	2006	2	49	7	0	371.0546	3.7	3.2376	1.2141	1	1.2141	3938	796.2694

440	119	4	2008	2	51	7	3684.259	957.5684	3.8	2.0235	1.6188	1	1.6188	4107	948.7973
441	120	1	2002	2	50	2	0	74.40295	4.4	1.2141	2.0235	0	0	2482	439.1789
442	120	2	2004	2	52	2	0	222.7012	4.6	1.2141	0.8094	1	0.8094	3201	415.1948
443	120	3	2006	2	54	2	0	303.0357	2.8	0.8094	0.8094	1	0.8094	3179	318.113
444	120	4	2008	2	56	2	110.5278	384.4886	3.6	1.2141	0.4047	1	0.4047	3244	397.8103
445	121	1	2002	2	60	7	0	149.364	2.8	2.4282	1.2141	0	0	2902	468.4556
446	121	2	2004	2	62	7	0	162.5739	6.6	2.4282	1.6188	0	0	2763	376.5066
447	121	3	2006	2	64	7	0	201.4167	3.6	1.2141	0.8094	1	0.8094	2780	498.8136
448	121	4	2008	2	66	7	0	292.6047	3.6	2.4282	1.2141	1	1.2141	3030	354.934
449	122	1	2002	2	60	1	0	116.7638	7.3	3.2376	0.60705	0	0	4098	664.6006
450	122	2	2004	2	62	1	0	492.5042	9.2	7.2846	2.4282	1	2.4282	3495	247.3157
451	122	3	2006	2	64	1	0	246.7281	3.8	1.6188	1.6188	1	1.6188	3716	422.2534
452	122	4	2008	2	66	1	0	333.7278	4.6	3.6423	1.82115	1	1.82115	2735	384.5604
453	123	1	2002	2	55	7	198.95	100.0759	6	3.2376	0.4047	0	0	3079	950.733
454	123	2	2004	2	57	7	442.1111	636.4825	3.8	4.4517	0.8094	1	0.8094	4311	1418.084
455	123	3	2006	2	59	7	0	283.562	3.8	1.6188	0.4047	1	0.4047	4061	519.2433
456	123	4	2008	2	61	7	64.47453	377.8992	8.1	1.2141	1.2141	1	1.2141	3410	748.3401
457	124	1	2002	2	60	10	2210.555	95.5913	4.1	0.4047	1.41645	0	0	2634	371.3521
458	124	2	2004	2	62	10	18421.29	354.0246	1.8	8.094	5.6658	1	5.6658	4064	711.3236
459	124	3	2006	2	64	10	921.0648	386.5527	5.3	0	8.094	1	8.094	3443	539.3114
460	124	4	2008	2	66	10	2578.981	1323.063	3.4	0	11.3316	1	11.3316	4553	1577.49
461	125	1	2002	2	60	5	663.1666	189.208	3.3	4.8564	0.8094	1	0.8094	3066	473.5411
462	125	2	2004	2	62	5	0	758.8677	6.8	4.8564	1.6188	1	1.6188	3887	355.3303
463	125	3	2006	2	64	5	0	241.3942	8.2	3.2376	2.4282	1	2.4282	3586	544.2463
464	125	4	2008	2	66	5	0	531.9027	9.9	4.8564	2.4282	1	2.4282	1913	429.8401
465	126	1	2002	2	60	10	0	383.6685	5.8	48.564	12.141	1	12.141	3911	479.9052
466	126	2	2004	2	62	10	0	1248.446	5.7	40.47	22.2585	1	10.1175	4252	502.9938
467	126	3	2006	2	64	10	3684.259	612.0231	5.4	14.1645	4.047	1	4.047	3812	611.2981
468	126	4	2008	2	66	10	552.6389	1058.06	5.2	14.1645	16.188	1	16.188	3841	641.806
469	127	1	2002	2	60	6	663.1666	135.8002	3.6	1.6188	0.8094	0	0	3250	598.2375
470	128	1	2002	2	65	8	0	125.7581	6.2	4.8564	1.01175	0	0	3380	983.4554
471	128	2	2004	2	67	8	0	1164.961	5.8	4.8564	1.2141	1	1.719975	3183	346.1462
472	128	3	2006	2	69	8	0	567.1739	6.6	4.8564	3.2376	1	3.2376	3597	874.8763
473	128	4	2008	2	71	8	0	778.2803	7.7	4.8564	4.8564	1	4.8564	2767	655.4867
474	129	1	2002	2	40	10	0	81.43209	3.3	2.22585	0.8094	0	0	2359	351.8075
475	129	2	2004	2	42	10	0	216.3597	3.3	2.4282	1.6188	1	0.8094	3646	230.1526
476	129	3	2006	2	44	10	0	325.4329	2.5	2.4282	1.2141	1	1.2141	3645	719.9368
477	129	4	2008	2	46	10	46.05324	231.7624	4.2	2.4282	1.2141	1	1.2141	2262	932.1028
478	130	1	2002	2	50	10	0	176.8584	4.6	8.094	4.8564	1	4.8564	3587	579.5399
479	130	2	2004	2	52	10	1105.278	389.1769	4.4	8.094	2.0235	1	2.0235	3123	428.7124
480	130	3	2006	2	54	10	0	722.4106	4.1	5.6658	2.0235	1	2.0235	3640	671.3614
481	130	4	2008	2	56	10	322.3727	392.7401	4.3	2.4282	2.0235	1	2.0235	2814	553.1341
482	131	1	2002	2	35	10	0	163.5412	3.2	2.4282	0.60705	0	0	3705	1025.534
483	132	1	2002	2	65	7	0	188.6541	3.8	4.8564	0.8094	0	0	2531	644.3227

484	133	1	2002	2	63	4	2100.028	159.8266	6.4	5.05875	1.2141	1	1.2141	3149	717.7206
485	133	2	2004	2	65	4	0	260.9628	6.3	10.1175	3.2376	1	1.6188	2992	423.8406
486	133	3	2006	2	67	4	0	1008.179	6.6	7.2846	2.0235	1	2.0235	3226	414.8444
487	133	4	2008	2	69	4	3979	954.5679	5.9	8.094	3.2376	1	3.2376	3143	600.4467
488	134	1	2002	2	52	10	0	223.7998	3.8	4.047	0.60705	1	0.4047	2665	361.3003
489	134	2	2004	2	54	10	0	424.7044	3.8	3.6423	1.2141	1	4.047	4389	712.4279
490	134	3	2006	2	56	10	0	375.0843	3.8	4.047	0.8094	1	0.8094	3532	750.6605
491	134	4	2008	2	58	10	736.8518	802.6473	4.6	3.2376	1.01175	1	1.01175	3577	288.5126
492	135	1	2002	2	60	7	0	202.2979	5.2	6.4752	1.6188	0	0	3610	1015.096
493	135	2	2004	2	62	7	1105.278	391.7781	4.4	6.8799	2.0235	1	2.0235	3152	501.6153
494	135	3	2006	2	64	7	0	438.0594	6.8	6.4752	2.8329	1	2.8329	2944	360.7399
495	135	4	2008	2	66	7	3684.259	872.9673	5.2	6.8799	2.8329	1	2.8329	3361	458.3753
496	136	1	2002	2	63	9	1657.917	165.2647	3.6	6.0705	1.01175	0	0	2622	526.4669
497	136	2	2004	2	65	9	0	224.1278	6.1	5.6658	0.8094	1	0.8094	3151	410.264
498	136	3	2006	2	67	9	0	469.9258	9	5.2611	1.2141	1	1.2141	2493	277.3841
499	136	4	2008	2	69	9	2763.194	814.4523	9.2	5.2611	2.0235	1	2.0235	3612	522.6888
500	137	1	2002	2	50	15	0	93.68076	5.4	2.8329	1.2141	0	0	2559	701.109
501	137	2	2004	2	52	15	0	541.4461	4.8	4.4517	1.6188	1	0.505875	3434	560.3923
502	137	3	2006	2	54	15	0	320.8095	4.6	6.8799	2.4282	1	2.4282	3484	665.0491
503	137	4	2008	2	56	15	18.4213	380.4362	4.4	6.8799	2.8329	1	2.8329	3119	452.3836
504	138	1	2002	2	38	10	0	94.96101	7.9	4.047	0.8094	0	0	2673	477.9719
505	138	2	2004	2	40	10	0	136.1073	8.5	5.2611	0.8094	1	0.8094	2758	296.786
506	138	3	2006	2	42	10	0	233.074	4	4.8564	1.2141	1	1.2141	3066	447.4755
507	138	4	2008	2	44	10	0	533.3016	9.7	4.8564	1.6188	1	1.6188	3253	367.8784
508	139	1	2002	2	75	3	884.2222	142.0845	6	2.4282	0.60705	0	0	2391	638.1901
509	139	2	2004	2	77	3	0	322.201	4.6	2.0235	0.60705	1	0.60705	3438	399.2507
510	139	3	2006	2	79	3	0	191.5359	9	2.4282	0.8094	1	0.8094	2594	287.6215
511	140	1	2002	2	55	0	0	93.59869	5	0.8094	0.12141	0	0	2614	545.6755
512	140	2	2004	2	57	0	0	160.3456	8.5	7.2846	1.01175	1	0.8094	2740	299.1473
513	140	3	2006	2	59	0	0	192.4983	4.1	1.6188	0.4047	1	0.4047	2150	386.1866
514	141	1	2002	2	38	2	773.6944	236.8055	3.3	1.2141	0.8094	0	0	3966	363.063
515	141	2	2004	2	40	2	0	154.0473	3.4	1.2141	0.4047	1	0.4047	3314	404.3073
516	141	3	2006	2	42	2	0	369.2489	3.5	1.6188	0.8094	1	0.8094	3293	572.8591
517	141	4	2008	2	44	2	460.5324	474.4046	3.4	1.41645	0.12141	1	0.12141	3798	706.3876
518	142	1	2002	2	38	10	0	312.8163	7.8	4.047	1.6188	1	0.4047	4052	604.2613
519	142	2	2004	2	40	10	0	681.4916	8.7	7.2846	3.2376	0	0	2339	394.8101
520	143	1	2002	2	43	2	221.0555	92.67132	7.9	1.2141	1.2141	1	0.4047	3209	332.7675
521	143	2	2004	2	45	2	442.1111	293.6505	6.6	1.2141	1.2141	0	0	1979	557.4598
522	143	3	2006	2	47	2	0	240.7716	7.8	1.2141	0.4047	0	0	1807	271.8024
523	144	1	2002	2	35	0	396.0578	184.284	13.7	2.4282	1.2141	0	0	2216	279.9127
524	144	2	2004	2	37	0	276.3194	695.7253	14.8	3.2376	0.8094	1	0.8094	2601	593.1701
525	144	3	2006	2	39	0	2210.555	353.1639	7.2	4.047	1.2141	1	1.2141	4250	449.7179
526	145	1	2002	2	47	10	0	195.4863	8	10.1175	0.8094	0	0	2086	296.6571
527	145	2	2004	2	49	10	0	115.0523	7.1	4.8564	0.8094	0	0	2697	264.8382

528	146	1	2002	2	66	0	55.26389	127.1547	6.9	0.4047	0.4047	0	0	1868	235.9484
529	146	3	2006	2	70	0	0	159.8574	5.6	0.4047	0.4047	1	0.4047	2903	549.3436
530	147	1	2002	2	60	7	0	108.0646	9.1	2.0235	0.4047	0	0	2318	386.8344
531	147	2	2004	2	62	7	0	178.0102	8.2	3.2376	2.0235	0	0	2669	335.8235
532	147	3	2006	2	64	7	0	451.5741	2.8	2.8329	0.8094	0	0	2639	320.757
533	148	1	2002	2	60	0	16.57917	216.1797	8.9	2.4282	1.01175	0	0	2486	391.2264
534	148	2	2004	2	62	0	0	524.3292	8.9	4.4517	2.0235	0	0	4007	440.9859
535	149	1	2002	2	45	0	66.31667	190.9192	6.6	3.2376	2.0235	0	0	3277	544.8613
536	149	2	2004	2	47	0	276.3194	334.6749	3.5	1.6188	1.2141	0	0	2667	803.7255
537	149	3	2006	2	49	0	0	512.053	3.3	0.8094	0	0	0	2960	446.1398
538	150	1	2002	2	40	10	27.63194	112.8182	9.9	2.4282	0.4047	0	0	2415	361.5582
539	150	2	2004	2	42	10	0	223.2833	8.8	3.2376	1.2141	1	0.8094	2937	278.4372
540	151	1	2002	2	55	0	22.10555	178.3332	9.1	0	8.094	1	8.094	2576	721.7688
541	151	2	2004	2	57	0	0	1432.015	4.6	8.4987	4.8564	1	3.2376	2780	765.8076
542	152	1	2002	2	50	15	0	790.5621	4.7	14.1645	4.047	1	0.8094	2766	391.0751
543	152	2	2004	2	52	15	0	566.697	5.8	16.188	4.047	0	0	1977	362.9954
544	152	3	2006	2	54	15	0	546.6517	4.2	10.5222	4.047	1	4.047	4163	778.5193
545	152	4	2008	2	56	15	1768.444	509.8855	4.8	8.9034	3.2376	1	3.2376	2740	576.2552
546	153	1	2002	2	48	10	1127.383	182.6426	5.2	11.7363	4.047	0	0	3147	552.0635
547	153	2	2004	2	50	10	663.1666	360.7458	4.6	8.094	3.6423	0	0	2577	361.5049
548	153	3	2006	2	52	10	1326.333	260.5502	5	12.141	6.0705	1	6.0705	3228	415.9517
549	153	4	2008	2	54	10	1105.278	686.0115	3.4	12.141	4.047	1	4.047	3337	596.7968
550	154	1	2002	2	34	15	552.6389	110.9889	4.2	2.4282	1.2141	0	0	2941	537.5892
551	155	1	2002	2	46	12	644.7453	130.724	6	18.2115	1.6188	0	0	3350	561.6461
552	155	2	2004	2	48	12	0	175.8375	2.8	8.9034	3.2376	0	0	2795	361.9063
553	155	3	2006	2	50	12	0	212.6018	3.6	16.188	1.6188	1	0.8094	4279	747.0003
554	156	1	2002	2	60	3	0	195.2975	2.7	8.094	2.4282	0	0	3449	539.3274
555	156	2	2004	2	62	3	0	292.6785	4.1	6.0705	2.0235	0	0	3040	303.8489
556	156	3	2006	2	64	3	0	236.444	5.2	8.094	3.2376	1	3.2376	2722	410.1458
557	156	4	2008	2	66	3	2210.555	700.6769	5.8	10.1175	2.8329	1	2.8329	4649	625.9717
558	157	1	2002	2	35	2	0	291.7952	3.6	8.9034	2.4282	0	0	3318	564.7615
559	157	2	2004	2	37	2	0	243.444	7.4	8.094	2.8329	0	0	3232	464.1366
560	157	4	2008	2	41	2	2210.555	482.7164	7.9	8.094	1.6188	1	1.6188	3045	391.7796
561	158	1	2002	2	70	4	110.5278	212.105	3.8	4.047	1.6188	0	0	3331	786.2722
562	158	4	2008	2	76	4	92.10648	206.731	4.6	0	2.4282	0	0	2808	312.6028
563	159	1	2002	2	35	5	736.8518	166.4341	3.3	6.0705	0.8094	0	0	3190	90.60606
564	159	2	2004	2	37	5	0	181.4006	6	6.0705	1.6188	0	0	2581	501.5325
565	159	3	2006	2	39	5	1547.389	210.0692	6.5	10.1175	2.0235	1	2.0235	3158	600.6438
566	159	4	2008	2	41	5	2763.194	982.8361	4.8	10.1175	2.0235	1	2.0235	4029	867.7795
567	160	1	2002	2	68	0	1326.333	351.0381	9.1	7.08225	4.4517	0	0	3828	498.7834
568	160	2	2004	2	70	0	663.1666	331.3	6.5	13.3551	4.047	0	0	2968	223.275
569	161	1	2002	2	53	1	110.5278	386.5408	7	44.517	6.0705	1	1.6188	4456	681.9399
570	161	2	2004	2	55	1	0	346.2938	3.6	8.094	1.2141	0	0	2844	215.2161
571	161	3	2006	2	57	1	0	660.4722	4.2	8.094	2.0235	1	2.0235	3991	642.5673

572	161	4	2008	2	59	1	736.8518	1050.075	6.8	14.1645	4.047	1	4.047	4225	462.3128
573	162	1	2002	2	42	16	7736.944	668.691	3.2	36.423	5.6658	1	2.4282	4140	390.2841
574	162	2	2004	2	44	16	644.7453	940.2456	10.9	46.9452	366.2535	0	0	2905	449.3144
575	162	3	2006	2	46	16	1105.278	1141.983	14.3	35.2089	1.6188	1	1.6188	3808	817.768
576	162	4	2008	2	48	16	9947.499	1289.145	15.8	16.188	2.4282	1	2.4282	4259	430.3637
577	163	1	2002	2	40	12	0	222.9288	6	8.094	1.6188	1	0.8094	3151	390.2322
578	164	1	2002	2	68	1	0	162.5441	4.6	16.188	4.8564	0	0	3105	309.2025
579	164	2	2004	2	70	1	0	170.4411	5.8	5.6658	2.4282	0	0	2901	312.8123
580	164	4	2008	2	74	1	0	719.3115	4.6	8.094	4.047	1	4.047	3065	1088.993
581	165	1	2002	2	43	0	442.1111	285.0499	12.1	11.3316	7.2846	0	0	3380	638.1591
582	165	2	2004	2	45	0	0	791.2687	4.8	1.6188	0.8094	0	0	2787	706.4884
583	165	3	2006	2	47	0	0	228.4237	4.8	8.094	2.8329	1	2.8329	3031	622.2524
584	165	4	2008	2	49	0	0	1023.966	16.5	10.1175	1.2141	1	1.2141	2767	359.1496
585	166	1	2002	2	52	0	0	105.7971	7.2	9.3081	4.4517	0	0	3919	883.4485
586	166	2	2004	2	54	0	0	345.5106	8.2	8.9034	2.4282	0	0	2735	310.2953
587	166	3	2006	2	56	0	0	201.1244	2.8	10.1175	8.094	1	4.047	3790	503.1187
588	167	1	2002	2	63	0	0	184.5165	10.6	8.094	1.6188	0	0	2456	379.8486
589	167	2	2004	2	65	0	663.1666	143.9291	4.6	26.7102	1.2141	0	0	1738	354.4536
590	167	4	2008	2	69	0	0	285.6021	5	2.0235	1.2141	1	1.2141	3036	637.348
591	168	1	2002	2	46	15	596.85	153.7731	3.3	3.2376	1.2141	0	0	3042	693.7819
592	168	2	2004	2	48	15	663.1666	244.2747	4.2	5.6658	1.2141	0	0	2762	388.0755
593	169	1	2002	2	32	2	55.26389	92.08043	6.3	0.8094	0.8094	1	0.8094	2740	323.7974
594	169	2	2004	2	34	2	0	139.7605	2.8	6.4752	0.8094	0	0	2307	332.2496
595	170	1	2002	2	69	7	1901.078	254.9028	8.4	4.8564	1.112925	1	1.112925	3930	691.9574
596	170	2	2004	2	71	7	368.4259	173.7608	8.5	4.047	1.6188	0	0	2150	279.469
597	170	3	2006	2	73	7	1016.855	399.8458	5.7	5.2611	1.2141	1	1.2141	3415	457.9681
598	170	4	2008	2	75	7	3536.889	604.4623	7.3	5.6658	1.6188	1	1.6188	3834	757.5054
599	171	1	2002	2	49	16	0	799.9285	7.3	16.188	2.0235	1	1.2141	4388	1056.409
600	171	2	2004	2	51	16	552.6389	279.3088	4.3	4.4517	4.8564	1	2.4282	4389	441.9586
601	172	1	2002	2	45	10	221.0555	109.5883	7.4	4.047	1.41645	1	0.4047	3111	524.2714
602	172	2	2004	2	47	10	0	198.7166	7.8	2.8329	1.6188	0	0	2235	310.2774
603	172	3	2006	2	49	10	0	204.4324	7.6	2.8329	1.6188	1	1.6188	2968	437.206
604	172	4	2008	2	51	10	0	397.5576	6.2	2.8329	1.2141	1	1.2141	2622	602.1644
605	173	1	2002	2	50	10	2247.398	248.6664	11	11.7363	2.8329	0	0	2603	283.7318
606	173	2	2004	2	52	10	0	1260.497	5.1	12.141	2.0235	0	0	2171	460.0455
607	173	3	2006	2	54	10	921.0648	810.434	4.8	12.141	1.6188	1	1.6188	3713	569.601
608	173	4	2008	2	56	10	0	678.1315	5.2	12.141	0.48564	1	0.48564	3987	620.0963
609	174	1	2002	2	50	0	368.4259	287.0743	4.8	3.6423	3.2376	0	0	2435	341.1335
610	174	2	2004	2	52	0	0	396.1422	5.6	14.1645	1.2141	0	0	2348	296.9693
611	175	1	2002	2	50	5	0	80.50882	4.2	1.6188	0.8094	0	0	3582	241.0842
612	175	2	2004	2	52	5	0	231.5371	5.2	4.8564	1.2141	0	0	2173	374.0752
613	176	1	2002	2	30	16	276.3194	142.4468	6	6.0705	2.0235	0	0	3118	301.3698
614	176	2	2004	2	32	16	405.2685	244.0996	3.2	3.6423	0.4047	0	0	2835	532.4518
615	177	1	2002	2	36	15	921.0648	343.7997	7.3	12.5457	0.4047	1	0.4047	3624	606.4723

616	177	2	2004	2	38	15	0	336.3802	7.9	12.5457	2.0235	1	2.0235	3100	311.3288
617	177	3	2006	2	40	15	5157.962	859.2894	8.1	12.5457	2.4282	1	2.4282	4498	1088.492
618	177	4	2008	2	42	15	9210.647	1139.099	7.3	12.5457	0.8094	1	0.8094	3632	813.2213
619	178	1	2002	2	45	10	0	719.7374	5.3	0	4.047	1	2.0235	4527	1053.561
620	179	1	2002	2	50	12	0	941.7316	3.8	0	2.0235	1	2.0235	3663	1183.637
621	180	1	2002	2	50	10	92.10648	280.8207	4.6	4.047	1.2141	0	0	2667	616.4586
622	181	1	2002	2	60	0	46.05324	197.3738	5.4	2.0235	1.2141	0	0	2891	706.1698
623	181	2	2004	2	62	0	0	145.5662	8.1	4.8564	0.8094	0	0	2056	231.9032
624	182	1	2002	2	40	5	331.5833	287.4436	4.6	3.2376	1.6188	0	0	3444	694.6214
625	182	2	2004	2	42	5	221.0555	278.6331	5.2	5.05875	0.60705	0	0	3740	1609.044
626	183	1	2002	2	40	10	0	278.5392	2.8	2.8329	0.8094	0	0	3392	275.5154
627	183	2	2004	2	42	10	1989.5	415.8708	6.6	8.9034	1.6188	0	0	3227	519.645
628	184	1	2002	2	55	12	884.2222	333.6069	5.2	4.047	2.0235	0	0	3213	513.0301
629	184	2	2004	2	57	12	0	213.4425	4.1	6.0705	1.2141	0	0	2103	565.3781
630	185	1	2002	2	60	0	442.1111	172.0353	6.7	1.6188	0.4047	0	0	3099	715.0027
631	185	2	2004	2	62	0	0	546.6576	10.3	16.188	5.6658	0	0	2914	410.9135
632	186	1	2002	2	41	10	176.8444	119.6143	5.1	2.0235	0.8094	0	0	2591	455.4673
633	186	2	2004	2	43	10	0	1995.597	7.6	30.3525	12.141	1	12.141	4244	1103.025
634	187	1	2002	2	65	5	1326.333	661.5585	12.5	28.329	1.6188	1	1.6188	3693	1244.805
635	187	3	2006	2	67	5	0	1381.785	16.1	24.282	8.094	1	8.094	3766	786.3207
636	188	1	2002	2	29	17	0	223.0717	8.2	12.141	2.4282	0	0	3485	940.6083
637	188	2	2004	2	31	17	0	256.4984	10.8	5.969325	1.2141	0	0	2085	230.9494
638	188	3	2006	2	33	17	0	229.7279	2.2	4.047	1.6188	1	1.6188	4495	785.5922
639	188	4	2008	2	35	17	0	313.1411	7.7	6.4752	2.4282	1	2.4282	4142	739.248
640	189	1	2002	2	36	10	552.6389	412.3086	6.4	4.047	3.2376	0	0	3110	866.7258
641	189	2	2004	2	38	10	0	188.1602	5.9	14.1645	2.0235	1	1.01175	3685	858.1433
642	189	3	2006	2	40	10	92.10648	301.0944	4.4	4.047	1.2141	1	1.2141	4420	1098.013
643	189	4	2008	2	42	10	921.0648	401.4228	4.6	4.047	2.0235	1	2.0235	3795	640.0457
644	190	1	2002	2	42	7	1326.333	274.7733	8.9	8.094	1.2141	0	0	3344	1013.465
645	190	2	2004	2	44	7	0	301.8039	4.5	28.329	12.141	1	12.141	3515	404.975
646	190	3	2006	2	46	7	921.0648	440.6847	6	8.9034	3.2376	1	3.2376	3242	489.0197
647	190	4	2008	2	48	7	7000.092	872.0231	6.1	8.094	3.2376	1	3.2376	4633	1066.443
648	191	1	2002	2	40	10	221.0555	237.9389	7.6	12.141	2.0235	0	0	3448	1029.269
649	191	2	2004	2	42	10	0	121.2199	8.5	8.094	1.6188	0	0	2825	150.9691
650	191	3	2006	2	44	10	0	432.1115	4.6	7.2846	0.8094	1	0.8094	4600	589.4988
651	191	4	2008	2	46	10	221.0555	625.1993	9.1	7.2846	1.2141	1	1.2141	3784	659.7092
652	192	1	2002	2	35	7	0	123.6544	6.2	4.8564	2.4282	0	0	2486	451.6283
653	192	2	2004	2	37	7	0	320.9413	6.8	4.8564	1.6188	1	1.6188	3456	447.1163
654	193	1	2002	2	35	10	0	135.3416	7.1	4.047	4.047	0	0	2426	550.7783
655	194	1	2002	2	45	0	128.9491	490.5622	5.1	0	2.4282	1	1.2141	4199	1516.33
656	195	1	2002	2	45	0	0	615.5109	4.3	0	3.2376	0	0	2431	511.3957
657	196	1	2002	2	60	0	0	382.2322	5.6	0	2.0235	0	0	3884	1335.408
658	197	1	2002	2	45	7	0	353.057	5.9	0	1.2141	0	0	3822	1615.558
659	198	1	2002	2	50	2	184.213	565.9007	5.6	0	1.6188	0	0	3795	1801.553

660	199	1	2002	2	50	7	0	486.2536	6.1	0	4.047	1	2.0235	3261	826.8201
661	200	1	2002	2	50	10	0	208.0427	3.4	0	2.0235	1	2.0235	4692	844.8871
662	202	1	2002	2	45	0	0	239.2922	3.6	0	3.2376	1	0.4047	4383	2110.512
663	202	2	2004	2	47	0	0	152.633	3.5	4.8564	3.2376	0	0	2387	713.1755
664	202	3	2006	2	49	0	0	357.6493	4.4	0	2.4282	1	2.4282	2825	608.9298
665	203	1	2002	2	45	7	0	249.4887	4.6	0	4.047	1	1.2141	4604	2063.089
666	203	2	2004	2	47	7	0	233.106	4.4	6.0705	4.4517	1	4.4517	3257	1079.511
667	203	3	2006	2	49	7	0	887.9244	1.8	0	4.047	1	4.047	4644	1832.193
668	203	4	2008	2	51	7	0	546.8037	1.8	0	2.0235	1	2.0235	4249	1460.017
669	204	1	2002	2	45	10	0	142.0599	8.1	0	1.6188	0	0	2351	487.4547
670	204	2	2004	2	47	10	0	137.7444	4.1	4.047	0.8094	0	0	1758	283.6854
671	205	1	2002	2	65	7	0	135.835	5.7	8.094	0.8094	0	0	2341	419.9643
672	205	2	2004	2	67	7	0	1234.379	4.4	4.8564	3.2376	1	1.6188	3589	389.8643
673	205	3	2006	2	69	7	368.4259	293.969	4	4.047	4.047	1	4.047	3332	521.3226
674	205	4	2008	2	71	7	0	352.2687	1.8	4.4517	8.094	1	8.094	3961	482.0982
675	206	1	2002	2	38	5	0	145.1656	5.9	0	2.4282	0	0	3122	1488.834
676	206	2	2004	2	40	5	0	743.3379	1.8	2.63055	1.82115	1	1.2141	4243	1598.677
677	206	3	2006	2	42	5	0	457.8367	1.8	0	2.8329	1	2.8329	3870	967.5978
678	206	4	2008	2	44	5	0	540.9125	1.8	0	3.2376	1	3.2376	4369	1718.103
679	207	1	2002	2	30	10	0	86.28094	7.4	4.047	0.8094	0	0	1796	362.7367
680	207	2	2004	2	32	10	0	325.5662	4	4.047	1.6188	0	0	3244	522.5963
681	208	1	2002	2	45	0	0	103.797	7.1	0	1.2141	0	0	1915	344.8441
682	208	2	2004	2	47	0	0	822.874	1.8	16.188	6.0705	1	1.6188	2840	538.2186
683	209	1	2002	2	32	7	0	129.565	6.8	0	2.0235	0	0	3428	1844.015
684	209	3	2006	2	36	7	0	481.835	4	0	4.047	0	0	2809	676.9048
685	209	4	2008	2	38	7	0	400.4111	4.6	0	4.047	1	4.047	3329	962.92
686	210	1	2002	3	45	5	128.9491	275.5827	3.7	1.6188	0.4047	0	0	2040	709.6915
687	210	2	2004	3	47	5	184.213	391.1437	3.6	2.4282	1.6188	1	1.6188	2757	385.4069
688	210	3	2006	3	49	5	0	801.9489	2.6	1.6188	2.8329	1	2.8329	4228	1290.764
689	210	4	2008	3	51	5	0	804.3787	2.6	1.6188	2.4282	1	2.4282	4644	1366.53
690	211	1	2002	3	27	10	736.8518	176.7255	2.8	0.8094	2.0235	1	0.4047	3180	866.6
691	212	1	2002	3	40	0	663.1666	230.5704	3.5	7.2846	3.6423	0	0	2395	943.1387
692	212	2	2004	3	42	0	0	311.9751	5	4.047	2.4282	1	2.4282	3049	883.9486
693	212	3	2006	3	44	0	1289.491	236.8338	4.8	7.6893	4.047	1	4.047	3911	755.8844
694	212	4	2008	3	46	0	386.8472	385.7483	6.3	7.2846	5.2611	1	5.2611	3417	602.3232
695	213	1	2002	3	50	2	368.4259	337.7349	2.8	2.8329	1.2141	0	0	2407	1030.699
696	213	2	2004	3	52	2	0	248.829	3.6	2.4282	1.2141	1	1.41645	2813	517.6821
697	213	3	2006	3	54	2	0	478.7587	4.4	2.4282	1.2141	1	1.2141	2912	934.8854
698	213	4	2008	3	56	2	0	652.8954	2.6	2.8329	1.6188	1	1.6188	4483	1140.494
699	214	1	2002	3	55	0	331.5833	646.1978	4.2	6.0705	2.4282	1	0.60705	3439	970.0156
700	214	2	2004	3	57	0	515.7963	594.3247	4	3.2376	1.6188	1	1.6188	3449	689.6157
701	215	1	2002	3	36	7	0	450.8309	3.6	3.2376	3.2376	0	0	2766	727.1361
702	215	2	2004	3	38	7	147.3704	1052.797	1.8	4.8564	1.6188	1	1.6188	4003	1725.919
703	215	3	2006	3	40	7	0	1229.481	3.5	3.2376	1.6188	1	1.6188	4290	1037.911

704	215	4	2008	3	42	7	27.66879	2456.405	1.8	2.8329	4.047	1	4.047	4371	1232.893
705	216	1	2002	3	38	5	0	182.792	4.3	5.2611	2.0235	0	0	2856	1039.422
706	216	2	2004	3	40	5	0	464.4839	3.7	6.0705	3.2376	1	0.8094	4047	1709.102
707	216	3	2006	3	42	5	0	970.8793	3.6	4.8564	3.2376	1	3.2376	3278	545.163
708	216	4	2008	3	44	5	1842.13	824.2667	3.6	5.2611	3.6423	1	3.6423	4477	1363.214
709	217	1	2002	3	55	2	607.9027	869.0767	2.6	5.2611	0.4047	0	0	2591	352.401
710	217	2	2004	3	57	2	0	459.2087	3.6	2.22585	1.82115	1	0.8094	3323	640.5717
711	217	3	2006	3	59	2	0	381.6595	4.6	1.2141	1.2141	1	1.2141	2074	575.8433
712	217	4	2008	3	61	2	73.68518	707.3076	3.6	1.6188	0.4047	1	0.4047	3326	647.7847
713	218	1	2002	3	22	0	0	173.6048	3.6	2.0235	2.0235	0	0	1704	344.8375
714	218	2	2004	3	24	0	0	222.4723	4.4	3.43995	2.22585	1	1.2141	3141	920.4495
715	218	3	2006	3	26	0	0	401.664	2.8	2.4282	3.2376	1	3.2376	4224	876.2928
716	218	4	2008	3	28	0	0	398.9256	5.2	2.22585	3.03525	1	3.03525	3186	674.2429
717	219	1	2002	3	31	3	0	206.1567	3.5	2.0235	1.6188	1	0.4047	3741	402.8882
718	219	2	2004	3	33	3	0	175.2956	3.1	4.047	2.8329	1	2.0235	3322	675.0109
719	219	3	2006	3	35	3	0	264.0792	3.7	2.0235	3.2376	1	3.2376	3214	820.8032
720	219	4	2008	3	37	3	0	429.08	3.1	2.0235	3.2376	1	3.2376	3748	718.8083
721	220	1	2002	3	35	1	0	854.5178	2.5	8.094	6.0705	1	0.8094	3333	563.9897
722	220	2	2004	3	37	1	0	359.2451	4.4	8.094	0.4047	1	2.0235	2196	465.901
723	220	3	2006	3	39	1	0	318.4812	3.4	8.094	5.6658	1	5.6658	3052	909.9818
724	220	4	2008	3	41	1	0	851.4666	3.6	8.094	6.0705	1	6.0705	4127	1187.747
725	221	1	2002	3	29	3	128.9491	251.2085	3.1	4.047	1.6188	0	0	1944	469.2164
726	221	2	2004	3	31	3	82.89583	260.0091	4.1	1.82115	1.82115	1	0.8094	2481	410.6173
727	221	3	2006	3	33	3	0	231.4279	4.2	8.094	2.0235	1	2.0235	2461	532.4736
728	221	4	2008	3	35	3	0	914.5149	3.3	3.2376	2.4282	1	2.4282	3924	1405.712
729	222	1	2002	3	33	9	128.9491	449.7753	6.1	9.7128	5.2611	1	0.8094	3811	765.9847
730	222	2	2004	3	35	9	92.10648	254.0635	5.6	8.9034	5.6658	1	3.2376	2887	716.4088
731	222	3	2006	3	37	9	0	426.6247	4.7	8.9034	4.047	1	4.047	3871	462.9218
732	222	4	2008	3	39	9	0	562.9767	5.8	8.9034	4.4517	1	4.4517	3984	1093.337
733	223	1	2002	3	30	2	265.2667	528.6647	3.7	0.4047	1.6188	1	0.8094	3135	420.423
734	223	2	2004	3	32	2	55.26389	140.6808	5.8	2.0235	1.6188	0	0	1906	463.8885
735	224	1	2002	3	35	5	368.4259	416.19	3.3	2.4282	3.2376	0	0	2025	633.849
736	225	1	2002	3	24	12	0	853.0858	3	6.0705	0.8094	0	0	3136	1053.59
737	225	2	2004	3	26	12	368.4259	245.407	5.2	0	1.2141	1	1.2141	1923	389.7473
738	225	3	2006	3	28	12	0	265.4823	2.5	4.047	0.8094	1	0.8094	2282	746.8827
739	225	4	2008	3	30	12	184.213	339.471	3.3	4.047	1.2141	1	1.2141	1958	575.095
740	226	1	2002	3	32	0	0	162.2585	3.8	2.8329	2.0235	0	0	2038	492.1382
741	226	2	2004	3	34	0	552.6389	237.226	3.7	2.63055	2.0235	1	0.4047	2732	563.3414
742	226	3	2006	3	36	0	0	437.1486	4	2.0235	1.41645	1	1.41645	3422	1194.927
743	226	4	2008	3	38	0	0	419.0023	4.2	1.41645	1.6188	1	1.6188	3283	873.0767
744	227	1	2002	3	40	0	0	111.8471	5.8	1.2141	2.8329	0	0	1567	177.3618
745	227	2	2004	3	42	0	165.7917	191.7235	4.3	2.4282	1.6188	0	0	1476	216.8195
746	227	3	2006	3	44	0	0	273.7512	6.1	1.2141	3.2376	1	3.2376	3013	604.9905
747	227	4	2008	3	46	0	0	290.4774	5	0	3.2376	1	3.2376	2740	599.8834

748	228	1	2002	3	27	5	0	345.8898	6.6	3.6423	2.0235	0	0	3178	814.5875
749	228	2	2004	3	29	5	36.84259	296.9986	2.4	20.235	0.60705	1	0.60705	3259	828.1454
750	228	4	2008	3	33	5	0	1127.751	1.8	2.0235	0.8094	1	0.8094	3585	941.653
751	229	1	2002	3	40	5	386.8472	374.0829	4.2	0.20235	0.303525	0	0	2365	523.5245
752	229	2	2004	3	42	5	1657.917	613.112	2.8	2.0235	1.6188	1	0.8094	3294	397.6696
753	229	3	2006	3	44	5	0	406.2784	4.3	0.4047	0.8094	1	0.8094	2507	432.6273
754	229	4	2008	3	46	5	221.0555	1146.716	3.6	0.8094	0.8094	1	0.8094	2635	617.6771
755	230	1	2002	3	25	5	0	175.9376	4	1.6188	0.8094	0	0	2594	718.8184
756	230	2	2004	3	27	5	0	726.8911	2.4	2.0235	0.8094	0	0	2981	962.1871
757	230	3	2006	3	29	5	0	547.2753	2.5	1.2141	1.2141	1	1.2141	3596	873.5922
758	230	4	2008	3	31	5	0	1248.266	2.5	1.2141	1.6188	1	1.6188	3707	1057.276
759	231	1	2002	3	40	5	184.213	573.9023	1.8	0.8094	0.8094	0	0	3193	834.7683
760	231	2	2004	3	42	5	276.3194	337.8809	3.7	2.0235	0.8094	1	0.8094	1833	308.313
761	231	3	2006	3	44	5	0	380.0989	3.6	3.2376	1.6188	1	1.6188	3800	1098.158
762	231	4	2008	3	46	5	331.5833	363.4996	2.6	1.6188	1.6188	1	1.6188	3181	882.8845
763	232	1	2002	3	48	4	552.6389	206.4013	4.4	2.8329	1.01175	0	0	2856	616.1848
764	232	2	2004	3	50	4	0	352.035	4.6	3.2376	1.6188	1	1.6188	3168	709.4196
765	232	4	2008	3	54	4	0	403.3091	4.4	1.6188	1.6188	1	1.6188	2234	644.4921
766	233	1	2002	3	45	9	2763.194	1335.314	1.8	3.2376	0.8094	0	0	3590	1372.672
767	233	2	2004	3	47	9	0	805.6844	1.8	4.047	1.6188	1	1.6188	3570	1087.257
768	233	3	2006	3	49	9	0	202.3611	3.4	4.047	2.0235	1	2.0235	2048	616.4954
769	233	4	2008	3	51	9	442.1111	680.9727	1.8	4.047	1.6188	1	1.6188	3474	786.053
770	234	1	2002	3	65	15	0	2163.73	1.8	8.094	4.047	1	0.8094	4555	1729.094
771	234	3	2006	3	69	15	0	1208.005	3.4	8.094	4.047	1	4.047	3623	1196.532
772	235	1	2002	3	46	5	921.0648	641.5388	5.3	24.282	2.0235	1	1.6188	3254	1029.138
773	235	2	2004	3	48	5	3315.833	3157.276	3.6	2.8329	1.2141	1	1.2141	4355	1491.741
774	235	3	2006	3	50	5	921.0648	2024.492	5.9	12.141	2.8329	1	2.8329	3899	971.9152
775	235	4	2008	3	52	5	0	867.5558	4	8.094	2.0235	1	2.0235	3926	1247.547
776	236	1	2002	3	32	0	0	484.5302	9.1	7.48695	4.047	1	0.4047	2523	742.5388
777	236	2	2004	3	34	0	1197.384	520.6276	9	10.1175	5.86815	1	1.2141	4144	1541.739
778	236	3	2006	3	36	0	0	623.2067	3.4	2.0235	1.2141	1	1.2141	4027	1180.188
779	237	1	2002	3	40	10	921.0648	730.151	9.8	12.141	4.047	1	1.2141	4251	1144.042
780	237	2	2004	3	42	10	0	692.301	12.5	11.3316	5.6658	1	4.047	3626	1915.198
781	237	3	2006	3	44	10	0	1184.787	7.4	12.141	6.0705	1	6.0705	3802	1335.488
782	237	4	2008	3	46	10	921.0648	454.6628	3	4.047	0.8094	1	0.8094	3361	1149.735
783	238	1	2002	3	35	2	0	167.1317	2.2	0.8094	0.8094	0	0	2646	821.3425
784	238	2	2004	3	37	2	0	240.8595	2.8	5.46345	4.8564	0	0	3048	745.4938
785	238	4	2008	3	41	2	0	356.2898	3.7	0.60705	0.8094	1	0.8094	2651	636.662
786	239	1	2002	3	50	2	0	361.0341	4	4.047	2.0235	0	0	3588	1386.944
787	239	2	2004	3	52	2	0	1221.889	2.8	6.0705	1.6188	0	0	2355	778.8498
788	239	3	2006	3	54	2	0	776.0137	2.8	6.0705	2.0235	1	2.0235	3543	1103.277
789	239	4	2008	3	56	2	110.5278	760.7721	4.3	6.0705	2.63055	1	2.63055	3812	1377.161
790	240	1	2002	3	50	10	0	410.6689	2.8	9.3081	0.8094	0	0	3171	1353.33
791	240	2	2004	3	52	10	1178.963	1610.232	1.8	2.8329	1.6188	0	0	3106	1009.828

792	240	3	2006	3	54	10	0	1639.592	2.6	4.047	1.2141	1	1.2141	4310	1905.745
793	240	4	2008	3	56	10	0	794.0837	4.3	2.0235	2.0235	1	2.0235	2220	469.6445
794	241	1	2002	3	50	0	505.9593	136.2686	7.3	0.8094	0.4047	0	0	1660	222.2883
795	241	2	2004	3	52	0	143.6861	170.3274	8.4	2.0235	1.41645	0	0	2073	401.7712
796	241	4	2008	3	56	0	0	1114.866	2.5	0.60705	1.2141	1	1.2141	4466	1299.202
797	242	1	2002	3	32	5	921.0648	184.9282	5.1	4.047	1.6188	0	0	2362	776.5945
798	242	2	2004	3	34	5	0	197.8551	5.1	0.8094	0.8094	0	0	2126	373.4825
799	242	3	2006	3	36	5	0	406.2739	3.6	3.2376	0.8094	1	0.8094	3355	1131.432
800	243	1	2002	3	35	5	0	724.8995	3.6	8.094	4.047	0	0	3160	1390.227
801	243	2	2004	3	37	5	0	681.537	4.2	8.094	3.6423	1	1.2141	4209	1418.162
802	244	1	2002	3	37	10	552.6389	322.0008	4	3.6423	1.01175	1	0.8094	3831	1160.479
803	244	2	2004	3	39	10	464.2166	668.0764	3	2.8329	0.8094	1	0.8094	3489	649.2388
804	244	3	2006	3	41	10	663.1666	665.28	3.2	1.01175	1.01175	1	1.01175	4377	1415.262
805	244	4	2008	3	43	10	795.7999	831.3262	4.1	2.8329	1.01175	1	1.01175	3239	708.5979
806	245	1	2002	3	38	0	663.1666	160.3406	3.1	0.60705	0.60705	1	1.6188	3757	781.8879
807	245	2	2004	3	40	0	55.26389	172.4875	3.2	2.22585	1.6188	1	0.8094	3009	469.2322
808	245	4	2008	3	44	0	368.4259	184.0729	2.3	0.4047	0.4047	1	0.4047	3354	736.4962
809	246	1	2002	3	35	15	0	152.5975	3.7	1.6188	0.4047	0	0	2929	870.3493
810	246	2	2004	3	37	15	18.4213	154.1883	3.8	1.6188	0.8094	1	0.4047	2322	804.0052
811	246	3	2006	3	39	15	0	226.9395	3.9	2.0235	0.8094	1	0.8094	2523	539.4636
812	246	4	2008	3	41	15	0	432.6498	4	2.0235	1.2141	1	1.2141	2238	700.2849
813	247	1	2002	3	30	10	276.3194	271.3788	3.2	2.0235	1.6188	1	0.60705	3808	1171.43
814	247	2	2004	3	32	10	0	207.2078	3.2	3.6423	1.6188	1	0.4047	3560	871.8337
815	247	3	2006	3	34	10	0	467.9784	3.3	2.0235	2.0235	1	2.0235	4316	1136.309
816	248	1	2002	3	40	0	0	255.5109	4.4	1.82115	0.8094	1	0.4047	3302	862.3769
817	248	2	2004	3	42	0	460.5324	155.2329	5.4	1.82115	0.8094	1	0.4047	2783	606.5747
818	248	3	2006	3	44	0	0	492.5118	3.6	2.0235	0.8094	1	0.8094	3116	1164.072
819	249	1	2002	3	23	10	184.213	234.92	2.2	0.8094	1.6188	1	0.60705	4036	1006.219
820	249	3	2006	3	27	10	0	461.5471	2.8	1.2141	0.8094	1	0.8094	4551	1442.128
821	249	4	2008	3	29	10	0	415.8937	3.1	1.2141	1.2141	1	1.2141	3728	790.5541
822	250	1	2002	3	30	10	0	205.1703	2.4	0.8094	0.4047	0	0	3026	1016.161
823	250	2	2004	3	32	10	9.210648	214.8704	2.4	0.4047	0.4047	1	0.4047	2639	569.4654
824	250	4	2008	3	36	10	1842.13	1128.754	3.2	0.8094	1.01175	1	1.01175	4243	1673.15
825	251	1	2002	3	60	0	0	190.3775	5.1	1.6188	1.6188	0	0	2761	841.8849
826	251	2	2004	3	62	0	0	269.9474	5.8	3.03525	0.8094	1	1.2141	4508	667.7238
827	251	3	2006	3	64	0	0	269.3051	4.9	2.22585	1.2141	1	1.2141	4116	1081.562
828	251	4	2008	3	66	0	0	310.0276	5	2.22585	1.6188	1	1.6188	2623	676.1177
829	252	1	2002	3	32	12	0	257.0989	4.6	2.0235	1.41645	1	0.60705	3298	1007.879
830	252	2	2004	3	34	12	0	116.4165	3.2	1.82115	1.82115	1	1.6188	2408	569.9479
831	252	3	2006	3	36	12	0	203.9352	3.2	2.4282	1.01175	1	1.01175	2500	414.8188
832	252	4	2008	3	38	12	0	292.0782	3.2	1.6188	1.2141	1	1.2141	2754	531.7258
833	253	1	2002	3	26	13	1768.444	247.4969	4.4	4.047	1.2141	0	0	3587	1600.133
834	253	2	2004	3	28	13	221.0555	683.9473	5.3	4.047	2.0235	1	0.8094	2576	767.8948
835	253	3	2006	3	30	13	0	370.6768	4.3	2.4282	1.6188	1	1.6188	3218	739.6806

836	253	4	2008	3	32	13	0	497.2842	6.6	4.047	2.4282	1	2.4282	4423	1087.896
837	254	1	2002	3	55	3	221.0555	281.2269	6.1	4.047	2.0235	1	2.0235	3653	723.059
838	254	2	2004	3	57	3	552.6389	351.5064	6.3	3.6423	2.8329	1	0.8094	2826	1059.118
839	254	3	2006	3	59	3	1657.917	707.9379	4.9	4.8564	1.2141	1	1.2141	3728	679.0234
840	254	4	2008	3	61	3	2947.407	693.2164	7.4	6.4752	3.2376	1	3.2376	3669	926.0544
841	255	1	2002	3	40	0	0	115.8145	4.1	1.6188	1.2141	0	0	2273	453.2191
842	255	3	2006	3	44	0	0	160.2429	5.4	0.8094	0.4047	1	0.4047	2005	572.1614
843	256	1	2002	3	55	5	221.0555	217.5626	4.9	2.8329	1.2141	1	0.8094	3162	795.496
844	256	2	2004	3	57	5	0	180.5886	5.4	2.4282	1.6188	0	0	2640	547.6696
845	256	3	2006	3	59	5	0	111.6901	5.2	1.82115	1.01175	1	1.01175	4167	361.0676
846	256	4	2008	3	61	5	1289.491	363.9383	6.6	2.63055	1.2141	1	1.2141	2567	681.7567
847	257	1	2002	3	75	1	2210.555	285.3403	6.1	4.047	2.63055	1	1.6188	3391	905.4711
848	257	2	2004	3	77	1	552.6389	852.5303	8.4	4.047	2.4282	1	0.8094	3001	972.3538
849	257	3	2006	3	79	1	0	591.0427	5.4	3.2376	1.41645	1	1.41645	3139	761.956
850	257	4	2008	3	81	1	1289.491	617.2975	3.6	4.047	1.6188	1	1.6188	3485	1067.356
851	258	1	2002	3	50	8	184.213	395.0061	5.2	2.8329	0.8094	0	0	1837	741.388
852	258	2	2004	3	52	8	18.4213	285.0083	5.2	3.2376	0.8094	1	0.4047	3874	1201.534
853	258	3	2006	3	54	8	0	273.047	4.5	4.047	1.01175	1	1.01175	3390	621.22
854	258	4	2008	3	56	8	55.26389	796.6074	5.4	3.43995	1.2141	1	1.2141	2716	679.713
855	259	1	2002	3	45	0	0	173.2663	3.6	4.047	2.63055	0	0	2229	1011.509
856	259	2	2004	3	47	0	92.10648	186.5242	5	4.047	2.4282	0	0	2292	696.5821
857	259	3	2006	3	49	0	0	282.2667	4.6	4.047	2.4282	1	2.4282	2502	904.8926
858	259	4	2008	3	51	0	368.4259	479.2178	1.8	4.047	4.047	1	4.047	4501	1385.047
859	260	1	2002	3	30	10	0	182.3451	2.8	2.0235	1.41645	1	1.01175	4278	782.7167
860	260	3	2006	3	34	10	0	365.5723	3.2	1.6188	1.41645	1	1.41645	3805	1200.272
861	260	4	2008	3	36	10	0	599.2354	3.3	1.2141	1.01175	1	1.01175	3640	1218.902
862	261	1	2002	3	45	7	0	122.6713	3.3	0.8094	0.8094	0	0	1916	531.8983
863	261	2	2004	3	47	7	151.0546	176.3756	3.4	0.8094	0.8094	1	0.4047	2820	561.8268
864	261	3	2006	3	49	7	0	214.3557	4	0.8094	0.8094	1	0.4047	2802	477.1467
865	261	4	2008	3	51	7	0	555.9664	3.6	0.8094	2.0235	1	2.0235	3657	1062.654
866	262	1	2002	3	42	2	0	224.446	3.2	2.4282	0.8094	0	0	2280	683.8722
867	262	2	2004	3	44	2	0	321.794	3.2	2.0235	0.60705	0	0	3284	846.369
868	262	3	2006	3	46	2	0	548.6957	3.3	3.2376	0.60705	1	0.60705	3785	1015.929
869	263	1	2002	3	43	12	0	129.1342	5.1	4.8564	0.8094	0	0	2524	727.7279
870	263	2	2004	3	45	12	0	295.548	4.4	4.047	1.2141	1	0.8094	2661	479.1693
871	264	1	2002	3	50	12	0	195.6709	3.6	4.047	0.4047	0	0	3068	1272.314
872	264	2	2004	3	52	12	27.63194	106.2756	3.7	2.8329	1.2141	0	0	2345	349.6592
873	264	3	2006	3	54	12	0	365.4749	2.6	1.2141	0.4047	1	0.4047	4161	1209.665
874	264	4	2008	3	56	12	0	3131.445	2.6	0.4047	1.6188	0	0	2637	588.7073
875	265	1	2002	3	45	0	0	260.3396	7.1	0.8094	0.4047	0	0	1597	439.8814
876	265	2	2004	3	47	0	230.2662	158.9106	7.2	0.8094	0.8094	1	0.8094	2461	652.8199
877	265	3	2006	3	49	0	0	284.7013	5.3	0.8094	2.0235	1	2.0235	4030	1006.706
878	265	4	2008	3	51	0	368.4259	221.1359	6.6	0.8094	0.8094	1	0.8094	2583	352.5448
879	266	1	2002	3	26	10	92.10648	199.1916	3.2	0.60705	0.4047	1	0.4047	3691	696.8101

880	266	2	2004	3	28	10	0	152.0691	2.8	1.82115	0.4047	1	1.2141	3905	1019.297
881	267	1	2002	3	35	10	221.0555	258.2043	3.8	1.2141	0.4047	0	0	3119	882.4484
882	267	2	2004	3	37	10	274.4773	352.6224	5.1	1.2141	0.60705	1	0.60705	3655	1547.131
883	267	3	2006	3	39	10	221.0555	297.2521	4.2	0.8094	0.8094	1	0.8094	2837	445.769
884	267	4	2008	3	41	10	644.7453	526.4979	4.2	1.2141	0.60705	1	0.60705	2514	783.9365
885	268	1	2002	3	46	10	1980.289	158.4399	9.1	4.047	2.0235	1	1.01175	2587	413.3451
886	268	2	2004	3	48	10	1031.593	273.7517	10.8	6.0705	2.4282	1	1.6188	2194	354.4016
887	268	3	2006	3	50	10	0	1147.088	3.4	6.0705	2.4282	1	2.4282	3424	830.4504
888	268	4	2008	3	52	10	663.1666	638.3387	5.2	6.0705	2.4282	1	2.4282	2406	584.0466
889	269	1	2002	3	38	10	368.4259	218.096	5.1	2.4282	0.8094	0	0	2051	756.6801
890	269	2	2004	3	40	10	0	385.8572	4.3	4.8564	2.0235	1	1.6188	3845	791.1061
891	269	3	2006	3	42	10	0	641.5449	3.3	2.4282	3.2376	1	3.2376	3848	1028.503
892	269	4	2008	3	44	10	0	688.4933	4.1	4.047	3.2376	1	3.2376	2937	1092.031
893	270	1	2002	3	55	10	0	213.4592	1.8	4.047	2.4282	0	0	3161	1282.489
894	270	2	2004	3	57	10	1105.278	503.8427	1.8	4.8564	4.047	1	2.0235	3820	1150.692
895	270	3	2006	3	59	10	0	289.3186	3.6	4.047	4.047	1	4.047	2190	489.9145
896	270	4	2008	3	61	10	9.210648	558.3605	1.8	3.2376	0.8094	1	0.8094	3950	1187.693
897	271	1	2002	3	55	7	442.1111	262.1584	4	2.4282	1.6188	0	0	2906	1046.726
898	271	2	2004	3	57	7	0	192.3602	3.1	2.8329	2.8329	0	0	2583	892.8594
899	271	3	2006	3	59	7	0	344.5469	3.3	2.0235	1.6188	0	0	2900	1152.864
900	272	1	2002	3	50	15	0	261.0751	1.8	4.047	2.4282	0	0	3434	1141.765
901	272	2	2004	3	52	15	0	386.5848	1.8	4.65405	3.03525	1	0.8094	2841	886.8395
902	272	3	2006	3	54	15	0	247.5377	2.6	4.8564	3.2376	1	3.2376	2781	799.1614
903	272	4	2008	3	56	15	3684.259	537.6285	1.8	3.2376	3.2376	1	3.2376	3992	1345.958
904	273	1	2002	3	40	10	0	147.8211	4.2	5.2611	0.8094	0	0	2505	578.8832
905	273	2	2004	3	42	10	18.4213	228.9222	4.2	4.047	2.4282	1	2.4282	2527	280.9032
906	273	3	2006	3	44	10	0	597.6613	3.3	4.047	3.2376	1	3.2376	2877	460.4571
907	273	4	2008	3	46	10	165.7917	770.707	3.3	5.2611	4.047	1	4.047	3461	851.4407
908	274	1	2002	3	58	7	368.4259	238.6606	4.4	3.43995	1.2141	0	0	2459	636.0199
909	274	2	2004	3	60	7	0	439.7169	3.6	4.047	2.0235	1	0.8094	4695	1405.911
910	274	4	2008	3	64	7	773.6944	507.9803	5.2	4.8564	2.0235	1	2.0235	2864	687.0081
911	275	1	2002	3	52	7	331.5833	663.849	2.4	2.8329	3.6423	1	0.8094	3785	1519.843
912	275	2	2004	3	54	7	0	766.7879	2.6	6.0705	4.4517	1	0.8094	4626	2067.217
913	275	3	2006	3	56	7	0	513.5147	2.6	4.4517	4.8564	1	4.8564	3442	597.171
914	275	4	2008	3	58	7	0	625.5954	1.8	4.4517	1.82115	1	1.82115	4482	1355.143
915	276	1	2002	3	54	12	265.2667	863.6849	2.4	12.141	3.2376	1	2.0235	4147	1689.616
916	276	2	2004	3	56	12	792.1157	641.2385	2.6	8.094	2.0235	1	1.6188	3945	1425.902
917	277	1	2002	3	45	4	0	283.2581	4.7	2.4282	1.6188	1	0.4047	2386	576.9747
918	277	2	2004	3	47	4	0	1198.249	3.2	2.4282	1.6188	1	0.8094	3174	926.5245
919	277	3	2006	3	49	4	0	445.5318	3.9	2.22585	1.6188	1	1.6188	2856	357.2011
920	277	4	2008	3	51	4	0	1933.041	1.8	2.22585	1.82115	1	1.82115	4226	1074.548
921	278	1	2002	3	40	7	3223.745	576.4465	2.6	4.047	2.4282	1	1.6188	4573	1176.25
922	278	2	2004	3	42	7	0	937.1532	4.6	3.2376	2.0235	1	0.8094	4546	1691.521
923	278	3	2006	3	44	7	0	584.0566	3.6	3.6423	2.4282	1	2.4282	4623	1887.296

924	279	1	2002	3	65	0	0	174.8051	4	0.8094	0.8094	0	0	3092	1188.148
925	279	2	2004	3	67	0	27.63194	311.8387	4.2	0.8094	0.8094	1	0.8094	3295	1090.337
926	279	3	2006	3	69	0	184.213	321.673	3.8	0.8094	0.8094	1	0.8094	3181	813.7531
927	280	1	2002	3	50	7	276.3194	384.0972	3.6	2.8329	0.60705	0	0	2443	755.0144
928	280	2	2004	3	52	7	0	1066.876	3.6	2.8329	1.82115	1	1.82115	3885	1173.479
929	280	4	2008	3	56	7	0	1746.929	3.5	1.6188	1.6188	1	1.6188	2807	573.1037
930	281	1	2002	3	22	7	0	225.954	1.8	2.0235	1.6188	0	0	2270	551.1939
931	281	2	2004	3	24	7	0	223.5713	1.8	1.6188	1.41645	1	0.8094	4062	1051.416
932	281	3	2006	3	26	7	276.3194	316.9103	2.8	4.047	3.2376	1	3.2376	3569	628.6216
933	281	4	2008	3	28	7	0	389.3833	2.8	2.0235	1.2141	1	1.2141	4191	980.7092
934	282	1	2002	3	38	4	0	393.1736	4.4	3.2376	1.6188	1	0.8094	4364	1533.118
935	282	2	2004	3	40	4	0	535.9482	2.8	1.6188	1.2141	1	1.6188	4171	928.4413
936	282	3	2006	3	42	4	0	298.0886	1.8	3.2376	2.4282	1	2.4282	3463	712.8525
937	283	1	2002	3	50	12	0	363.7259	2.8	7.2846	3.2376	0	0	3379	1264.806
938	283	2	2004	3	52	12	0	1005.002	3.8	6.0705	4.8564	1	3.15666	2473	645.7148
939	283	3	2006	3	54	12	0	309.0623	3.4	7.2846	6.0705	1	1.2141	3070	621.0613
940	283	4	2008	3	56	12	1842.13	666.7084	3.6	6.0705	2.4282	1	2.4282	3204	954.245
941	284	1	2002	3	33	8	110.5278	523.8921	3.2	1.2141	0.8094	0	0	3023	870.977
942	284	2	2004	3	35	8	221.0555	291.86	7.5	2.0235	1.41645	1	1.2141	2435	463.9094
943	284	4	2008	3	39	8	736.8518	1938.163	2.4	1.2141	1.01175	1	1.01175	4483	911.7722
944	285	1	2002	3	48	7	0	157.9155	4	3.6423	2.0235	0	0	2502	875.0905
945	285	2	2004	3	50	7	0	238.4114	5	3.6423	2.0235	1	1.2141	3056	391.4698
946	285	3	2006	3	52	7	0	321.4572	3.4	3.6423	2.22585	1	2.22585	3166	499.6297
947	285	4	2008	3	54	7	0	493.9083	4.3	3.6423	2.4282	1	2.4282	2935	552.6802
948	286	1	2002	3	35	7	184.213	249.5691	2.5	2.4282	1.2141	1	0.4047	3863	914.0146
949	286	2	2004	3	37	7	0	208.5323	3.5	2.4282	1.41645	1	0.8094	3154	546.9063
950	287	1	2002	3	45	0	184.213	135.7407	4.9	2.4282	1.2141	0	0	2319	675.68
951	287	2	2004	3	47	0	18.4213	180.7367	4	2.4282	1.2141	1	0.8094	3514	768.5071
952	287	3	2006	3	49	0	0	176.5505	4.2	2.4282	1.6188	1	1.6188	3019	407.6648
953	287	4	2008	3	51	0	0	316.7335	3.5	2.4282	1.6188	1	1.6188	2593	317.3609
954	288	1	2002	3	48	2	265.2667	488.7156	3.6	1.2141	1.6188	0	0	3497	1334.715
955	288	2	2004	3	50	2	36.84259	910.7322	1.8	1.2141	1.2141	1	0.4047	4081	1628.7
956	288	3	2006	3	52	2	184.213	335.0933	4.2	0.8094	0.4047	1	0.4047	2433	308.2865
957	288	4	2008	3	54	2	736.8518	358.7115	3.4	1.2141	1.2141	1	1.2141	3526	896.2396
958	289	1	2002	3	50	2	0	264.2758	4.6	2.4282	0.8094	1	0.8094	2154	344.3464
959	289	2	2004	3	52	2	36.84259	3847.112	1.8	2.0235	1.6188	1	1.2141	3257	1244.938
960	289	3	2006	3	54	2	0	276.0063	5	2.4282	2.4282	1	2.4282	2483	790.9405
961	289	4	2008	3	56	2	552.6389	380.6568	4.2	2.4282	1.2141	1	1.2141	2184	594.1577
962	290	1	2002	3	60	12	0	105.7694	4.4	5.2611	1.6188	0	0	2310	713.7591
963	290	2	2004	3	62	12	0	505.7602	4	6.0705	1.6188	1	0.8094	3493	1185.937
964	290	3	2006	3	64	12	0	512.3634	4.4	3.6423	2.8329	1	2.8329	3746	1034.36
965	290	4	2008	3	66	12	368.4259	456.1616	5.6	0.8094	0.8094	1	0.8094	3084	965.3229
966	291	1	2002	3	50	12	0	277.0209	5.4	6.8799	2.4282	0	0	2964	1118.289
967	291	2	2004	3	52	12	18.4213	389.286	4.6	2.0235	1.6188	1	0.8094	3387	1361.911

968	291	3	2006	3	54	12	0	577.794	3.4	4.4517	2.4282	1	2.4282	3801	1200.913
969	291	4	2008	3	56	12	1105.278	685.4049	4.2	3.2376	3.2376	1	3.2376	2953	602.3585
970	292	1	2002	3	52	17	1989.5	1473.287	2.4	3.43995	2.0235	1	0.4047	3773	1435.399
971	292	2	2004	3	54	17	2210.555	4227.604	1.6	2.8329	1.82115	0	0	3910	1558.676
972	292	3	2006	3	56	17	1149.489	1182.1	3.2	4.8564	2.4282	1	2.4282	4528	1657.13
973	293	1	2002	3	45	12	5415.861	681.986	2.4	3.2376	1.2141	0	0	3474	760.9075
974	293	2	2004	3	47	12	552.6389	916.985	3.4	3.2376	2.0235	1	2.0235	3313	897.3433
975	293	3	2006	3	49	12	4789.537	793.8685	4.9	3.2376	2.0235	1	2.0235	4516	1256.448
976	293	4	2008	3	51	12	2026.342	1981.9	1.8	3.2376	0.4047	1	0.4047	4246	1325.827
977	294	1	2002	3	40	0	0	86.01969	5.4	1.6188	1.2141	0	0	1748	392.5162
978	294	3	2006	3	44	0	0	441.1064	3.8	1.6188	1.2141	1	1.2141	2885	598.5096
979	295	1	2002	3	42	12	0	565.9417	5.2	2.0235	1.2141	1	0.4047	2555	628.7692
980	295	2	2004	3	44	12	0	721.578	3.6	1.82115	1.82115	1	1.82115	3448	779.893
981	295	3	2006	3	46	12	0	668.5256	5.2	2.0235	2.0235	1	2.0235	3020	463.2332
982	295	4	2008	3	48	12	0	1482.945	5.1	2.0235	2.0235	1	2.0235	2698	721.1877
983	296	1	2002	3	30	10	921.0648	161.5741	3.9	2.22585	1.2141	1	0.4047	2495	620.2361
984	296	2	2004	3	32	10	552.6389	303.4713	4.8	2.8329	1.82115	1	0.8094	2354	545.0007
985	296	4	2008	3	36	10	0	757.7004	5.1	3.2376	2.8329	1	2.8329	2528	704.5435
986	297	1	2002	3	40	0	0	450.3385	3.6	2.4282	1.6188	0	0	2233	572.4335
987	297	2	2004	3	42	0	0	234.925	4.4	3.2376	1.6188	1	1.2141	2931	806.7685
988	297	3	2006	3	44	0	0	303.6429	3.3	2.4282	1.6188	1	1.6188	3814	910.8305
989	298	1	2002	3	35	15	0	208.412	3.5	2.4282	1.2141	1	0.4047	2988	730.2805
990	298	2	2004	3	37	15	0	1774.466	3.4	3.6423	2.63055	1	1.82115	3718	1162.5
991	298	4	2008	3	41	15	0	1289.972	3.3	2.4282	2.22585	1	2.22585	3316	665.2598
992	299	1	2002	3	60	0	0	151.2105	2.5	0.20235	0.20235	0	0	2903	1015.905
993	299	2	2004	3	62	0	55.26389	649.7802	2.6	0.20235	0.20235	0	0	2445	605.2756
994	299	3	2006	3	64	0	0	334.9374	2.6	0.8094	0.4047	1	0.4047	3952	922.9104
995	300	1	2002	3	28	0	265.2667	199.5076	2.5	0.505875	0.303525	0	0	2104	587.6421
996	301	1	2002	3	30	3	0	180.6788	5.6	1.2141	0.8094	0	0	2164	676.2057
997	301	2	2004	3	32	3	41.44791	128.9807	5.6	0.8094	0.8094	1	0.4047	3045	360.0757
998	301	3	2006	3	34	3	110.5278	358.7318	4	1.01175	1.01175	1	1.01175	3271	692.33
999	301	4	2008	3	36	3	0	217.2403	5.9	1.01175	0.8094	1	0.8094	1892	354.9208
1000	302	1	2002	3	49	12	847.3796	263.0201	4.3	1.82115	1.01175	0	0	2188	597.5255
1001	302	2	2004	3	51	12	2210.555	1005.093	1.6	1.41645	1.01175	1	0.4047	4485	2114.641
1002	302	3	2006	3	53	12	2394.768	524.5896	3.2	1.41645	1.2141	1	1.2141	3694	943.4208
1003	302	4	2008	3	55	12	1105.278	1432.941	3.8	4.24935	1.01175	1	1.01175	2960	685.3939
1004	303	1	2002	3	50	3	0	299.5507	3.6	1.2141	0.8094	1	0.4047	3177	829.6617
1005	303	2	2004	3	52	3	110.5278	348.1583	1.8	0.8094	0.8094	1	0.8094	3480	669.5411
1006	303	4	2008	3	56	3	0	204.8884	6.3	1.6188	1.6188	1	1.6188	1599	345.6213
1007	304	1	2002	3	27	12	1473.704	421.3689	4.6	9.7128	3.2376	1	0.8094	3490	1153.999
1008	304	2	2004	3	29	12	2486.875	385.5389	4.6	8.094	4.8564	1	2.4282	2441	323.7786
1009	304	3	2006	3	31	12	921.0648	557.2446	5.4	8.094	4.8564	1	4.8564	2553	566.5172
1010	304	4	2008	3	33	12	322.3727	710.3911	3.6	8.094	4.8564	1	4.8564	3587	1099.744
1011	305	1	2002	3	42	10	685.2722	629.6841	2.6	2.0235	0.8094	0	0	3176	993.687

1012	305	2	2004	3	44	10	331.5833	667.1517	2.8	1.2141	0.8094	1	0.4047	3012	699.5751
1013	305	3	2006	3	46	10	442.1111	514.4479	3.6	2.0235	1.2141	1	1.2141	3933	1457.539
1014	305	4	2008	3	48	10	782.905	798.2786	2.6	3.2376	1.2141	1	1.2141	3994	741.2889
1015	306	1	2002	3	38	10	0	301.1079	3.3	2.8329	1.2141	0	0	2747	1083.399
1016	306	2	2004	3	40	10	0	319.236	3.3	3.2376	1.6188	1	0.8094	4000	1254.893
1017	306	3	2006	3	42	10	0	262.8648	4.4	3.2376	1.6188	1	1.6188	2542	429.0333
1018	307	1	2002	3	50	8	110.5278	94.50143	5.1	2.0235	0.8094	0	0	1776	324.2022
1019	307	2	2004	3	52	8	442.1111	287.1608	2.5	1.2141	0.8094	1	0.8094	4071	1019.499
1020	307	3	2006	3	54	8	0	287.5047	5.2	1.2141	0.8094	1	0.8094	2486	580.8798
1021	308	1	2002	3	27	10	0	118.9167	4.9	3.2376	1.6188	1	0.4047	2281	587.5201
1022	308	2	2004	3	29	10	14.73704	240.2607	4.9	2.4282	1.2141	1	0.60705	2888	803.7468
1023	308	3	2006	3	31	10	0	357.6434	4.3	2.4282	1.6188	1	1.6188	2536	441.3187
1024	308	4	2008	3	33	10	0	389.8554	5.1	2.4282	1.6188	1	1.6188	2146	498.1644
1025	309	1	2002	3	29	7	36.84259	146.0484	3	1.82115	0.4047	0	0	2241	704.0154
1026	309	2	2004	3	31	7	55.26389	164.5975	3.2	1.01175	0.8094	0	0	2074	435.3051
1027	309	3	2006	3	33	7	0	234.9899	3.2	1.01175	1.01175	1	1.01175	3680	710.9932
1028	309	4	2008	3	35	7	0	573.9534	5	1.01175	1.01175	1	1.01175	3774	1075.148
1029	310	1	2002	3	28	10	0	438.2585	3.6	3.2376	1.6188	0	0	2470	1050.254
1030	310	2	2004	3	30	10	0	625.7572	3.8	4.047	2.8329	1	2.0235	4485	2083.376
1031	310	3	2006	3	32	10	0	455.588	3.9	4.047	2.4282	1	2.4282	2858	647.8441
1032	310	4	2008	3	34	10	0	733.1161	3.1	1.6188	1.2141	1	1.2141	2793	1034.662
1033	311	1	2002	3	40	0	184.213	157.817	4.5	4.047	2.0235	1	0.4047	2774	828.8281
1034	311	2	2004	3	42	0	110.5278	1185.982	3.8	2.4282	1.2141	1	0.8094	4300	1924.196
1035	311	3	2006	3	44	0	368.4259	383.6654	4.2	2.8329	1.6188	1	1.6188	3500	691.8728
1036	311	4	2008	3	46	0	1473.704	589.3922	5	2.8329	2.4282	1	2.4282	2181	732.5977
1037	312	1	2002	4	35	8	0	182.8149	4.6	3.2376	0.4047	1	0.4047	3201	298.5701
1038	312	2	2004	4	37	8	64.47453	261.4901	4.9	4.8564	0.4047	1	0.4047	3219	1056.556
1039	313	1	2002	4	37	0	0	119.5404	5	1.41645	0.8094	1	0.4047	4233	530.2799
1040	313	2	2004	4	39	0	179.6076	210.8572	3.3	3.2376	0.4047	1	0.4047	2547	400.4102
1041	313	3	2006	4	41	0	331.5833	136.3841	3.5	1.41645	1.2141	1	1.2141	3776	208.9157
1042	313	4	2008	4	43	0	0	174.7806	3.3	2.8329	1.01175	1	1.01175	2182	790.7847
1043	314	1	2002	4	45	5	3315.833	617.8006	3.2	1.01175	0.4047	1	0.4047	3373	1018.13
1044	315	1	2002	4	47	8	0	309.7603	6.2	0.8094	0.4047	0	0	3148	848.1239
1045	315	2	2004	4	49	8	1105.278	815.5677	5.2	1.82115	0.4047	1	0.4047	4020	1182.571
1046	315	4	2008	4	53	8	3315.833	1759.706	2.4	0.9469979	0.4047	1	0.4047	4472	1346.282
1047	316	1	2002	4	43	8	442.1111	329.4337	3.2	1.112925	0.8094	0	0	3252	1211.785
1048	316	2	2004	4	45	8	681.5879	839.3315	3.3	2.8329	1.2141	1	0.4047	3521	1316.234
1049	316	3	2006	4	47	8	0	547.8975	3	2.8329	1.2141	0	0	3638	1329.028
1050	316	4	2008	4	49	8	663.1666	1036.626	3.3	2.22585	0.8094	1	0.8094	3023	1053.21
1051	317	1	2002	4	54	12	3094.778	810.5687	4.3	2.22585	0.8094	1	0.4047	4621	1645.85
1052	317	2	2004	4	56	12	13263.33	1735.88	2.3	2.8329	2.0235	0	0	4034	1511.425
1053	318	1	2002	4	56	0	0	278.6295	6.4	3.2376	1.82115	1	0.4047	3579	241.0692
1054	318	2	2004	4	58	0	11.23699	225.9493	6.5	3.2376	1.6188	1	1.2141	2540	659.7137
1055	318	3	2006	4	60	0	0	192.5536	6.2	1.01175	0.60705	0	0	1682	356.8298

1056	318	4	2008	4	62	0	9.210648	607.3402	4.1	2.22585	0.4047	1	0.4047	2331	549.9055
1057	319	1	2002	4	60	5	110.5278	570.0944	2.6	2.0235	0.8094	0	0	3124	1002.495
1058	319	2	2004	4	62	5	478.9537	468.8358	2.6	2.0235	0.4047	1	0.4047	3929	938.3485
1059	319	3	2006	4	64	5	1105.278	249.4716	3.9	1.41645	0.60705	1	0.60705	2755	613.9545
1060	319	4	2008	4	66	5	0	611.0972	2.8	1.41645	0.8094	1	0.8094	3688	838.0251
1061	320	1	2002	4	56	8	232.1083	392.8067	5	1.82115	0.4047	1	0.4047	4177	1035.076
1062	321	1	2002	4	52	15	4052.703	370.5868	6.4	3.6423	0.60705	1	0.60705	3186	785.2057
1063	322	1	2002	4	75	0	88.42222	207.0168	2.4	1.01175	0.303525	1	0.303525	2507	1285.827
1064	322	2	2004	4	77	0	267.1088	330.9459	0.8	0.16188	0.16188	0	0	3400	107.6387
1065	323	1	2002	4	63	0	0.6447453	425.5741	4.4	1.800915	0.32376	1	0.32376	3737	1414.521
1066	323	2	2004	4	65	0	92.10648	345.3615	3.3	1.2141	1.2141	1	0.4047	3164	1063.708
1067	324	1	2002	4	55	5	0	514.3209	4.3	1.922325	0.708225	1	0.4047	3906	1227.386
1068	324	2	2004	4	57	5	92.10648	434.1292	3.6	2.0235	0.8094	1	1.2141	4063	823.5371
1069	325	1	2002	4	67	5	0	261.7973	2.6	1.01175	0.4047	1	0.4047	3937	644.729
1070	325	2	2004	4	69	5	396.0578	271.3203	2.6	1.112925	0.20235	0	0	3412	878.8443
1071	326	1	2002	4	50	5	0	189.3229	5.3	2.4282	0.708225	1	0.303525	2807	448.088
1072	326	2	2004	4	52	5	893.4328	1086.892	3.4	2.529375	0.505875	1	0.4047	3554	934.3461
1073	326	3	2006	4	54	5	1326.333	445.6937	3.4	4.8564	0.4047	1	0.4047	3633	631.3589
1074	326	4	2008	4	56	5	3684.259	595.826	3.4	4.8564	0.4047	1	0.4047	4045	864.3801
1075	327	1	2002	4	62	5	486.3222	190.4473	2.5	0.505875	0.60705	0	0	3249	904.4277
1076	327	2	2004	4	64	5	0	751.8531	1.8	1.517625	0.8094	0	0	3316	959.4235
1077	327	3	2006	4	66	5	0	309.9168	1.8	0.60705	0.4047	1	0.4047	3191	693.0161
1078	327	4	2008	4	68	5	921.0648	632.0757	3.8	0.8094	0.4047	1	0.4047	3041	782.8047
1079	328	1	2002	4	53	11	1105.278	227.5815	4.2	6.0705	0.60705	1	0.60705	3958	1040.908
1080	328	3	2006	4	57	11	1105.278	432.2914	3.8	4.047	1.2141	1	1.2141	3906	1029.148
1081	329	1	2002	4	60	0	55.26389	276.709	5	2.0235	0.4047	0	0	2321	480.9885
1082	329	2	2004	4	62	0	221.0555	292.1479	2.2	2.0235	0.8094	0	0	3801	1266.42
1083	329	3	2006	4	64	0	0	333.056	1.8	2.0235	0.8094	1	0.4047	3207	788.1458
1084	329	4	2008	4	66	0	0	231.2645	5.7	2.0235	0.20235	1	0.20235	2058	353.8365
1085	330	1	2002	4	55	5	442.1111	462.6344	2.8	0	0.20235	1	0.20235	4604	1477.208
1086	330	2	2004	4	57	5	442.1111	158.4311	3.6	2.4282	1.01175	1	0.8094	2665	612.8459
1087	331	1	2002	4	60	8	442.1111	209.5014	3.4	1.6188	0.4047	0	0	2509	702.8082
1088	332	1	2002	4	51	7	0	561.5921	2.8	2.4282	0.20235	1	0.28329	3965	1340.147
1089	332	2	2004	4	53	7	18.4213	650.4092	2.8	1.6188	0.4047	1	0.4047	2912	804.1001
1090	332	3	2006	4	55	7	0	517.5399	2.6	2.8329	0.8094	1	0.8094	3499	402.8131
1091	333	1	2002	4	50	7	1145.805	562.3738	3.9	4.047	0.505875	1	0.505875	3882	1039.985
1092	333	2	2004	4	52	7	1381.597	545.2024	3.5	2.8329	0.72846	1	0.505875	2796	502.9749
1093	334	1	2002	4	27	10	442.1111	635.7407	2.6	1.6188	0.8094	0	0	2912	1182.146
1094	334	2	2004	4	29	10	184.213	234.186	3.6	2.0235	0.8094	0	0	2472	725.1992
1095	334	3	2006	4	31	10	773.6944	509.9318	2.6	2.4282	0.8094	1	0.4047	3656	929.7933
1096	334	4	2008	4	33	10	663.1666	2325.082	3.4	1.6188	0.4047	1	0.4047	2843	770.5963
1097	335	1	2002	4	75	0	1105.278	391.785	3.5	1.6188	0.60705	1	0.60705	3462	285.3945
1098	335	2	2004	4	77	0	854.7481	252.1866	4.9	1.6188	0.8094	0	0	2091	707.2155
1099	335	3	2006	4	79	0	1105.278	292.3319	2.6	1.6188	0.8094	1	0.8094	3439	414.8757

1100	336	1	2002	4	42	7	0	194.3168	1.8	0.60705	0.4047	0	0	3115	1194.153
1101	336	2	2004	4	44	7	184.213	1326.626	1.8	2.8329	1.2141	1	0.4047	4661	1868.976
1102	336	3	2006	4	46	7	0	174.0724	3.3	0.4047	0.4047	1	0.4047	3422	1119.149
1103	337	1	2002	4	60	5	884.2222	247.9401	3.4	0	0.4047	0	0	3149	747.2996
1104	338	1	2002	4	52	8	0	540.4719	5.1	3.43995	0.32376	1	0.32376	4082	1155.902
1105	338	2	2004	4	54	8	2505.296	872.5079	3.2	2.0235	0.4047	0	0	3097	792.6415
1106	339	1	2002	4	46	9	442.1111	373.9352	3.3	1.01175	0.60705	1	0.60705	3841	1803.197
1107	339	2	2004	4	48	9	663.1666	836.4256	2.8	1.01175	0.60705	1	0.20235	4468	1599.67
1108	340	1	2002	4	40	6	0	582.9504	3.1	3.2376	0.52611	1	0.52611	3160	968.0567
1109	340	2	2004	4	42	6	0	1215.68	2	4.047	0.60705	0	0	4070	1024.181
1110	341	1	2002	4	53	10	442.1111	223.9844	3.4	2.4282	1.2141	0	0	2447	811.1589
1111	341	2	2004	4	55	10	921.0648	469.8424	2.6	2.4282	2.0235	1	1.2141	3054	901.0867
1112	342	1	2002	4	48	4	331.5833	183.8999	3.8	0	1.6188	0	0	3302	1029.554
1113	342	2	2004	4	51	4	663.1666	376.3129	4.2	2.4282	1.2141	1	1.2141	3005	846.9789
1114	342	4	2008	4	55	4	1105.278	1184.307	2.4	0	0.60705	0	0	3908	1021.28
1115	343	2	2004	4	40	3	1178.963	381.91	3.8	1.2141	0.4047	1	0.8094	3831	964.6135
1116	343	3	2006	4	42	3	0	508.6575	2.5	1.2141	1.2141	1	1.2141	3229	980.5743
1117	343	4	2008	4	44	3	0	4900.125	1.8	1.2141	0.60705	1	0.60705	4419	2052.66
1118	344	2	2004	4	49	9	386.8472	323.8517	3.4	0.60705	0.24282	0	0	2698	757.0901
1119	344	4	2008	4	53	9	1326.333	1783.078	1.8	1.2141	0.4047	1	0.4047	3333	830.3111
1120	345	2	2004	4	49	10	276.3194	285.0955	3.4	3.03525	1.2141	1	0.4047	2129	346.668
1121	345	3	2006	4	51	10	0	275.7477	3.4	2.8329	0.4047	1	0.4047	3016	1111.289
1122	346	2	2004	4	52	10	1132.91	438.7267	4.4	8.094	2.4282	1	0.4047	2289	401.6224
1123	347	2	2004	4	47	9	1326.333	304.1671	5	0.8094	0.4047	1	0.4047	3130	721.7504
1124	347	3	2006	4	49	9	736.8518	289.952	5.2	0.60705	0.303525	1	0.303525	3523	1113.45
1125	347	4	2008	4	51	9	3684.259	721.576	5.2	0.60705	0.60705	1	0.60705	4552	1444.571
1126	348	2	2004	3	26	12	921.0648	481.3877	6.4	4.8564	2.4282	1	2.0235	3021	1078.729
1127	348	3	2006	3	28	12	0	864.4929	1.8	4.047	1.2141	1	1.2141	2740	763.0609
1128	349	2	2004	3	25	6	46.05324	198.7608	2.8	4.8564	3.6423	1	0.8094	3104	526.1723
1129	349	3	2006	3	27	6	0	410.2091	3.1	4.8564	3.6423	1	3.6423	3887	1074.506
1130	349	4	2008	3	29	6	0	479.08	3.3	2.0235	2.0235	1	2.0235	3877	792.7712
1131	350	2	2004	3	29	6	368.4259	229.7522	4.6	3.2376	1.01175	1	1.41645	3021	980.2462
1132	350	3	2006	3	31	6	0	472.2734	3.4	2.8329	1.2141	1	1.2141	3433	1043.263
1133	351	2	2004	3	35	8	92.10648	379.7067	3.2	2.529375	0.910575	0	0	3428	1411.925
1134	351	3	2006	3	37	8	0	695.0978	3.2	3.2376	1.2141	1	1.2141	4217	1231.492
1135	351	4	2008	3	39	8	0	857.0945	3.2	3.03525	0.60705	1	0.60705	3885	668.4133
1136	352	2	2004	3	24	11	55.26389	363.5289	4.6	3.6423	0.8094	1	0.8094	3378	570.8081
1137	352	3	2006	3	26	11	0	458.4933	4.6	4.047	0.8094	1	0.8094	3272	765.8133
1138	352	4	2008	3	28	11	0	471.5468	6.6	4.047	1.01175	1	1.01175	4020	928.5042
1139	353	2	2004	3	45	7	1105.278	1163.529	1.8	2.0235	1.01175	1	0.8094	3687	1407.24
1140	353	3	2006	3	47	7	552.6389	590.1507	4.2	2.0235	1.2141	1	1.2141	1816	582.4385
1141	353	4	2008	3	49	7	1289.491	783.7804	3.5	2.0235	1.2141	1	1.2141	2391	693.2402
1142	354	2	2004	3	52	10	1694.759	392.7278	4.8	8.094	3.2376	1	1.6188	3278	610.3232
1143	354	3	2006	3	54	10	1473.704	1091.372	2.6	6.0705	4.047	1	4.047	3246	1067.03

1144	354	4	2008	3	56	10	0	1085.772	5	12.141	5.6658	1	5.6658	3133	1027.404
1145	355	2	2004	3	50	10	33.15833	464.9841	2.4	4.8564	1.6188	1	0.8094	4688	1185.69
1146	355	3	2006	3	52	10	0	743.951	3.2	3.2376	2.8329	1	2.8329	3661	808.0007
1147	355	4	2008	3	54	10	0	1350.827	3.3	2.8329	2.8329	1	2.8329	3901	972.0544
1148	356	2	2004	3	57	4	442.1111	370.2137	1.8	5.2611	1.2141	0	0	3281	1500.843
1149	356	3	2006	3	59	4	0	188.3367	5.8	2.0235	2.8329	1	0.8094	1763	253.2656
1150	356	4	2008	3	61	4	921.0648	929.9072	4.9	4.047	3.6423	1	2.4282	3983	1369.819
1151	357	2	2004	3	28	12	589.4814	915.9618	8.1	10.1175	2.0235	1	0.4047	3717	1252.464
1152	357	3	2006	3	30	12	0	834.0004	6	9.7128	1.6188	1	1.2141	3717	1212.556
1153	357	4	2008	3	32	12	1915.815	896.7536	4.8	9.7128	3.2376	1	3.2376	2971	964.0866
1154	358	2	2004	3	40	15	810.537	868.6676	3.4	5.6658	1.6188	1	0.4047	3727	1242.469
1155	358	3	2006	3	42	15	0	1619.501	4.1	5.6658	2.0235	1	2.0235	2903	1174.553
1156	358	4	2008	3	44	15	0	2071.797	1.8	5.6658	3.2376	1	3.2376	3222	1240.752
1157	359	2	2004	3	26	0	44.21111	214.5521	2.4	1.82115	1.01175	1	0.8094	3278	1386.979
1158	359	3	2006	3	28	0	0	328.3674	3.5	1.2141	1.6188	1	1.6188	3538	1072.748
1159	360	2	2004	2	39	15	0	469.9409	3.8	28.329	1.6188	1	0.8094	4288	628.1846
1160	360	3	2006	2	41	15	0	417.8637	3.8	8.094	4.047	1	4.047	3659	768.6721
1161	360	4	2008	2	43	15	368.4259	658.6432	4.1	24.282	4.047	1	4.047	2797	503.0021
1162	361	2	2004	2	27	9	0	561.9373	1.8	4.8564	0.8094	1	0.8094	4028	460.818
1163	361	4	2008	2	31	9	0	312.7601	4.4	1.82115	0.4047	1	0.4047	3511	517.7372
1164	362	2	2004	2	35	0	92.10648	207.6171	8.5	4.8564	1.6188	0	0	2636	265.8628
1165	363	2	2004	2	35	3	0	287.2351	10.6	23.4726	1.2141	1	8.094	2669	224.1592
1166	363	3	2006	2	37	3	0	776.5235	4.3	12.9504	1.6188	1	1.6188	4577	652.8655
1167	363	4	2008	2	39	3	1537.257	451.5518	11.1	12.141	1.2141	1	1.2141	2273	305.7812
1168	364	2	2004	2	49	4	0	252.2055	3.7	3.2376	1.2141	0	0	3224	530.8381
1169	365	2	2004	2	51	15	0	453.2742	3.4	9.3081	0.505875	0	0	3036	555.2141
1170	365	3	2006	2	53	15	0	300.2586	3.9	3.2376	0.8094	1	0.8094	3517	846.2877
1171	365	4	2008	2	55	15	0	220.9899	6.3	4.047	1.2141	1	1.2141	3747	397.7252
1172	366	2	2004	2	35	4	0	243.8563	4.2	4.8564	3.2376	0	0	1529	280.9741
1173	367	2	2004	2	42	12	0	129.1132	3.9	6.4752	0.8094	0	0	2691	489.1372
1174	367	4	2008	2	46	12	464.2166	202.0843	6.1	7.6893	1.2141	1	1.2141	3552	173.4348
1175	368	2	2004	2	30	9	0	319.2178	3.2	2.0235	0.8094	0	0	2097	729.5359
1176	370	2	2004	2	48	6	368.4259	536.6985	4.5	8.094	6.0705	1	1.6188	2441	250.5936
1177	371	2	2004	2	48	19	2431.611	1739.882	11.3	36.423	16.188	0	0	4233	582.6122
1178	371	3	2006	2	50	19	11052.78	1682.37	12.1	32.376	16.188	1	16.188	8469	2169.328
1179	371	4	2008	2	52	19	16063.37	5096.265	12.6	36.423	16.188	1	16.188	7928	1934.718
1180	372	2	2004	2	30	0	0	148.1987	7.4	1.2141	1.2141	0	0	2546	599.7051
1181	372	4	2008	2	34	0	0	207.4288	5.1	1.2141	3.2376	1	3.2376	2776	292.1667
1182	373	2	2004	2	50	0	0	146.703	6.9	6.4752	1.6188	0	0	3407	226.4716
1183	374	2	2004	2	35	0	0	138.3083	8.1	4.047	2.0235	0	0	1643	483.6464
1184	374	3	2006	2	37	0	0	212.9526	3.9	3.2376	2.0235	1	0.8094	2534	428.0707
1185	374	4	2008	2	39	0	92.10648	157.2524	4	0.8094	0.4047	1	0.4047	3188	224.2158
1186	375	2	2004	2	45	0	0	236.9259	9.2	9.3081	4.047	0	0	2677	583.4843
1187	376	2	2004	2	35	10	0	164.5553	5.3	3.2376	0.8094	0	0	3595	325.776

1188	377	2	2004	2	43	4	0	396.4933	9.1	20.235	1.6188	0	0	3023	750.9753
1189	378	2	2004	2	65	0	0	406.2346	12.4	16.188	8.094	1	3.2376	4395	549.4248
1190	378	3	2006	2	67	0	0	792.0846	16.6	16.188	10.1175	1	10.1175	3851	1069.956
1191	378	4	2008	2	69	0	3315.833	884.0812	16.2	16.188	8.094	1	8.094	3830	398.4999
1192	379	2	2004	2	50	0	0	190.0214	9.6	6.0705	2.4282	0	0	1589	433.8412
1193	379	3	2006	2	52	0	0	296.9867	3.4	4.047	2.0235	0	0	3205	607.2169
1194	379	4	2008	2	54	0	0	483.279	1.8	4.047	2.8329	1	2.8329	3496	790.8678
1195	380	2	2004	2	32	15	0	472.5785	4.6	20.235	4.047	0	0	3057	459.529
1196	380	4	2008	2	36	15	0	298.546	5.2	24.282	4.047	1	4.047	2913	850.6196
1197	381	2	2004	2	45	7	221.0555	186.3574	4.5	4.4517	1.2141	0	0	3760	796.7327
1198	381	3	2006	2	47	7	0	436.3262	5.8	8.4987	1.6188	0	0	3006	1027.923
1199	381	4	2008	2	49	7	1289.491	567.636	5.5	8.4987	3.2376	1	3.2376	3371	990.4015
1200	382	2	2004	2	50	0	0	227.2396	5.3	7.6893	1.6188	0	0	3530	737.7431
1201	382	3	2006	2	52	0	0	257.414	4.5	1.6188	1.6188	1	1.6188	3444	630.7848
1202	382	4	2008	2	54	0	921.0648	359.0541	5.1	1.6188	1.2141	1	1.2141	3003	217.2336
1203	383	2	2004	2	28	5	0	180.2334	4.6	6.0705	0.4047	0	0	2631	449.0124
1204	384	2	2004	2	75	5	0	330.6048	6	7.2846	1.6188	1	2.4282	4418	451.3606
1205	384	3	2006	2	77	5	0	364.7403	4.3	2.8329	1.01175	1	1.01175	3303	749.1586
1206	384	4	2008	2	79	5	0	479.3582	4.5	2.8329	1.01175	1	1.01175	4039	721.0015
1207	385	2	2004	2	64	7	0	302.0009	5.3	3.2376	1.2141	1	2.0235	3693	620.7045
1208	385	3	2006	2	66	7	0	976.8833	1.8	4.4517	2.4282	1	2.4282	3880	744.8676
1209	385	4	2008	2	68	7	0	571.4632	5.3	4.047	3.2376	1	3.2376	3121	665.3144
1210	386	2	2004	2	35	10	0	236.168	4.8	1.319322	0.833682	1	1.2141	3209	418.4948
1211	387	2	2004	2	75	7	221.0555	454.9886	4.8	5.46345	1.2141	1	2.0235	3481	249.6262
1212	387	3	2006	2	77	7	0	305.2845	3.2	2.22585	3.2376	1	3.2376	4057	362.2142
1213	387	4	2008	2	79	7	0	494.9934	5.2	2.10444	2.8329	1	2.8329	4183	223.2449
1214	388	2	2004	2	51	3	3679.027	681.3824	4.4	1.41645	0.60705	1	0.60705	3807	699.5602
1215	388	3	2006	2	53	3	0	280.6611	3.8	1.2141	0.4047	1	0.4047	3459	429.8944
1216	388	4	2008	2	55	3	66.31667	484.8646	3	1.41645	0.8094	1	0.8094	4282	611.4102
1217	389	2	2004	2	40	10	0	173.1696	4.3	2.0235	0.8094	1	0.8094	2638	266.8496
1218	389	4	2008	2	44	10	0	169.7418	3.5	0.4047	3.03525	1	3.03525	2515	230.8221
1219	390	2	2004	2	40	1	0	112.9734	5.9	1.6188	1.6188	1	0.8094	2358	264.9439
1220	391	2	2004	1	42	7	442.1111	260.5048	3.4	0.4047	2.4282	1	0.8094	3954	231.1095
1221	391	3	2006	1	44	7	0	197.5624	4	3.2376	2.4282	1	2.4282	2629	241.0906
1222	391	4	2008	1	46	7	0	314.8575	4.6	2.8329	2.22585	1	2.22585	2289	689.5867
1223	392	2	2004	1	45	4	221.0555	146.9759	6.3	1.6188	1.6188	0	0	3045	516.8122
1224	392	3	2006	1	47	4	0	692.17	4.1	1.6188	1.6188	1	1.6188	3621	808.7023
1225	392	4	2008	1	49	4	0	589.705	2.6	1.517625	0.8094	1	0.8094	3953	397.1531
1226	393	2	2004	1	55	9	246.8454	198.6812	3.9	0.4047	0.4047	1	0.4047	3603	535.3256
1227	394	2	2004	1	50	10	663.1666	258.3538	4	1.2141	0.8094	1	0.8094	3863	1041.804
1228	394	3	2006	1	52	10	817.9055	250.0337	3.3	1.2141	0.8094	1	0.8094	4029	1332.691
1229	394	4	2008	1	54	10	773.6944	244.0336	3.1	1.2141	0.8094	1	0.8094	3178	875.2209
1230	395	2	2004	1	60	10	792.1157	242.8649	8.4	2.22585	1.2141	0	0	2001	321.4595
1231	395	3	2006	1	62	10	817.9055	189.9685	9.2	2.0235	0.8094	1	0.8094	2181	367.8686

1232	395	4	2008	1	64	10	2210.555	258.0597	9.3	2.076111	1.2141	1	1.2141	2378	437.8581
1233	396	2	2004	1	38	14	11052.78	147.0759	7.9	1.6188	1.2141	1	0.8094	4243	290.3914
1234	396	3	2006	1	40	14	0	160.8763	5	1.01175	0.4047	1	0.4047	2415	224.2047
1235	396	4	2008	1	42	14	3536.889	277.7221	5	1.2141	0.8094	1	0.8094	3136	337.1359
1236	397	2	2004	1	45	2	552.6389	66.63938	8.2	0.8094	0.8094	0	0	2204	174.3438
1237	397	3	2006	1	47	2	0	150.2226	8.3	0.505875	0.505875	1	0.505875	2753	98.51674
1238	398	2	2004	1	21	15	1842.13	419.9636	9.7	3.6423	0.8094	1	0.8094	3431	1124.615
1239	398	3	2006	1	23	15	2763.194	454.4487	10.7	2.8329	1.01175	1	1.01175	3906	165.681
1240	399	2	2004	1	26	10	276.3194	72.35107	5.4	1.41645	1.01175	1	0.8094	2766	81.43228
1241	399	4	2008	1	30	10	884.2222	269.8588	2.2	1.01175	0.8094	1	0.8094	3444	489.3815
1242	400	2	2004	1	52	10	184.213	291.3984	7.4	6.0705	4.047	1	0.8094	3809	600.942
1243	400	3	2006	1	54	10	221.0555	223.2966	8	6.0705	2.4282	1	2.4282	2675	664.1122
1244	400	4	2008	1	56	10	368.4259	328.8092	9.8	6.0705	3.2376	1	3.2376	2022	314.687
1245	401	2	2004	3	28	5	0	201.2603	5.3	5.6658	4.24935	1	1.2141	3722	912.2072
1246	401	3	2006	3	30	5	0	283.4795	4.1	2.0235	2.0235	1	2.0235	4114	1453.39
1247	401	4	2008	3	32	5	0	441.1047	4.6	3.6423	3.6423	1	3.6423	2887	821.343
1248	402	3	2006	1	36	10	884.2222	185.0011	4.9	3.2376	0.8094	1	0.8094	2275	503.7025
1249	402	4	2008	1	38	10	994.7499	380.2457	4.3	0	1.01175	1	1.01175	1954	516.8245
1250	403	3	2006	1	38	15	0	184.6638	5	2.0235	0.8094	1	0.8094	2582	171.2683
1251	403	4	2008	1	40	15	0	565.1417	3.3	1.01175	1.2141	1	1.2141	2841	660.2889
1252	404	3	2006	1	35	0	921.0648	220.9939	7.4	1.6188	0.4047	1	0.4047	3125	362.7036
1253	404	4	2008	1	37	0	0	391.3679	4.9	1.6188	0.60705	1	0.60705	3101	686.8373
1254	405	3	2006	1	35	10	257.8981	123.6264	4.6	1.6188	0.20235	1	0.20235	2293	238.3739
1255	405	4	2008	1	37	10	0	355.3188	4.8	1.41645	1.2141	1	0.8094	1895	363.1845
1256	406	3	2006	1	36	17	0	242.4016	7.3	9.3081	2.8329	1	2.8329	3825	360.6109
1257	406	4	2008	1	38	17	0	301.5459	4.6	9.3081	1.2141	1	1.2141	2233	482.6195
1258	407	3	2006	1	44	9	0	138.8014	7.1	1.82115	0.505875	1	0.505875	2255	320.2517
1259	407	4	2008	1	46	9	2763.194	344.6277	4	1.6188	0.4047	1	0.4047	3598	734.7567
1260	408	3	2006	1	45	10	0	229.76	6	2.8329	1.6188	1	1.6188	2556	291.2405
1261	409	3	2006	1	20	9	221.0555	225.2485	4.4	3.2376	3.2376	1	3.2376	3525	325.7063
1262	409	4	2008	1	22	9	1473.704	249.1163	6.2	3.2376	2.0235	1	2.0235	2497	362.4553
1263	410	3	2006	1	47	12	0	764.1669	3.6	4.047	2.0235	1	2.0235	4193	888.2094
1264	410	4	2008	1	49	12	0	726.4478	3.8	4.4517	4.047	1	4.047	2970	573.3004
1265	411	3	2006	1	22	8	0	668.6868	3.6	2.8329	2.4282	1	2.4282	2988	468.6132
1266	411	4	2008	1	24	8	0	270.91	4	2.8329	2.0235	1	2.0235	2218	457.9169
1267	412	3	2006	1	34	10	0	980.0615	5.1	14.5692	6.0705	1	6.0705	2334	626.3844
1268	412	4	2008	1	36	10	0	485.4099	5.2	14.1645	4.4517	1	4.4517	2401	725.2784
1269	413	3	2006	1	47	10	0	216.2507	5.4	0.8094	0.8094	1	0.8094	2288	319.2662
1270	413	4	2008	1	49	10	0	232.7651	6.2	2.4282	1.2141	1	1.2141	2140	358.7119
1271	414	3	2006	1	30	0	663.1666	125.6899	5.6	1.01175	0.4047	1	0.4047	1405	286.4707
1272	415	3	2006	1	52	7	0	298.7857	3.3	2.4282	2.4282	1	2.4282	2858	548.677
1273	415	4	2008	1	54	7	0	332.9155	3.4	2.4282	0.8094	1	0.8094	3776	743.8038
1274	416	3	2006	3	65	9	4421.111	351.9856	7.4	7.2846	4.8564	1	4.8564	3131	1160.696
1275	416	4	2008	3	67	9	0	1417.711	3.6	7.2846	2.8329	1	2.8329	4631	1293.341

1276	417	3	2006	3	40	10	0	502.1299	5.9	4.047	2.0235	1	2.0235	2757	665.7769
1277	417	4	2008	3	42	10	0	1739.734	3.6	4.047	2.0235	1	2.0235	3722	1034.852
1278	419	3	2006	3	65	4	0	188.2767	4.6	1.2141	1.2141	1	1.2141	3020	465.6715
1279	419	4	2008	3	67	4	0	151.3997	5.2	1.41645	1.41645	1	1.41645	2062	338.6297
1280	420	3	2006	3	55	12	921.0648	251.0277	4.6	7.2846	3.2376	1	3.2376	2137	707.3821
1281	420	4	2008	3	57	12	193.4236	476.3098	2.8	7.2846	3.2376	1	3.2376	3140	937.1439
1282	421	3	2006	3	35	0	1105.278	125.172	4.3	0.8094	0.8094	1	0.8094	2193	381.3465
1283	421	4	2008	3	37	0	0	770.3058	3.5	0.8094	2.0235	1	2.0235	3622	912.6622
1284	422	3	2006	3	40	4	442.1111	216.5235	3.9	2.0235	1.2141	1	1.2141	3605	800.733
1285	422	4	2008	3	42	4	92.10648	337.6686	3.9	2.0235	1.6188	1	1.6188	3047	685.1783
1286	423	3	2006	3	35	10	0	191.6798	3.8	1.2141	0.8094	1	0.8094	2763	740.21
1287	424	3	2006	3	50	7	0	409.2017	8.1	2.0235	1.2141	1	1.2141	2556	795.6865
1288	425	3	2006	3	28	0	0	360.3553	3.3	0.8094	0.60705	1	0.60705	4013	1478.209
1289	425	4	2008	3	30	0	0	258.348	3.4	0.8094	1.2141	1	1.2141	2027	277.1659
1290	426	3	2006	3	35	10	0	263.3236	3.4	1.2141	1.01175	1	1.01175	3256	733.0253
1291	427	3	2006	3	35	0	55.26389	159.6312	3.9	1.01175	0.8094	1	0.8094	2447	387.0345
1292	427	4	2008	3	37	0	0	262.903	3.9	1.2141	1.2141	1	1.2141	3202	523.8568
1293	428	3	2006	3	25	16	0	319.0578	3.6	2.4282	1.2141	1	1.2141	3270	1078.882
1294	429	3	2006	3	40	0	0	846.4433	2.7	0.4047	0.4047	1	0.4047	4435	907.0059
1295	430	3	2006	3	46	7	0	445.2215	3.4	2.0235	2.0235	1	2.0235	2898	472.561
1296	430	4	2008	3	48	7	0	1718.2	3.5	2.0235	1.01175	1	1.01175	2642	586.5106
1297	431	3	2006	3	35	0	0	207.9604	3.3	1.6188	1.2141	1	1.2141	2691	332.3138
1298	431	4	2008	3	37	0	55.26389	320.7019	4.4	1.6188	1.41645	1	1.41645	3036	727.931
1299	432	3	2006	3	35	12	0	325.5453	3.2	4.047	3.2376	1	3.2376	2200	507.1088
1300	432	4	2008	3	37	12	0	479.4811	3	5.2611	5.2611	1	5.2611	3662	1400.274
1301	433	3	2006	3	29	16	0	329.0983	3.2	4.8564	2.8329	1	2.8329	3236	453.6441
1302	433	4	2008	3	31	16	736.8518	495.287	3.3	4.047	2.4282	1	2.4282	3245	753.3624
1303	434	3	2006	3	60	0	0	748.4597	3.5	5.2611	2.63055	1	2.63055	3198	742.403
1304	434	4	2008	3	62	0	0	924.4736	3.6	4.8564	2.8329	1	2.8329	3052	898.1559
1305	435	3	2006	3	27	0	0	372.8056	2.5	2.0235	1.82115	1	1.82115	2808	900.359
1306	436	3	2006	3	27	10	0	578.8472	1.2	2.4282	3.6423	1	3.6423	4234	1593.757
1307	436	4	2008	3	29	10	0	464.8784	4.8	1.82115	2.4282	1	2.4282	3179	900.7279
1308	437	3	2006	3	50	10	0	323.1032	4.3	2.0235	1.2141	1	1.2141	3069	660.4601
1309	437	4	2008	3	52	10	110.5278	563.0985	4.3	2.0235	0.8094	1	0.4047	4197	587.5023
1310	438	3	2006	3	32	0	276.3194	180.7511	3.8	0.60705	0.60705	1	0.60705	3560	758.6618
1311	438	4	2008	3	34	0	1086.856	405.7759	3.9	1.01175	0.60705	1	0.60705	4138	734.5433
1312	439	3	2006	3	45	10	0	306.6967	4.2	0.20235	0.20235	1	0.20235	2616	315.4388
1313	440	3	2006	3	27	10	0	564.403	1	0.8094	0.8094	1	0.8094	3661	1082.912
1314	440	4	2008	3	29	10	0	405.3083	3.6	0.8094	1.6188	1	1.6188	3237	693.522
1315	441	3	2006	3	41	5	0	343.029	3.5	2.8329	0.8094	1	0.8094	3651	1249.985
1316	442	3	2006	3	47	18	0	726.7709	3.4	6.0705	4.047	1	4.047	3289	636.1514
1317	443	3	2006	2	35	0	0	177.5954	4.6	3.6423	0.8094	1	0.8094	2883	268.5436
1318	444	3	2006	2	45	0	0	382.4344	6	1.2141	1.2141	1	1.2141	4206	492.8568
1319	444	4	2008	2	47	0	497.375	526.0258	5.2	2.4282	0.20235	1	0.20235	4145	482.0578

1320	445	3	2006	2	65	16	3684.259	511.5583	3.2	28.329	16.188	1	16.188	3239	407.2787
1321	446	3	2006	2	70	0	0	422.7794	11.2	10.5222	1.6188	1	1.6188	2999	315.0876
1322	446	4	2008	2	72	0	0	1145.049	14.5	12.141	4.047	1	4.047	4295	663.8317
1323	447	3	2006	2	60	5	0	899.8105	5.8	8.094	0.8094	1	0.8094	4382	737.0115
1324	448	3	2006	2	35	0	0	161.2885	2.6	5.6658	2.8329	1	2.8329	2800	632.4077
1325	449	3	2006	2	36	0	0	119.9396	10.8	7.2846	0.8094	1	0.8094	998	237.1119
1326	450	3	2006	2	65	0	0	296.1187	10.8	6.0705	2.8329	1	2.0235	3150	742.8334
1327	450	4	2008	2	67	0	0	274.2486	7.2	2.4282	1.6188	1	1.6188	3754	457.9951
1328	451	3	2006	2	55	0	0	267.0204	4.2	4.8564	1.6188	1	1.6188	4025	398.2353
1329	451	4	2008	2	57	0	442.1111	676.34	4.2	4.8564	1.41645	1	1.41645	3010	634.0195
1330	452	3	2006	2	30	0	0	215.8373	3.9	1.01175	2.4282	1	2.4282	3124	330.3232
1331	453	3	2006	2	50	6	0	182.3551	4.9	2.4282	0.8094	1	0.8094	2680	152.8024
1332	453	4	2008	2	52	6	1289.491	499.6956	5.8	2.4282	1.2141	1	1.2141	3931	717.8179
1333	454	3	2006	2	45	0	0	216.7484	4	1.2141	0.60705	1	0.60705	3768	396.123
1334	454	4	2008	2	47	0	0	500.0351	4.2	1.6188	1.2141	1	1.2141	2655	430.2717
1335	455	3	2006	2	34	10	0	284.7762	3.1	4.047	0.8094	1	0.8094	3643	556.4048
1336	455	4	2008	2	36	10	884.2222	776.4252	10.1	2.0235	2.4282	1	2.4282	2468	354.1664
1337	456	3	2006	2	35	12	0	284.6301	3.1	6.4752	1.82115	1	1.82115	3077	734.7549
1338	456	4	2008	2	37	12	221.0555	770.9451	3.2	5.2611	4.047	1	4.047	3840	962.7006
1339	457	3	2006	2	59	8	0	344.1601	4.1	1.112925	1.112925	1	1.112925	2633	425.6174
1340	457	4	2008	2	61	8	27.63194	266.9842	4.3	0.8094	0.8094	1	0.8094	2796	214.4795
1341	458	3	2006	2	26	0	0	195.8624	2.6	0.60705	0.4047	1	0.4047	2783	274.1804
1342	458	4	2008	2	28	0	0	249.9248	3.4	0.8094	0.8094	1	0.8094	3367	865.8207
1343	459	3	2006	2	30	0	110.5278	239.7909	3.2	2.8329	0.8094	1	0.8094	3838	422.1053
1344	460	3	2006	2	18	0	0	217.9832	5.8	4.047	1.6188	1	1.6188	3366	570.6763
1345	460	4	2008	2	20	0	0	207.4992	5.2	4.047	3.6423	1	3.6423	2621	379.3792
1346	461	3	2006	2	20	0	0	539.4648	2.8	2.4282	2.0235	1	2.0235	4418	1618.682
1347	461	4	2008	2	22	0	31.3162	432.2737	10.4	2.4282	4.4517	1	4.4517	3433	1009.432
1348	462	3	2006	2	60	4	0	328.5811	4.6	9.7128	3.2376	1	2.0235	2486	392.5992
1349	462	4	2008	2	62	4	55.26389	304.9987	4.6	8.094	1.6188	1	1.6188	2954	510.8322
1350	463	3	2006	2	35	7	0	256.4276	13.3	8.094	0.8094	1	0.8094	1442	224.9729
1351	463	4	2008	2	37	7	1105.278	253.5523	11.9	8.094	2.4282	1	2.4282	2679	153.6652
1352	464	3	2006	2	35	0	0	151.1468	5.7	0.303525	0.4047	0	0	2300	368.832
1353	464	4	2008	2	37	0	0	227.2082	7	1.2141	1.6188	1	1.6188	3021	1244.297
1354	465	3	2006	2	40	0	0	360.063	4.9	4.047	0.8094	1	0.8094	4063	1025.884
1355	466	3	2006	2	25	10	0	528.6935	6.6	6.0705	4.047	0	0	2968	471.1331
1356	467	3	2006	2	58	0	0	295.2327	3.4	2.0235	0.60705	1	0.60705	2285	304.3891
1357	467	4	2008	2	60	0	313.162	184.5074	1.8	2.4282	0.4047	1	0.4047	3729	242.1243
1358	468	3	2006	2	22	10	0	220.4632	4.8	1.2141	1.2141	0	0	962	126.7447
1359	469	3	2006	2	21	0	0	295.1503	2.8	3.2376	3.2376	1	3.2376	2937	365.555
1360	469	4	2008	2	23	0	0	227.6636	3.8	3.2376	2.0235	1	2.0235	2296	387.1904
1361	470	3	2006	2	40	8	0	454.2688	4.6	3.6423	2.4282	1	2.4282	3590	376.1676
1362	470	4	2008	2	42	8	0	583.1035	4.9	3.6423	8.9034	1	8.9034	2773	624.8856
1363	471	3	2006	2	28	0	0	454.9809	3.1	8.094	8.094	1	8.094	3110	518.4613

1364	471	4	2008	2	30	0	0	445.0439	4	6.4752	4.8564	1	4.8564	3155	483.3039
1365	472	3	2006	2	32	0	147.3704	234.0679	3.3	0.4047	0.4047	1	0.4047	4022	613.6599
1366	472	4	2008	2	34	0	73.68518	330.6794	4.3	0.8094	0.8094	1	0.8094	2777	718.7899
1367	473	3	2006	2	55	0	0	323.472	4.8	24.282	3.2376	1	2.4282	2739	368.8568
1368	473	4	2008	2	57	0	331.5833	573.9355	6	24.282	6.0705	1	6.0705	4318	1886.945
1369	474	4	2008	2	52	7	22.10555	358.897	4.4	0.8094	1.6188	1	1.6188	3403	617.4888
1370	475	4	2008	2	58	4	27.63194	427.275	3.8	2.8329	2.4282	1	2.4282	3278	687.9355
1371	476	4	2008	2	28	7	276.3194	244.9194	4.6	0.8094	0.8094	1	0.8094	3506	347.1508
1372	477	4	2008	2	60	8	276.3194	484.7831	10.4	5.2611	4.047	1	4.047	3665	489.5972
1373	478	4	2008	2	50	0	0	557.6837	3.8	2.8329	2.8329	1	2.8329	3093	1266.562
1374	479	4	2008	2	58	3	0	360.742	3.6	2.4282	0.8094	1	0.8094	2531	266.8309
1375	480	4	2008	2	75	5	92.10648	214.9611	4.3	1.2141	0.60705	1	0.60705	2624	283.1981
1376	481	4	2008	2	45	6	1105.278	660.6769	4.5	7.2846	2.4282	1	2.4282	4267	326.0186
1377	482	4	2008	2	38	14	0	919.4334	11.3	24.282	12.141	1	12.141	4538	762.5833
1378	483	4	2008	2	40	8	0	305.0827	3.3	0.8094	1.2141	1	1.2141	3365	527.5524
1379	484	4	2008	2	49	11	1934.236	555.2219	11	4.047	0.8094	1	0.8094	2403	424.2146
1380	485	4	2008	2	42	10	88.42222	510.6794	3.3	6.27285	2.4282	1	2.4282	3474	1026.506
1381	486	4	2008	2	31	5	560.3758	216.6057	3.6	1.01175	0.4047	1	0.4047	3557	557.1697
1382	487	4	2008	2	26	15	884.2222	487.9794	3.4	0.8094	0.8094	1	0.8094	4443	981.9769
1383	488	4	2008	2	48	18	92.10648	1268.655	5	16.188	2.4282	1	2.4282	3405	889.0806
1384	489	4	2008	2	58	12	0	975.3322	5.2	24.282	1.2141	1	1.2141	3821	420.7017
1385	490	4	2008	2	45	6	176.8444	358.1449	4.4	2.8329	1.2141	1	1.2141	2363	403.7318
1386	491	4	2008	2	38	10	92.10648	268.1474	7.8	2.8329	1.2141	1	1.2141	4498	302.5461
1387	492	4	2008	2	48	8	0	260.6894	9	8.094	1.2141	1	1.2141	2543	562.4539
1388	493	4	2008	2	61	0	552.6389	165.689	7.7	0.8094	0.8094	1	0.8094	2684	233.8036
1389	494	4	2008	2	45	0	1197.384	296.8413	5.4	1.2141	1.6188	1	1.6188	2799	257.1441
1390	495	4	2008	2	55	4	0	276.89	6.2	4.8564	4.047	1	4.047	2914	925.3569
1391	496	4	2008	2	50	0	36.84259	161.5818	7.1	2.0235	2.0235	1	2.0235	1637	224.9263
1392	497	4	2008	2	30	4	0	228.284	3.2	1.6188	1.6188	1	1.6188	2652	426.6067
1393	498	4	2008	2	32	14	0	234.2692	3.1	2.0235	0.8094	1	0.8094	4285	446.4235
1394	499	4	2008	3	55	0	0	419.8044	3.6	4.047	3.2376	1	3.2376	4029	1306.314
1395	500	4	2008	3	38	8	0	717.9619	3.3	2.0235	1.6188	1	1.6188	4274	1012.569
1396	501	4	2008	3	26	0	0	637.2023	7.5	2.0235	4.047	1	4.047	3648	611.6432
1397	502	4	2008	3	47	5	0	385.4224	1.8	3.2376	1.01175	1	1.01175	2451	916.7576
1398	503	4	2008	3	42	5	193.4236	2718.075	1.8	7.2846	1.2141	1	1.2141	4484	1571.041
1399	504	4	2008	3	33	10	0	731.3947	2.8	0	0.8094	1	0.8094	3172	1651.035
1400	505	4	2008	3	24	12	0	878.165	3.6	3.6423	1.2141	1	1.2141	2901	1023.343
1401	506	4	2008	3	25	5	0	643.5197	2.8	4.047	2.8329	1	2.8329	4110	1181.623
1402	507	4	2008	3	39	7	0	966.4243	2.6	4.047	1.6188	0	0	3293	1176.157
1403	508	4	2008	3	40	12	921.0648	749.2166	3.5	0.8094	1.2141	1	1.2141	3302	610.3871
1404	509	4	2008	3	39	10	0	1332.402	3.5	3.2376	2.4282	1	2.4282	2347	677.8991
1405	510	4	2008	3	65	3	442.1111	1800.075	3.6	2.0235	1.6188	1	1.6188	3304	745.8597
1406	511	4	2008	4	50	0	442.1111	517.3916	3.6	1.2141	0.4047	0	0	2988	1050.99
1407	512	4	2008	4	56	5	663.1666	442.2788	3.5	1.6188	0.8094	1	0.8094	3573	848.8942

1408	513	4	2008	1	32	10	1842.13	458.9765	5.5	4.8564	2.0235	1	2.0235	3483	955.3231
1409	514	4	2008	1	42	17	1565.81	802.4846	7.1	12.141	4.8564	1	4.8564	3003	674.8392
1410	515	4	2008	1	24	8	644.7453	415.9438	2.8	0.8094	0.4047	1	0.4047	2842	663.9138
1411	516	4	2008	1	28	13	0	224.7072	5.3	3.6423	2.0235	1	2.0235	1774	423.0491
1412	517	4	2008	1	32	12	663.1666	220.7428	5.6	1.2141	0.8094	1	0.8094	2465	408.8392
1413	518	4	2008	1	52	4	0	652.0031	4.4	4.047	4.047	1	4.047	2682	1128.609
1414	519	4	2008	1	35	0	644.7453	302.1008	3.8	2.0235	1.2141	1	1.2141	3720	489.6805
1415	520	4	2008	1	26	8	0	356.731	3.6	1.2141	1.01175	1	1.01175	2735	976.7053
1416	521	4	2008	1	28	14	884.2222	1771.434	1.8	7.2846	2.8329	1	2.8329	2636	457.2859
1417	522	4	2008	1	50	0	0	332.2762	1.8	1.6188	1.2141	1	1.2141	3589	603.9155
1418	523	4	2008	1	43	15	2026.342	447.6756	7	14.1645	3.6423	1	3.6423	2988	366.4913
1419	524	4	2008	1	44	5	0	591.627	1	8.094	2.4282	1	2.4282	4374	685.7033
1420	525	4	2008	1	30	15	221.0555	278.5309	7.6	8.094	4.047	1	4.047	2962	483.0302
1421	526	4	2008	1	50	0	3278.99	367.987	6.3	0.8094	0.8094	1	0.8094	3029	348.0988
1422	527	4	2008	1	62	0	1400.018	183.5381	4.5	0.8094	0.505875	1	0.505875	2470	447.7822
1423	528	4	2008	1	48	10	0	563.3492	3.6	3.2376	3.2376	1	3.2376	2416	614.8389
1424	529	4	2008	1	40	9	0	519.2078	4.3	1.41645	1.2141	1	1.2141	2723	789.1661
1425	530	4	2008	1	48	7	0	490.8499	5.6	2.0235	2.0235	1	2.0235	2393	550.9553
1426	531	4	2008	1	35	10	0	310.3535	9.8	8.094	6.8799	1	6.8799	2359	249.5222
1427	532	4	2008	1	28	7	552.6389	193.5636	6.3	1.6188	0.60705	1	0.60705	2478	268.0789
1428	533	4	2008	1	40	10	2136.87	876.0759	2.3	2.0235	2.0235	1	2.0235	3873	875.3705
1429	534	4	2008	1	26	15	0	693.1562	3	16.188	10.1175	1	10.1175	3114	807.1349
1430	535	4	2008	1	50	3	0	233.7679	3.8	4.047	2.0235	1	2.0235	2434	308.9781
1431	536	4	2008	1	25	7	442.1111	140.3582	6.2	5.6658	2.8329	1	2.8329	2118	302.4287
